# C−N Axial Chiral Hypervalent Iodine Reagents: Catalytic Stereoselective α‐Oxytosylation of Ketones

**DOI:** 10.1002/chem.202005253

**Published:** 2021-02-08

**Authors:** Haifa Alharbi, Mohamed Elsherbini, Jihan Qurban, Thomas Wirth

**Affiliations:** ^1^ School of Chemistry Cardiff University Main Building, Park Place Cardiff CF10 3AT UK; ^2^ current address: Department of Chemistry University of Huddersfield Queensgate Huddersfield HD1 3DH UK; ^3^ current address: Department of Chemistry Faculty of Applied Science Umm Al-Qura University Makkah Saudi Arabia

**Keywords:** catalysis, hypervalent iodine, ketones, stereochemistry, α-oxytosylation

## Abstract

A simple synthesis of a library of novel C−N axially chiral iodoarenes is achieved in a three‐step synthesis from commercially available aniline derivatives. C−N axial chiral iodine reagents are rarely investigated in the hypervalent iodine arena. The potential of the novel chiral iodoarenes as organocatalysts for stereoselective oxidative transformations is assessed using the well explored, but challenging stereoselective α‐oxytosylation of ketones. All investigated reagents catalyse the stereoselective oxidation of propiophenone to the corresponding chiral α‐oxytosylated products with good stereochemical control. Using the optimised reaction conditions a wide range of products was obtained in generally good to excellent yields and with good enantioselectivities.

## Graphical abstract

**Novel C‐N axial chiral iodoarenes** enable catalytic stereoselective α‐oxytosylations of ketones with high yields and improved enantioselectivities.

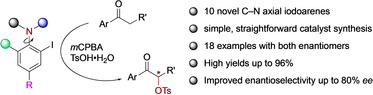

Hypervalent iodine compounds are very attractive in modern synthetic chemistry as they are environmentally and economically viable alternatives to transition metal reagents.^[1]^ Although the history of chiral hypervalent iodine reagents can be traced back to the seminal work by Pribram published in 1907,^[2]^ it took almost a century till they became active players in stereoselective synthesis.^[3]^ Nowadays, chiral hypervalent iodine reagents are widely used in a wide range of stereoselective transformations, including, but not limited to, stereoselective difunctionalisation of alkenes,^[4]^ α‐functionalisation of carbonyl compounds,^[5]^ oxidation of sulfur compounds,^[6]^ phenol dearomatisation,^[7]^ and oxidative rearrangements.^[8]^ In addition they are gaining an increased interest as redox‐active mediators in oxidative electrochemical transformations.^[9]^

Among the wide spectrum of chiral hypervalent iodine reagents and chiral iodoarene catalysts, axial chiral iodine‐containing scaffolds are very promising from structural and synthetic perspectives. Numerous enantioselective oxidative transformations have been achieved with high levels of stereocontrol using axial chiral hypervalent iodine reagents under stoichiometric and catalytic reaction conditions.^[3]^ The majority of axial chiral hypervalent iodine reagents and their iodoarene precursors contain a chiral C−C axis such as biphenyls **1**,^[10]^ binaphthyls **2** ^[11]^ or spiroindanes **3** ^[12]^ (Figure 1). On the other hand, axial chiral iodoarenes containing a chiral C−N axis such as **4** are rarely investigated in the context of hypervalent iodine chemistry. Hence, the synthesis of such compounds with a chiral C−N axis and the investigation of their potential in stereoselective oxidative transformations is of great interest. To the best of our knowledge, only one report on the synthesis and reactivity of C‐N axial chiral hypervalent iodine reagents emerged during the final preparation of this work.^[13]^

**Figure 1:**
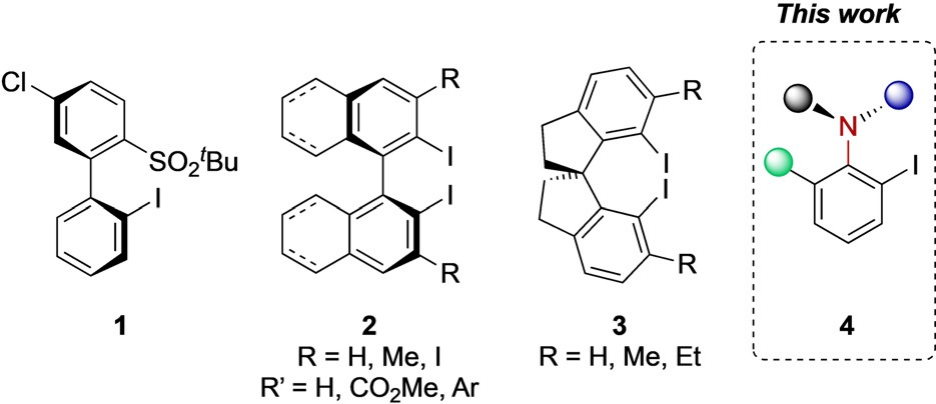
Examples of axially chiral iodoarene scaffolds.

Herein, we report a simple synthesis of a small library of novel C−N axial chiral iodoarenes, starting from commercially available aniline derivatives and investigate their potential as chiral organocatalysts using the extensively studied—yet challenging—hypervalent iodine mediated stereoselective α‐oxytosylation of ketones as a model reaction.[^5c^, ^5d^, ^14^]

In contrast to axial chiral biaryl systems, the methods available for the stereoselective construction of C−N axial chiral compounds are limited. As we are interested to develop a facile and rapid access to the target molecules, we want to avoid the use of specialised and/or complex or expensive catalysts and reagents. Therefore, our synthesis relies on chiral resolution to keep the synthetic route simple and to access optically active target molecules from simple and cheap commercially available chemicals. In addition, chiral resolution would enable access to diastereomers of each compound enabling a rapid construction of the target library of chiral iodoarenes.

The synthesis commences with the electrophilic iodination of anilines using molecular iodine^[8f]^ to give the corresponding iodoanilines **5 a** and **5 b** in 88 % and 40 % yield, respectively. Racemic iodosulfonamides **6 a**–**d** were obtained in good yields by treatment of iodoanilines **5** with the sulfonyl chloride derivatives, namely, *p*‐tosyl chloride (TsCl), *p*‐nosyl chloride (NsCl) and *p*‐anisylsulfonyl chloride (AnCl). Reaction of the racemic mixtures **6 a**–**d** with (*S*)‐lactate esters under Mitsunobu reaction conditions^[8f]^ led to the formation of the corresponding diastereomeric mixtures of **7** that were easily separated by crystallisation or column chromatography. The reaction led to the formation of the *S*_C‐N_ diastereomer as major isomer and the *R*_C‐N_ diastereomer as the minor isomer in all cases with the *de* ranging from 10 % to 30 %. As a result, ten novel optically active C−N axial chiral iodoarenes (**7 a**–**j**) were synthesised in satisfactory yields over three simple chemical steps (Scheme 1).

**Scheme 1:**
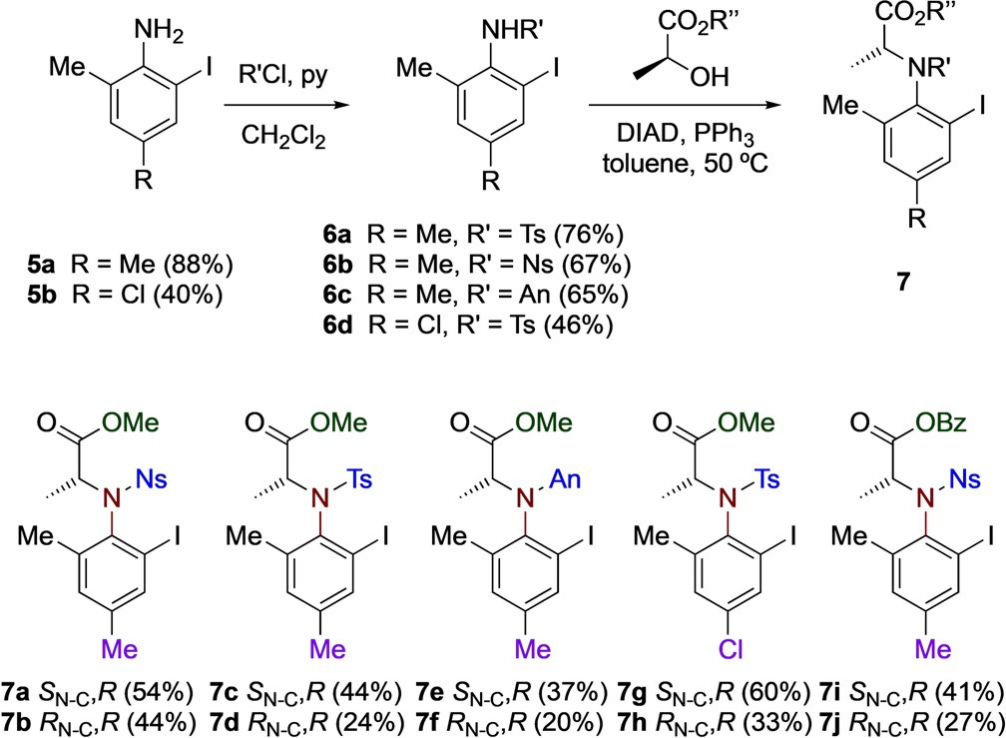
Synthesis of novel C‐N axial chiral iodoarenes **7 a**–**j**. Ts: 4‐toluenesulfonyl; Ns: 3‐nitrobenzenesulfonyl; An: 4‐methoxybenzenesulfonyl.

The absolute configuration of the iodoarenes **7** were assigned through analysis of the X‐ray crystallographic structures.^[15]^ The 3D structures of the diastereomers **7 a** and **7 b** are shown in Figure 2 while other X‐ray structures (**7 d**, **7 e**, **7 h**) are found in the supporting information.

**Figure 2:**
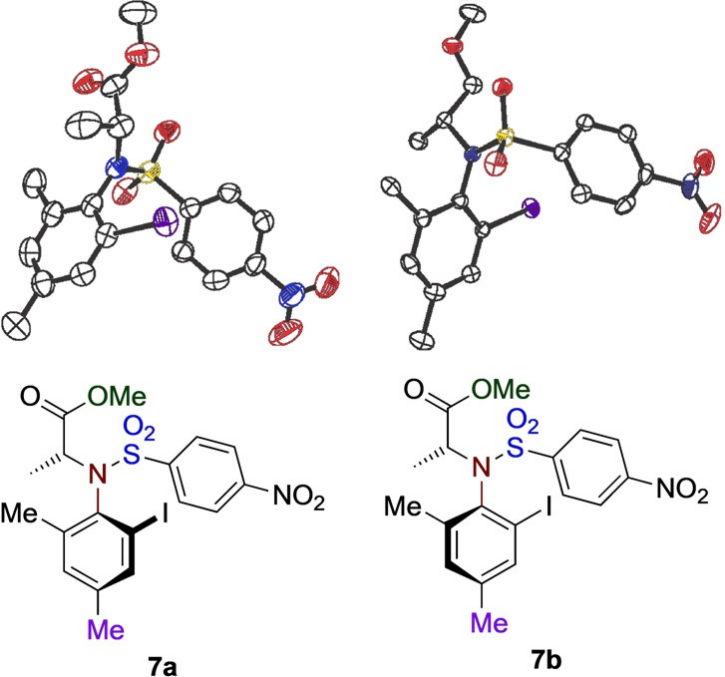
3D structure and absolute configuration of diastereomers **7 a** and **7 b**.

After the library synthesis of C−N axial chiral iodoarenes, their potential as organocatalysts for the stereoselective α‐oxytosylation of ketones was initially probed using propiophenone **8 a** as a model substrate, *m*‐chloroperbenzoic acid (*m*CPBA, 3 equiv) as the terminal oxidant and *p*‐toluenesulfonic acid (TSA, 3 equiv) as nucleophile, according to a literature procedure.^[14b]^ The result of the catalyst screening (Table 1) shows that catalytic amounts (10 mol %) of all iodoarenes **7** led to the formation of the desired product **9 a** in good to excellent yields and only with pre‐catalyst **7 j** (entry 10) a low yield was obtained. The enantiomeric excess of the resulted α‐tosyloxy ketone **9 a** was moderate to good (31–67 % *ee*) in all cases except for pre‐catalysts **7 i** (9 % *ee*, entry 9) and **7 g** (21 % *ee*, entry 7). The best results were obtained using pre‐catalyst **7 d** (entry 4) where (*S*)‐**9 a** was formed in excellent yield (96 %) and with good enantioselectivity (67 % *ee*). On the other hand, pre‐catalyst **7 c** gave the best results for the opposite enantiomer (*R*)‐**9 a**. Importantly, the configuration of the stereogenic centre of **9 a** seems solely depending on the configuration of the chiral C−N axis where the *R*_C‐N_ configuration always forms **9 a** with (*S*)‐configuration. This proves that the stereochemical induction is mainly controlled by the chiral axis and not by the stereocentre in the lactate moiety.

**Table 1:** Screening of pre‐catalysts **7** in the enantioselective α‐tosyloxylation of acetophenone **8 a**.^[a]^

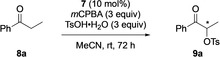				
Entry	Ar−I reagent	**9 a** Yield [%]	**9 a** *ee* [%]^[b]^	**9 a** abs. config.
1	**7 a**	40	47	*R*
2	**7 b**	85	40	*S*
3	**7 c**	93	43	*R*
4	**7 d**	96	67	*S*
5	**7 e**	81	31	*R*
6	**7 f**	78	59	*S*
7	**7 g**	45	21	*R*
8	**7 h**	62	64	*S*
9	**7 i**	53	9	*R*
10	**7 j**	20	55	*S*

[a] Reactions were carried out with 0.027 mmol of **7**, 0.27 mmol of **8 a**, 0.81 mmol of *m*CPBA and 0.81 mmol of TsOH in acetonitrile (5 mL) at room temperature for 72 h. [b] Enantiomeric excesses were determined by chiral‐phase HPLC analysis.

Using pre‐catalyst **7 d**, the influence of other reaction parameters, such as solvent, stoichiometries of the terminal oxidant and *p*‐toluenesulfonic acid, and temperature were studied (Table 2). Performing the reaction at 0 °C (entry 2) lead to a slight increase in enantioselectivity (70 % *ee*) but was accompanied by a significant reduction in yield (26 %). On the other hand, increasing the reaction temperature to 50 °C (entry 3) did not show a significant change in the reaction outcome. Decreasing the amount of *m*CPBA and *p*‐toluenesulfonic acid from 3 to 2 equivalents (entry 4) did not affect the yield, but led to a reduced *ee* of 52 % while using 5 equivalents (entry 5) had no effect. Subsequent results show that the most effective reaction parameter is the solvent. The reaction performed in ethyl acetate did not perform well (entry 8), but with dichloromethane and diethyl ether an improved enantioselectivity of 73 % and 76 % *ee* was observed albeit with much smaller yields (entries 6, 7). Using mixtures of acetonitrile and dichloromethane (entries 9–11) was more efficient compared to using any of the two solvents on their own. The best result, 94 % yield and 75 % *ee* of **9 a** was obtained using a 1:1 mixture. On the other hand, the best results for using the diastereomeric pre‐catalyst **7 c** [(*R*)‐**9 a**: 72 % yield, 75 % *ee*, entry 12] were obtained with a 1:1 mixture of EtOAc and CH_2_Cl_2_ (see supporting information, Table S1).

**Table 2:** Screening of reaction parameters.^[a]^

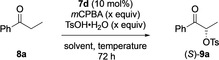					
Entry	Solvent	*x* [equiv]	Temp. [°C]	**9 a** Yield [%]	**9 a** *ee* [%]^[a]^
1	MeCN	3	20	96	67
2	MeCN	3	0	26	70
3	MeCN	3	50	94	66
4	MeCN	2	20	92	52
5	MeCN	5	20	96	64
6	CH_2_Cl_2_	3	20	70	73
7	Et_2_O	3	20	40	76
8	EtOAc	3	20	64	56
9	MeCN‐CH_2_Cl_2_ (1:1)	3	20	94	75
10	MeCN‐CH_2_Cl_2_ (1:2)	3	20	90	73
11	MeCN‐CH_2_Cl_2_ (2:1)	3	20	93	72
12	EtOAc‐CH_2_Cl_2_ (1:1)	3	20	72	75^[b]^

[a] Enantiomeric excesses were determined by chiral‐phase HPLC analysis. [b] Synthesis of (*R*)‐**9 a** using catalyst **7 c** (10 mol %).

With the optimised reaction conditions for synthesising both enantiomers of **9 a**, the scope of the substrates was investigated (Scheme 2). The reaction provided satisfactory to excellent yields (31–96 %) under both conditions **A** and **B** for most of the products. Only the thiophene derivative **9 m** is formed in poor yields and 1‐benzosuberone was not reactive under the reaction conditions forming only trace amounts of the product **9 p**. The enantioselectivity was moderate to very good under both reaction conditions ranging from 48–80 % under condition **A** and from 60 % to 80 % under condition **B**, except the α‐tetralone derivative **9 o** was formed in only 32 and 20 % *ee*, respectively. A comparison of the enantioselectivity of the reaction using the conditions **A** and **B** with already reported protocols[^5c^, ^14^] using various classes of chiral iodoarenes/ hypervalent iodine reagents shows a good improvement of the stereochemical induction of the reaction using the newly synthesised C‐N axial chiral iodoarenes **7**, especially **7 d** and **7 c** and demonstrates their potential as organocatalysts in stereoselective oxidative transformations. Propiophenone derivatives with electron‐withdrawing group attached to the aromatic ring (Cl, CF_3_, NO_2_) gave the products **9 b**, **9 c**, **9 d** and **9 e** in very good yields and with good enantioselectivities under both conditions, while the derivatives with electron‐donating groups (Me, OMe, *t*Bu) gave the corresponding products **9 f**, **9 g** and **9 h** in lower yields, but without big influence on the enantioselectivity. Also, the naphthyl derivative **9 i** was obtained in good enantioselectivity but with moderate yield. Changing the aliphatic α‐carbon from methyl to ethyl lead to product **9 j** in excellent yields (>90 %) and good stereoselectivities (77 % and 80 % *ee*) under both conditions **A** and **B**, respectively, while introducing a phenyl group at the aliphatic α‐carbon led to complete loss of stereoselectivity, giving **9 k** in high yields but as a racemate. Heterocyclic ketones containing furan and thiophene moieties provided the corresponding products **9 l** and **9 m** in good (57 %) to high (80 %) enantiomeric excess and high yields for the furan derivative **9 l**. The reactivity and selectivity of the reaction showed dependence on the ring size of cyclic ketones. 1‐Indanone afforded the corresponding product **9 n** in high yields and in moderate *ee*, α‐tetralone gave **9 o** in low conversion and yield and poor *ee* while the seven‐membered ring ketone 1‐benzosuberone was not reactive at all. Changing the sulfonic acid from *p*‐toluenesulfonic acid to benzenesulfonic acid and methanesulfonic acid was successful under both reaction conditions and led to the formation of the propiophenone derivatives **9 q** and **9 r** in excellent yields and with reasonable enantioselectivity.

**Scheme 2:**
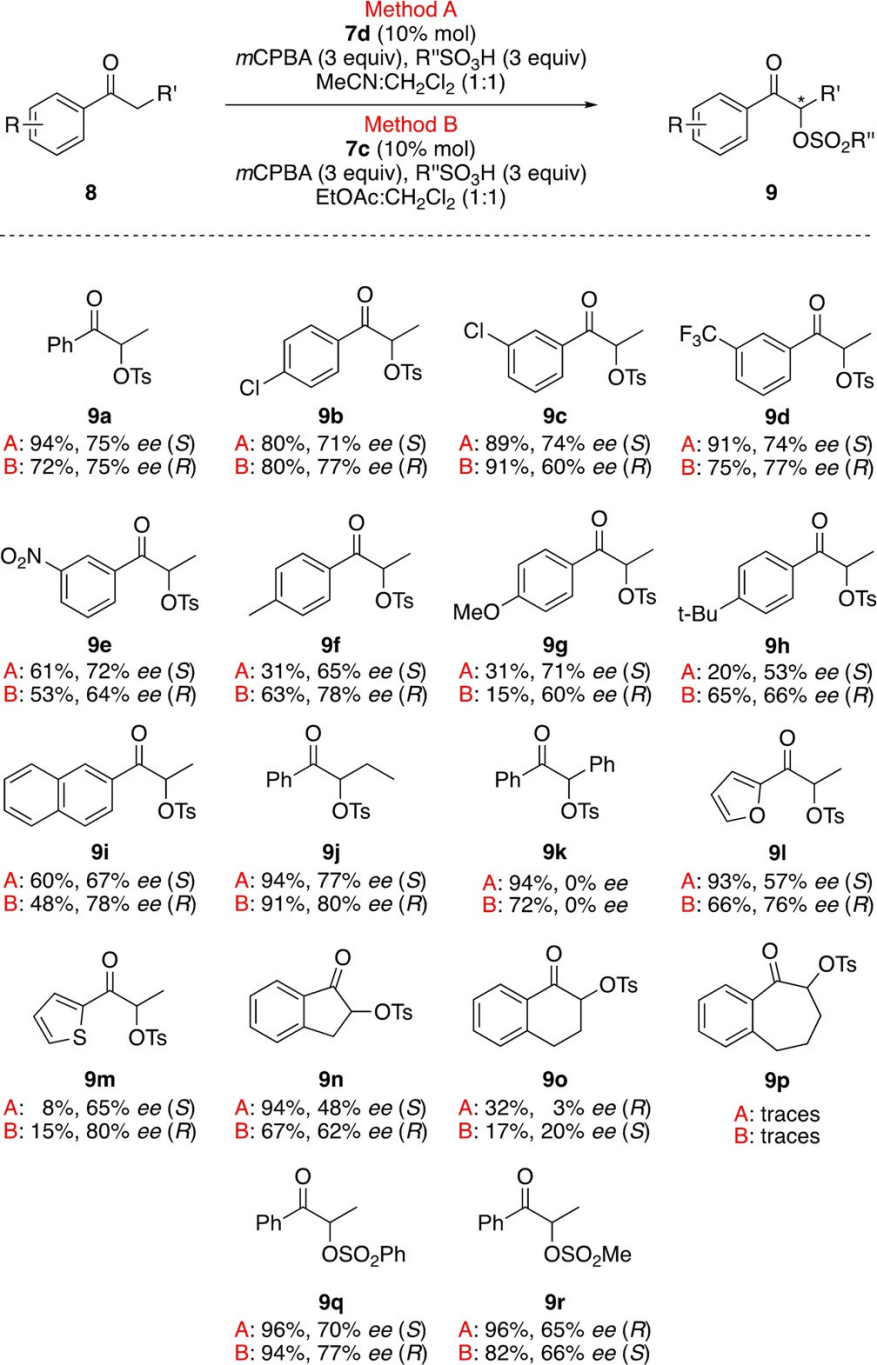
Reaction scope of the stereoselective α‐oxytosylation protocol.

The reaction mechanism was a subject of various studies.[^10a^, ^14c^, ^14d^] The reaction has two possible mechanistic pathways A and B (Scheme 3). Equilibration of **Int‐I** and **Int‐II** could lead to racemisation and account for the lower stereoselectivity of the reaction. Although, Beaulieu and Legault^[14c]^ excluded pathway B and demonstrated computationally that the reaction proceeds via pathway A, the formation of the product from **Int‐I** through a S_N_2’ mechanism could also suffer from low stereoselectivity.

**Scheme 3:**
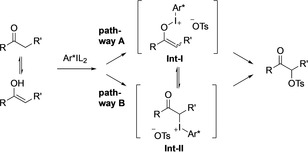
Mechanistic insights of the stereoselective hypervalent iodine mediated α‐oxytosylation of ketones.

In conclusion, ten novel C‐N axial chiral iodoarenes have been synthesised from simple aniline derivatives. Easily separable diastereomers were obtained via chiral resolution using lactate esters, enabling rapid access to the target library of chiral iodoarene reagents avoiding the use of complex and/or expensive reagents and catalysts. Their application in the stereoselective α‐oxytosylation of ketones demonstrated their potential as efficient chiral organocatalysts as a wide range of ketones could be transformed into the corresponding α‐oxygenated products in generally good to excellent yields up to 96 % and with good to high enantioselectivity up to 80 % *ee*. Cyclic and aliphatic ketones remain challenging substrates giving unsatisfactory yields and/or enantioselectivities. The stereochemical reaction is controlled mainly by the axial chirality.

## Experimental Section

Chiral iodine pre‐catalyst **7 c** or **7 d** (0.027 mmol, 0.1 equiv), *m*CPBA (0.81 mmol, 3 equiv), and RSO_3_H (0.81 mmol, 3 equiv) were dissolved in a mixture of MeCN and dichloromethane (1:1) or a mixture of EtOAc and dichloromethane (1:1) followed by the addition of the appropriate ketone **8** (0.27 mmol, 1 equiv). The reaction mixture was stirred at room temperature for 72 h. After completion of the reaction, the mixture was washed with sat. aq. NaHCO_3_ solution and sat. aq. Na_2_S_2_O_3_ solution and extracted with dichloromethane (3×5 mL). The combined organic layers were dried over MgSO_4_, filtered, and concentrated under reduced pressure. The crude products were purified by flash chromatography on silica gel (hexane/EtOAc) to afford pure products **9**.

## Conflict of interest

The authors declare no conflict of interest.

## Supporting information

As a service to our authors and readers, this journal provides supporting information supplied by the authors. Such materials are peer reviewed and may be re‐organized for online delivery, but are not copy‐edited or typeset. Technical support issues arising from supporting information (other than missing files) should be addressed to the authors.

Supplementary
