# Supplementary material for: C−N Axial Chiral Hypervalent Iodine Reagents: Catalytic Stereoselective α‐Oxytosylation of Ketones
Source: Chemistry. 2021 Feb 8;27(13):4317–21. doi: 10.1002/chem.202005253 (PMC7986903; doi:10.1002/chem.202005253)

Chemistry–A European Journal

Supporting Information

C–N Axial Chiral Hypervalent Iodine Reagents: Catalytic Stereoselective α-Oxytosylation of Ketones

Haifa Alharbi,[a] Mohamed Elsherbini,[a, b] Jihan Qurban,[a, c] and Thomas Wirth*[a]

General Methods S1
Procedures and Spectroscopic Data... S3
References S28
NMR spectra S29

General Methods

Chemicals were purchased from Sigma Aldrich, Alfa Aesar, and FluoroChem and were used as received without purification or drying. Dichloromethane was freshly distilled from P_2O_5 under a dried nitrogen atmosphere where dry toluene was collected from a solvent purification system (SPS) from the company M BRAUN (MB SPS-800) stored under a nitrogen atmosphere. Lower temperatures were achieved using ice/water bath (0 °C), dry ice/ acetonitrile bath (-30 °C), dry ice/acetone bath (-78 °C). Thin layer chromatography (TLC) was performed on precoated aluminium sheets of Merck silica gel 60 F254 (0.20 mm) and visualized by UV radiation (254 nm). Automated column chromatography was performed on a Biotage® Isolera Four using Biotage® cartridges SNAP Ultra. ^{1}H NMR and ^{13}C NMR NMR spectra were measured on Bruker DPX 300, 400 or 500 apparatus and were referenced to the residual proton solvent peak (^{1}H: $CDCl_3$, δ 7.26 ppm) and solvent ^{13}C signal ($CDCl_3$, δ 77.2 ppm). Chemical shifts δ were given in ppm and the multiplicity of the signals was reported as: s = singlet, d = doublet, t = triplet, q = quartet, dd = doublet of doublets, dt = doublet of triplet, m = multiplet, b = broad. The coupling constants (J) in Hertz. IR spectra were recorded on a Shimadzu FTIR Affinity-1S apparatus and wave numbers are quoted in cm^{-1}. Mass spectrometric measurements were performed by R. Jenkins, R. Hick, T. Williams, and S. Waller at Cardiff University on a Water LCR Premier XE. Ions were generated by Electrospray (ES) or Electron Ionisation (EI). The molecular ion peak values quoted for molecular ion plus hydrogen $[M+H]^{+}$, molecular ion minus hydrogen $[M-H]^{+}$ or molecular ion plus sodium $[M+Na]^{+}$. Melting points were measured using a

Gallenkamp variable heater with samples in open capillary tubes. The HPLC measurements were performed on a Shimadzu apparatus. The different modules were SIL-10ADVP (auto injector), LC-10ATVP (liquid chromatograph), FCV-10ALVP (pump), DGU-14A (degasser), CTO-10ASVP (column oven), SCL-10AVP (system controller) and SPD-M10A (diode array detector). The solvents used were hexane and 2- propanol as HPLC grade. The chiral column used for the separation of the enantiomers was YMC Chiral Amylose-C S-5μm (0.46 cm Ø x 25 cm). Optical rotation was measured with a SCHMIDT and HAENSCH UniPol L polarimeter at 20 °C in a cuvette of 50 mm length with a sodium light (589.30 nm). X-Ray crystallographic studies were carried out at the X-Ray Crystallography Service at Cardiff University. The data were collected on an Agilent SuperNova Dual Atlas diffractometer with a mirror monochromator, equipped with an Oxford cryosystems cooling apparatus. Crystal structures were solved and refined using SHELX. Nonhydrogen atoms were refined with anisotropic displacement parameters. Hydrogen atoms were inserted in idealised positions. The structure was solved by a direct Method and refined by a full matrix least-squares procedure on F2 for all reflections (SHELXL-97).

Procedures and Spectroscopic Data

Table S1. Solvent screening for catalyst **7c** (reaction conditions B).

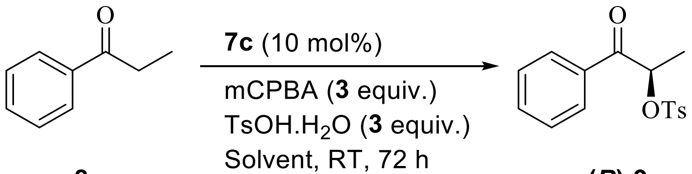

Entry	Solvent	(*R*)-**9a**	
		Yield [%]	ee [%][a]
1	MeCN	93	43
2	CH_2Cl_2	68	54
3	Et_2O	25	59
4	EtOAc	53	69
5	CH_2Cl_2-TFE (1:1)	67	30
6	TFE	63	31
7	HFIP	5	11
8	EtOAc-CH_2Cl_2 (1:1)	72	75
9	EtOAc- Et_2O (1:2)	46	80
10	MeCN-CH_2Cl_2 (1:1)	92	49

[a] Determined by HPLC.

General procedure for the preparation of 2-iodoaniline derivatives (GP 1)

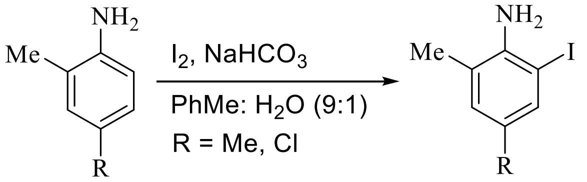

5

Prepared according to a literature procedure.[1] 2-Methyl aniline derivative (40 mmol, 1 equiv.), iodine (40 mmol, 1 equiv.) and sodium bicarbonate (60 mmol, 1.5 equiv.) were dissolved in a mixture of toluene and H_2O (50 mL, 9:1), the mixture was stirred

at room temperature for (5–24) h. The reaction mixture was then diluted with ethyl acetate and washed with aqueous solution of $Na_2S_2O_3$ (2 x 50 mL). The organic layer was separated, and the aqueous layer was extracted with EtOAc (2 x 50 mL). The combined organic layers were washed with brine and dried over $MgSO_4$, filtered, concentrated under reduced pressure. The crude product was purified by flash column chromatography on silica gel (hexane/EtOAc).

2-Iodo-4,6-dimethylaniline (5a)

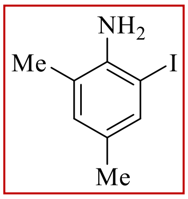

Following **GP 1**: 2,4-dimethyl aniline (4.95 ml, 40 mmol), I_2 (10.2 g, 40 mmol), and $NaHCO_3$ (5.0 g, 60 mmol) to afford **5a** as a light brown solid (8.7 g, 88%). **M.p.:** 65-66 ˚C [Lit[1] 62–64 ºC]. **^{1}H NMR (400 MHz, $CDCl_3$):** δ = 7.36 (s, 1H), 6.84 (s, 1H), 3.93 (bs, 2H), 2.19 (s, 6H) ppm. **^{13}C NMR (101 MHz, $CDCl_3$):** δ = 142.5, 137.0, 131.5, 129.4, 122.6, 84.9, 20.0, 19.0 ppm. The spectroscopic data are in agreement with the literature.[1,2]

4-Chloro-2-iodo-6-methylaniline (5b)

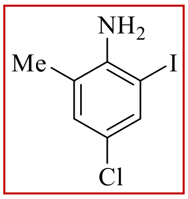

Following **GP 1**: 4-chloro-2-methylaniline (1.0 g, 7 mmol), I_2 (1.77 g, 7 mmol), and $NaHCO_3$ (0.88 g, 10.5 mmol) to yield **5b** as a light yellow solid (0.73 g, 39 %). **M.p.:** 49–50 ºC [Lit[2] 46-47 ºC]. **^{1}H NMR (500 MHz, $CDCl_3$):** δ = 7.49 (d, *J* = 2.2 Hz, 1H), 7.01 (d, *J* = 1.8 Hz, 1H), 4.06 (bs, 2H), 2.20 (s, 3H) ppm. **^{13}C NMR (126 MHz, $CDCl_3$)**: δ = 143.8, 135.7, 130.4, 123.4, 123.0, 83.8, 19.0 ppm. The spectroscopic data are in agreement with the literature. [3]

General procedure for the preparation of *N*-substituted 2-iodoaniline derivatives (GP 2)

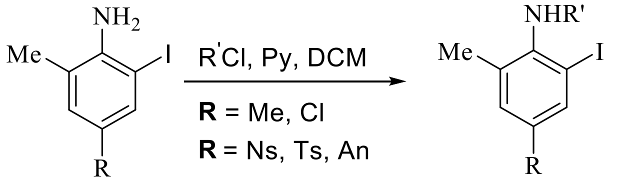

5 **6**

Prepared according literature procedure.[4] To a stirred solution of 2-iodoaniline derivative (12 mmol, 1 equiv.) in dry dichloromethane (25 mL), pyridine (1.45 ml, 18 mmol, 1.5 equiv.) was added the appropriate sulfonyl chloride derivative (12 mmol, 1 equiv.) at 0 °C. The reaction mixture was stirred at ambient temperature for 16 h, then

washed with HCl (1M, 3 x 25 mL). The combined acid wash was extracted with dichloromethane (4 x 25 mL). The combined organic layers were dried over $MgSO_4$, filtered, concentrated under reduced pressure. The crude product was purified by flash column chromatography on silica gel (hexane/EtOAc).

***N*-(2-Iodo-4,6-dimethylphenyl)-4-methylbenzenesulfonamide (6a)**

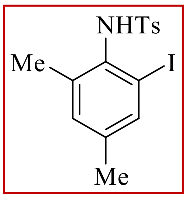

Following **GP 2**: 2-Iodo-4,6-dimethyl aniline **5a** (2.97 g, 12 mmol), 4-methylbenzenesulfonyl chloride (2.29 g, 12 mmol), and pyridine (1.45 ml, 18 mmol) to afford **6a** as a white solid (3.66 g, 76%). **M.p.:** 157–158 ºC [Lit[4] 157–158 ºC]. **^{1}H NMR (400 MHz, $CDCl_3$)**: δ = 7.56 (d, *J* = 8.1 Hz, 2H), 7.38 (s, 1H), 7.24 (d, *J* = 8.1 Hz, 2H), 7.06 (s, 1H), 6.04 (s, 1H), 2.47 (s, 3H), 2.42 (s, 3H), 2.25 (s, 3H) ppm. **^{13}C NMR (126 MHz, $CDCl_3$):** δ = 144.1, 139.6, 139.4, 137.7, 137.4, 133.4, 133.0, 129.7, 128.1, 100.3, 21.8, 20.9, 20.5 ppm. The spectroscopic data are in agreement with the literature.[2]

***N*-(2-Iodo-4,6-dimethylphenyl)-4-nitrobenzenesulfonamide (6b)**

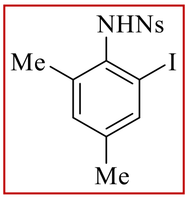

Following **GP 2**, 2-iodo-4,6-dimethyl aniline **5a** (2.97 g, 12 mmol), 4-nitrobenzenesulfonyl chloride (2.66 g, 12 mmol), and pyridine (1.45 ml, 18 mmol) to afford **6b** as a white solid (3.47 g, 67%). **M.p.:** 138–139 ºC. **^{1}H NMR (500 MHz, $CDCl_3$):** δ = 8.30 (d, *J* = 7.9 Hz, 2H), 7.87 (d, *J* = 7.8 Hz, 2H), 7.39 (s, 1H), 7.11 (s, 1H), 6.19 (bs, 1H), 2.52 (s, 3H), 2.26 (s, 3H) ppm. **^{13}C NMR (126 MHz, $CDCl_3$):** δ = 150.6, 145.8, 140.4, 140.2, 137.9, 133.3, 132.3, 129.5, 124.4, 100.1, 21.0, 20.6 ppm. **HRMS** (ESI): $[M-H]^-$ calc. for $[C_{14}H_{12}IN_2O_4S]^-$ 430.9568, found 430.9548. **IR** (neat): ν = 3281, 3105, 1527, 1465, 1334, 1163, 1087, 854, 738, 628 cm^{-1}.

***N*-(2-Iodo-4,6-dimethylphenyl)-4-methoxybenzenesulfonamide (6c)**

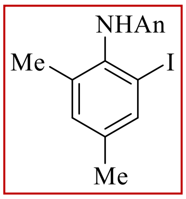

Following **GP 2**, 2-iodo-4,6-dimethyl aniline **5a** (2.97 g, 12 mmol), 4-methoxybenzenesulfonyl chloride (2.48 g, 12 mmol), and pyridine (1.45 ml, 18 mmol) to afford **6c** as a white solid (3.25 g, 65 %). **M.p.:** 176–177 ºC. **^{1}H NMR (500 MHz, $CDCl_3$):** δ = 7.62 – 7.57 (m, 2H), 7.38 (d, *J* = 1.1 Hz, 1H), 7.06 (s, 1H), 6.93 – 6.88 (m, 2H), 6.02 (bs, 1H), 3.87 (s, 3H), 2.49 (s, 3H), 2.25 (s, 3H) ppm. **^{13}C NMR (126 MHz, $CDCl_3$):** δ = 163.6, 139.7, 139.4, 137.7, 133.5, 133.0, 132.1, 130.2, 114.2, 100.2, 55.8, 20.9, 20.5 ppm. **HRMS** (ESI): $[M+H]^+$

calc. for $[C_{15}H_{17}INO_3S]^+$ 417.9968, found 417.9978. **IR** (neat): ν = 3280, 3105, 1527, 1465, 1346, 1334, 1161, 1087, 852, 738, 621 cm^{-1}.

***N*-(4-Chloro-2-iodo-6-methylphenyl)-4-methylbenzenesulfonamide (6d)**

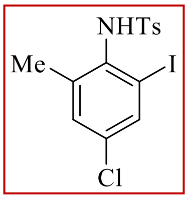

Following **GP 2**, 4-chloro-2-iodo-6-methylaniline **5b** (3.20 g, 12 mmol), 4-methylbenzenesulfonyl chloride (2.29 g, 12 mmol), and pyridine (1.45 ml, 18 mmol) to afford **6d** as a white solid (2.32 g, 46 %). **M.p.**: 101–102 °C. **^{1}H NMR (500 MHz, CDCl$_3$)**: δ = 7.56 (d, *J* = 8.3 Hz, 2H), 7.55 (ddd, *J* = 5.5, 3.0, 2.2 Hz, 1H), 7.26 (d, *J* = 8.0 Hz, 2H), 7.28 – 7.24 (m, 1H), 6.06 (bs, 1H), 2.51 (s, 3H), 2.43 (s, 3H) ppm. **^{13}C NMR (126 MHz, CDCl$_3$):** δ = 144.5, 141.3, 137.1, 136.5, 135.0, 133.9, 132.0, 129.9, 128.1, 100.4, 21.8, 21.1 ppm. **HRMS** (ESI): $[M-H]^+$ calc. 419.9322, found 419.9328 $[C_{14}H_{12}ClINO_2S]^+$. **IR** (neat): ν = 3267, 3010, 2358, 1757, 1456, 1336, 1149, 1087, 857, 732, 588 cm^{-1}.

General procedure for preparation diastereomers of axial chiral hypervalent iodine reagents (GP 3)

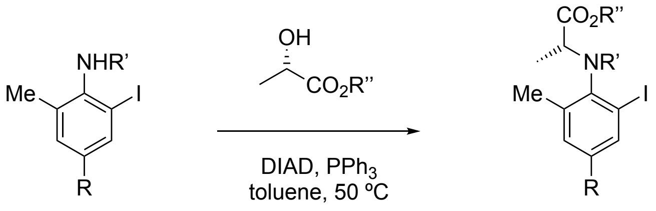

Prepared according to a modified literature procedure.[5] To a stirred solution of *N*-substituted 2-iodoaniline **6** (5.6 mmol, 1 equiv.), triphenylphosphine (8.4 mmol, 1.5 equiv.), and (*S*)-lactate derivative (8.4 mmol, 1.5 equiv.) in dry toluene (30 mL), diisopropyl azodicarboxylate (8.4 mmol, 1.5 equiv.) was added at 0 °C. The reaction mixture was warmed up to room temperature and then stirred at 50 °C for 6 h. The solvent was then removed under reduced pressure and Et_2O was added. The precipitated triphenylphosphine oxide was removed by filtration and the filtrate was concentrated under reduced pressure. The diastereomers were separated by flash column chromatography (SiO_2, *n*-hexane: EtOAc).

(Methyl *N*-(2-iodo-4,6-dimethylphenyl)-*N*-((4-nitrophenyl) sulfonyl)-alaninate

Following **GP 3**, *N*-(2-iodo-4,6-dimethylphenyl)-4 nitrobenzenesulfonamide (**6b**) (2.42 g, 5.6 mmol), PPh_3 (2.20 g, 8.4 mmol), (*S*)-lactic acid methyl ester (0.80 mL, 8.4 mmol),

and DIAD (1.65 mL, 8.4 mmol) to afford two diastereomers (55:45) (2.84 g, 98%); major diastereomer *R*,$S_{N\text{-}C}$: minor diastereomer *R*,$R_{N\text{-}C}$.

(*R,S*$_{N\text{-}C}$)-(Methyl *N*-(2-iodo-4,6-dimethylphenyl)-*N*-((4-nitrophenyl) sulfonyl)-alaninate (7a)

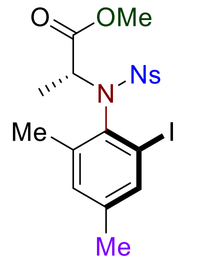
[CCDC-2049029]

White solid. **M.p.** 148–147 °C. **[α]$_D^{20}$** = +10.2 (c = 0.19, $CHCl_3$). **^{1}H NMR (500 MHz, $CDCl_3$):** δ = 8.33 – 8.27 (m, 2H), 7.91 – 7.80 (m, 2H), 7.53 (d, *J* = 1.5 Hz, 1H), 7.05 (s, 1H), 4.90 (q, *J* = 7.2 Hz, 1H), 3.88 (s, 3H), 2.29 (s, 3H), 2.26 (s, 3H), 1.10 (d, *J* = 7.2 Hz, 3H) ppm. **^{13}C NMR (126 MHz, $CDCl_3$):** δ = 173.6, 150.4, 145.8, 144.2, 141.3, 139.2, 134.7, 132.7, 130.3, 123.9, 104.2, 58.3, 52.8, 20.6, 19.9, 17.8 ppm. **HRMS** (ESI): $[M+H]^+$ calc. for $[C_{18}H_{20}IN_2O_6S]^+$ 519.0081, found 519.0084. **IR (neat): ν =** 2949, 1751, 1525, 1456, 1344, 1315, 1205, 1163, 1085, 950, 850, 738, 684, 619, 582, 547 cm^{-1}.

(*R,R*$_{N\text{-}C}$)-(Methyl *N*-(2-iodo-4,6-dimethylphenyl)-*N*-((4-nitrophenyl)sulfonyl)-alaninate (7b)

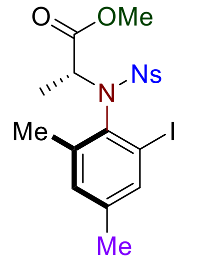
[CCDC-2049032]

White solid. **M.p.** 156–157 °C. **[α]$_D^{20}$** = –25.6 (c = 0.31, $CHCl_3$). **^{1}H NMR (500 MHz, $CDCl_3$):** δ = 8.32 – 8.28 (m, 2H), 7.96 – 7.90 (m, 2H), 7.55-7.51 (m, 1H), 7.12-7.08 (m, 1H), 4.86 (q, *J* = 7.3 Hz, 1H), 3.80 (s, 3H), 2.42 (s, 3H), 2.29 (s, 3H), 1.21 (d, *J* = 7.3 Hz, 3H) ppm. **^{13}C NMR (125 MHz, $CDCl_3$):** δ = 172.0, 150.3,146.3, 143.0, 141.3, 139.6, 135.5, 132.9, 130.8, 103.7, 58.9, 52.6, 21.1, 20.5, 17.3 ppm. **HRMS** (ESI): $[M+H]^+$ calc. for $[C_{18}H_{20}IN_2O_6S]^+$ 519.0081, found 519.0093. **IR** (neat): ν = 2949, 1751, 1525, 1456, 1344, 1315, 1205, 1163, 1085, 950, 850, 738, 684, 619, 582, 547 cm^{-1}.

Methyl *N*-(2-iodo-4,6-dimethylphenyl)-*N*-tosyl-alaninate

Following **GP 3,** *N*-(2-iodo-4,6-dimethylphenyl)-4-methylbenzenesulfonamide **(6a)** (2.25 g, 5.6 mmol), PPh_3 (2.20 g, 8.4 mmol), (*S*)-lactic acid methyl ester (0.80 mL, 8.4 mmol), and DIAD (1.65 mL, 8.4 mmol) to afford two diastereomers (65:35) (1.85 g, 68%); major diastereomer *R*,$S_{N\text{-}C}$: minor diastereomer *R*,$R_{N\text{-}C}$.

(*R,S*$_{N\text{-}C}$)-Methyl *N*-(2-iodo-4,6-dimethylphenyl)-*N*-tosyl-alaninate (7c)

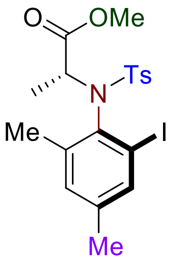

White foam. **[α]$_D$^{20}** = +9.1 (c = 0.22, $CHCl_3$). **^{1}H NMR (500 MHz, $CDCl_3$):** δ = 7.56 (d, *J* = 8.3 Hz, 2H), 7.50 (s, 1H), 7.24 (d, *J* = 8.2 Hz, 2H), 7.01 (s, 1H), 4.88 (q, *J* = 7.1 Hz, 1H), 3.86 (s, 3H), 2.41 (s, 3H), 2.27 (s, 3H), 2.25 (s, 3H), 1.07 (d, *J* = 7.2 Hz, 3H) ppm. **^{13}C NMR (125 MHz, $CDCl_3$):** δ = 173.8, 144.6, 143.7, 140.4, 138.8, 137.4, 135.4, 132.2, 129.3, 128.8, 104.1, 57.6, 52.5, 21.6, 20.4, 19.9, 17.8 ppm. **HRMS** (ESI): $[M+H]^+$ calc. for $[C_{19}H_{23}INO_4S]^+$ 488.0387, found 488.0383. **IR** (neat): ν = 2951, 1751, 1597, 1452, 1344, 1161, 1085, 916, 592 cm^{-1}.

(*R, R*$_{N\text{-}C}$)-Methyl *N*-(2-iodo-4,6-dimethylphenyl)-*N*-tosyl-alaninate (7d)

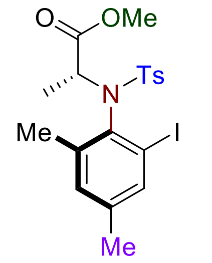

[CCDC-2049034]

White solid. **m.p.**: 102–103 °C. **[α]$_D$^{20}** = –6.0 (c = 1.0, $CHCl_3$).**^{1}H NMR (500 MHz, $CDCl_3$):** δ = 7.64 (d, *J* = 8.3 Hz, 2H), 7.56 (s, 1H), 7.26 (d, *J* = 8.3 Hz, 2H), 7.02 (s, 1H), 4.74 (q, *J* = 7.2 Hz, 1H), 3.77 (s, 3H), 2.42 (s, 3H), 2.26 (s, 3H), 2.23 (s, 3H), 1.19 (d, *J* = 7.2 Hz, 3H) ppm. **^{13}C NMR (126 MHz, $CDCl_3$):** δ = 172.4, 143.9, 142.3, 140.5, 139.4, 137.8, 136.2, 132.6, 129.3, 129.2, 105.0, 58.5, 52.3, 21.8, 20.8, 20.5, 17.5 ppm. **HRMS** (ESI): $[M+H]^+$ for $[C_{19}H_{23}INO_4S]^+$ 488.0387, found 488.0393 $[C_{19}H_{23}INO_4S]^+$. **IR (neat): ν =** 2949, 1759, 1597, 1456, 1338, 1161, 1085, 916, 592 cm^{-1}.

Methyl*N*-(2-iodo-4,6-dimethylphenyl)-*N*-((4-methoxyphenyl)sulfonyl)-D-alaninate

Following **GP 3,** *N*-(2-iodo-4,6-dimethylphenyl)-4-methoxybenzenesulfonamide **(6c)** (2.34 g, 5.6 mmol), PPh_3 (2.20 g, 8.4 mmol), (*S*)-lactic acid methyl ester (0.80 mL, 8.4 mmol), and DIAD (1.65 mL, 8.4 mmol) to afford two diastereoisomers (65:35) (1.60 g, 57%); major diastereomer *R*,*S*$_{N\text{-}C}$: minor diastereomer *R*,*R*$_{N\text{-}C}$.

(*R,S*$_{N\text{-}C}$)-Methyl N-(2-iodo-4,6-dimethylphenyl)-N-((4-methoxyphenyl) sulfonyl)-D-alaninate (7e)

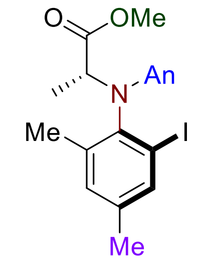

[CCDC-2049030]

White solid. **M.p.**: 124–125 °C. **[α]$_D$ ^{20}** = +3.3 (c = 1.8, $CHCl_3$). **^{1}H NMR (500 MHz, $CDCl_3$):** δ = 7.63 – 7.58 (m, 2H), 7.52 – 7.48 (m, 1H), 7.02 – 7.00 (m, 1H), 6.93 – 6.89 (m, 2H), 4.87 (q, *J* = 7.2 Hz, 1H), 3.86 (s, 3H), 3.86 (s, 3H), 2.28 (s, 3H), 2.26 (s, 3H), 1.07 (d, *J* = 7.2 Hz, 3H) ppm. **^{13}C NMR (126 MHz, $CDCl_3$):** δ = 174.1, 163.4, 144.8, 140.6, 138.9, 135.5, 132.4, 131.0, 113.9, 104.1, 57.7, 55.7, 52.6, 20.6, 19.9, 17.9 ppm. **HRMS** (ESI): $[M+H]^+$ calc. for $[C_{19}H_{22}INO_5S]^+$ 504.0336, found 504.0347. **IR (neat): v =** 2360, 2341, 1743, 1458, 1344, 1257, 1195, 1157, 680, 592, 545 cm^{-1}.

(*R,R$_{N-C}$*)-Methyl N-(2-iodo-4,6-dimethylphenyl)-N-((4-methoxyphenyl) sulfonyl)-D-alaninate (7f)

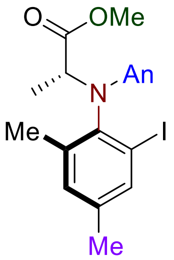

White solid. **m.p.**: 119–120 °C. **[α]$_D$ ^{20}** = –1.7 (c = 2.4, $CHCl_3$). **^{1}H NMR (500 MHz, $CDCl_3$):** δ = 7.70 – 7.66 (m, 2H), 7.56 – 7.54 (m, 1H), 7.03 – 7.01 (m, 1H), 6.94 – 6.91 (m, 2H), 4.74 (q, *J* = 7.2 Hz, 1H), 3.86 (s, 3H), 3.78 (s, 3H), 2.26 (s, 6H), 1.17 (d, *J* = 7.2 Hz, 3H) ppm. **^{13}C NMR (126 MHz, $CDCl_3$):** δ = 172.5, 163.4, 142.3, 140.5, 139.4, 136.1, 132.6, 132.5, 131.3, 113.8, 105.0, 58.4, 55.7, 52.3, 20.8, 20.5, 17.5 ppm. **HRMS** (ESI): $[M+H]^+$ calc. for $[C_{19}H_{22}INO_5S]^+$ 504.0336, found 504.0342 $[C_{19}H_{22}INO_5S]^+$. **IR (neat): v =** 2360, 2341, 1743, 1593, 1346, 1257, 1157, 682, 592, 543 cm^{-1}.

(*R*)-Methyl N-(4-chloro-2-iodo-6-methylphenyl)-N-tosyl-L-alaninate

Following **GP 3,** *N*-(4-chloro-2-iodo-6-methylphenyl)-4-methylbenzenesulfonamide **(6d)** (2.0 g, 4.7 mmol), PPh_3 (1.84 g, 7 mmol), (*S*)-lactic acid methyl ester (0.67 mL, 7 mmol), and DIAD (1.38 mL, 7 mmol) to afford two diastereomers (65:35) (2.22 g, 93%); major diastereomer *R*,*S*$_{N-C}$: minor diastereomer *R*,*R*$_{N-C}$.

(*R,S$_{N-C}$*)-Methyl *N*-(4-chloro-2-iodo-6-methylphenyl)-*N*-tosyl-L-alaninate (7g)

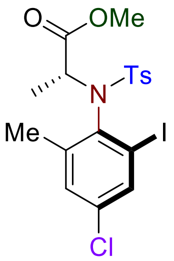

White foam. **[α]$_D$ ^{20}** = +2.2 (c = 2.7, $CHCl_3$). **^{1}H NMR (500 MHz, $CDCl_3$):** δ = 7.66 (dd, *J* = 2.5, 0.6 Hz, 1H), 7.58 – 7.54 (m, 2H), 7.27 – 7.24 (m, 2H), 7.21 (dd, *J* = 2.5, 0.6 Hz, 1H), 4.87 (q, *J* = 7.2 Hz, 1H), 3.86 (s, 3H), 2.42 (s, 3H), 2.30 (t, *J* = 0.5 Hz, 3H), 1.08 (d, *J* = 7.2 Hz, 3H) ppm. **^{13}C NMR (126 MHz, $CDCl_3$):** δ = 173.7, 146.5, 144.2, 137.6, 137.24,

137.18, 135.1, 131.4, 129.6, 128.9, 104.5, 57.7, 52.7, 21.8, 20.1, 17.9 ppm. **HRMS** (ESI): $[M+Na]^+$ calc. for $[C_{18}H_{19}ClINO_4SNa]^+$ 529.9660, found 529.9671. **IR (neat): v =** 2358, 1749, 1456, 1346, 1155, 1083, 669, 586, 540 cm^{-1}.

(*R,R*$_{N\text{-}C}$)-Methyl *N*-(4-chloro-2-iodo-6-methylphenyl)-*N*-tosyl-L-alaninate (7h)

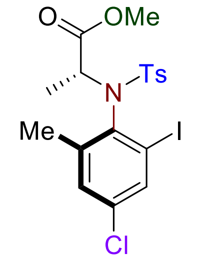

[CCDC-2049031]

White solid. **M.p.**: 117–118 °C. **[α]$_D$ ^{20}** = –3.8 (c = 3.2, $CHCl_3$). **^{1}H NMR (500 MHz, $CDCl_3$):** δ = 7.72 (dd, *J* = 2.5, 0.6 Hz, 1H), 7.65 – 7.61 (m, 2H), 7.30 – 7.26 (m, 2H), 7.23 (dt, *J* = 3.7, 1.8 Hz, 1H), 4.74 (q, *J* = 7.2 Hz, 1H), 3.78 (s, 3H), 2.43 (s, 3H), 2.28 (s, 3H), 1.20 (d, *J* = 7.2 Hz, 3H) ppm. **^{13}C NMR (126 MHz, $CDCl_3$):** δ = 172.1, 144.2, 144.1, 138.1, 137.8, 137.5, 135.1, 131.6, 129.4, 129.2, 105.4, 58.5, 52.4, 21.8, 20.9, 17.5 ppm. **HRMS** (ESI): $[M+Na]^+$ calc. for $[C_{18}H_{19}ClINO_4SNa]^+$ 529.9660, found 529.9667. **IR (neat): v =** 2360, 1757, 1456, 1338, 1153, 1085, 673, 588, 538 cm^{-1}.

Benzyl *N*-(2-iodo-4,6-dimethylphenyl)-N-((4-nitrophenyl)sulfonyl)-D-alaninate

Following **GP 3,** *N*-(2-iodo-4,6-dimethylphenyl)-4 nitrobenzenesulfonamide **(6b)** (2.42 g, 5.6 mmol), PPh_3 (2.20 g, 8.4 mmol), (*S*)-lactic acid benzyl ester (1.35 mL, 8.4 mmol), and DIAD (1.65 mL, 8.4 mmol) to afford two diastereomers (60:40) (2.32 g, 68%); major diastereomer *R*,*S*$_{N\text{-}C}$: minor diastereomer *R*,*R*$_{N\text{-}C}$.

(*R,S*$_{N\text{-}C}$)-(Benzyl *N*-(2-iodo-4,6-dimethylphenyl)-*N*-((4-nitrophenyl) sulfonyl)-alaninate (7i)

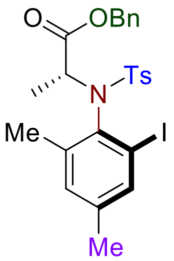

Colourless oil. **[α]$_D$ ^{20}** = +20 (c = 0.20, $CHCl_3$). **^{1}H NMR (500 MHz, $CDCl_3$):** δ = 8.26 – 8.20 (m, 2H), 7.78 – 7.73 (m, 2H), 7.53 (dd, *J* = 1.4, 0.6 Hz, 1H), 7.49 – 7.45 (m, 2H), 7.44 – 7.40 (m, 2H), 7.40 – 7.36 (m, 1H), 7.02 (dd, *J* = 1.4, 0.7 Hz, 1H), 5.32 (d, *J* = 12.1 Hz, 1H), 5.26 (d, *J* = 12.1 Hz, 1H), 4.96 (q, *J* = 7.2 Hz, 1H), 2.28 (s, 3H), 2.19 (s, 3H), 1.09 (d, *J* = 7.2 Hz, 3H) ppm. **^{13}C NMR (126 MHz, $CDCl_3$)**: δ = 172.9, 150.3, 145.8, 144.0, 141.3, 139.2, 135.6, 134.7, 132.6, 130.2, 128.8, 128.71, 128.67, 123.9, 104.4, 67.8, 58.4, 20.6, 20.0, 17.8 ppm. **HRMS** (ESI): $[M+H]^+$ calc. for $[C_{24}H_{24}IN_2O_6S]^+$ 595.0394, found 595.0419. **IR (neat): v =** 2360, 1747, 1529, 1348, 1166, 854, 738, 617 cm^{-1}.

(*R,R*$_{N\text{-}C}$)-(Benzyl *N*-(2-iodo-4,6-dimethylphenyl)-*N*-((4-nitrophenyl) sulfonyl)-alaninate (7j)

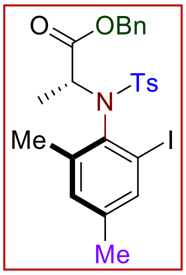

White foam. **[α]$_D$ ^{20}** = –25 (c = 0.16, $CHCl_3$). **^{1}H NMR (500 MHz, $CDCl_3$):** δ = 8.24 – 8.18 (m, 2H), 7.86 – 7.81 (m, 2H), 7.52 (dd, *J* = 1.4, 0.6 Hz, 1H), 7.43 – 7.34 (m, 5H), 7.09 (dd, *J* = 1.4, 0.7 Hz, 1H), 5.27 (d, *J* = 12.2 Hz, 1H), 5.21 (d, *J* = 12.2 Hz, 1H), 4.91 (q, *J* = 7.3 Hz, 1H), 2.42 (s, 3H), 2.28 (s, 3H), 1.21 (d, *J* = 7.3 Hz, 3H) ppm. **^{13}C NMR (126 MHz, $CDCl_3$):** δ = 171.4, 150.3, 146.2, 143.0, 141.3, 139.6, 135.50, 135.45, 132.9, 130.8, 128.8, 128.7, 128.6, 123.7, 103.8, 67.5, 59.1, 21.1, 20.5, 17.4 ppm. **HRMS** (ESI): $[M+H]^+$ calc. for $[C_{24}H_{24}IN_2O_6S]^+$ 595.0394, found 595.0402 $[C_{24}H_{24}IN_2O_6S]^+$. **IR (neat): ν =** 2360, 1751, 1529, 1348, 1166, 740 cm^{-1}.

General procedure for of catalytic enantioselective *α*-tosyloxylation of ketones (GP 5)

Method A:

Chiral iodine catalyst **7d** (0.027 mmol, 0.1 equiv.), *m*CPBA (0.81 mmol, 3 equiv.), and RSO_3H (0.81 mmol, 3 equiv.) were dissolved in a mixture of MeCN and dichloromethane (1:1), followed by the addition of the appropriate ketone (0.27 mmol, 1 equiv.). The reaction mixture was stirred at room temperature for 72 h. After completion of the reaction, the mixture was washed with sat. aq. $NaHCO_3$ solution and sat. aq. $Na_2S_2O_3$ solution and extracted with dichloromethane (3 x). The combined organic layers were dried over $MgSO_4$, filtered, concentrated under reduced pressure. The crude products were purified by flash chromatography on silica gel (hexane/EtOAc) to afford the desired pure products.

Method B:

Chiral iodine catalyst **7c** (0.027 mmol, 0.1 equiv.), *m*CPBA (0.81 mmol, 3 equiv.), and RSO_3H (0.81 mmol, 3 equiv.) were dissolved in a mixture of EtOAc and dichloromethane (1:1) followed by the addition of the appropriate ketone (0.27 mmol, 1 equiv.). The reaction mixture was stirred at room temperature for 72 h. After completion of the reaction, the mixture was washed with sat. aq. $NaHCO_3$ solution and sat. aq. $Na_2S_2O_3$ solution and extracted with dichloromethane (3 x). The combined organic layers were dried over $MgSO_4$, filtered, concentrated under reduced pressure. The crude products were purified by flash chromatography on silica gel (hexane/EtOAc) to afford the desired pure products.

1-Oxo-1-phenylpropan-2-yl 4-methylbenzenesulfonate (9a)

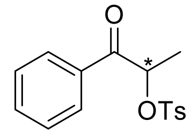

[CCDC-2049033]

Following **GP 5** Method **A** and Method **B**: Chiral iodine catalyst (13 mg, 0.027 mmol), *m*CPBA (184 mg, 0.81 mmol), *p*-TsOH hydrate (154 mg, 0.81 mmol), and propiophenone (**8a)** (36.4 mg, 0.27 mmol) to afford **9a** as a white solid. **M.p.**: 64–65 °C. **^{1}H NMR (400 MHz, $CDCl_3$):** δ = 7.89–7.86 (m, 2H), 7.77–7.73 m, 2H), 7.59 (ddt, *J* = 7.9, 7.0, 1.3 Hz, 1H), 7.48-7.43 (m, 2H), 7.28–7.24 (m, 2H), 5.78 (q, *J* = 6.9 Hz, 1H), 2.41 (s, 3H), 1.6 (d, *J* = 6.9 Hz, 3H) ppm. **^{13}C NMR (101 MHz, $CDCl_3$):** δ = 195.0, 145.2, 134.0, 133.9, 133.7, 129.9, 128.9, 128.1, 77.5, 21.8, 18.9 ppm. The spectroscopic data are in agreement with the literature.[6]

(*S*)-9a: Prepared by Method **A**: white solid (78 mg, 95%), **$[\alpha]_D^{20}$** = –12.0 (c = 2.0, $CHCl_3$). Enantiomeric excess is determined by HPLC YMC Chiral Amylose-C S-5 µm (25 cm), (*n*-hexane/*i*-PrOH = 80/20, flow rate = 1.0 mL/min, 254 nm): major isomer: t_R = 9.84 min, minor isomer: t_R = 8.96 min, *ee* = 75%.

(*R*)-9a: Prepared by Method **B:** white solid (59 mg, 72%), **$[\alpha]_D^{20}$** = +8.33 (c = 1.2, $CHCl_3$). Enantiomeric excess is determined by HPLC YMC Chiral Amylose-C S-5 µm (25 cm), (*n*-hexane/*i*-PrOH = 80/20, flow rate = 1.0 mL/min, 254 nm): major isomer: t_R = 8.97 min, minor isomer: t_R = 9.86 min, ee = 75%.

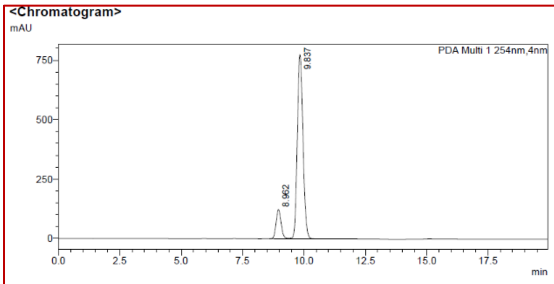

Peak Table

PDA Ch1 254nm

Peak#	Ret. Time	Area%
1	8.962	12.315
2	9.837	87.685
Total		100.000

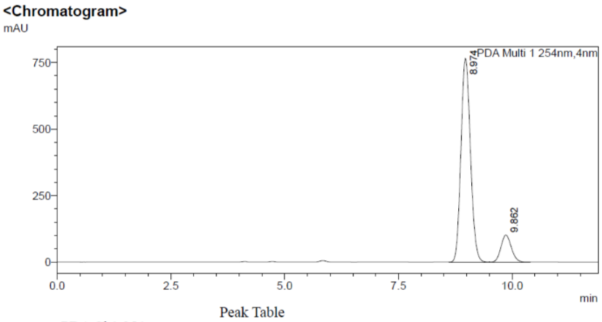

Peak Table

PDA Ch1 254nm

Peak#	Ret. Time	Area%
1	8.974	87.327
2	9.862	12.673
Total		100.000

1-(4-Chlorophenyl)-1-oxopropan-2yl 4-methylbenzenesulfonate (9b)

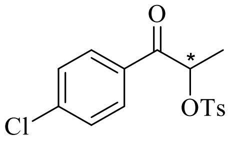

Following **GP 5** Method **A** and Method **B**: Chiral iodine catalyst (13 mg, 0.027 mmol), *m*CPBA (184 mg, 0.81 mmol), *p*-TsOH hydrate (154 mg, 0.81 mmol), and 4-chloropropiophenone (**8b)** (46 mg, 0.27 mmol) to afford **9b** as a white solid. **M.p.**:106–108 °C. **^{1}H NMR (400 MHz, $CDCl_3$)**: δ =

7.87 – 7.81 (m, 2H), 7.74 (d, *J* = 8.3 Hz, 2H), 7.46 – 7.40 (m, 2H), 7.31 – 7.26 (m, 2H), 5.68 (q, *J* = 6.9 Hz, 1H), 2.42 (s, 3H), 1.58 (d, *J* = 6.9 Hz, 3H) ppm. **^{13}C NMR (101 MHz, $CDCl_3$)**: δ = 194.0, 145.3, 140.6, 133.5, 132.2, 130.4, 129.9, 129.3, 128.1, 77.4, 21.8, 18.7 ppm. The spectroscopic data are in agreement with the literature.[7]

(*S*)-9b: Prepared by Method **A:** white solid (73 mg, 80%), **$[\alpha]_D^{20}$** = –1.1 (c = 1.76, $CHCl_3$). Enantiomeric excess is determined by HPLC YMC Chiral Amylose-C S-5 µm (25 cm), (*n*-hexane/*i*-PrOH = 80/20, flow rate = 1.0 mL/min, 254 nm): major isomer: t_R = 13.49 min, minor isomer: t_R = 10.01 min, *ee* = 71%.

(*R*)-9b: Prepared by Method **B:** white solid (73 mg, 80%), **$[\alpha]_D^{20}$** = +11.1 (c=0.36, $CHCl_3$). Enantiomeric excess is determined by HPLC YMC Chiral Amylose-C S-5 µm (25 cm), (*n*-hexane/*i*-PrOH = 80/20, flow rate = 1.0 mL/min, 254 nm): major isomer: t_R = 10.00 min, minor isomer: t_R = 13.52 min, *ee* = 77%.

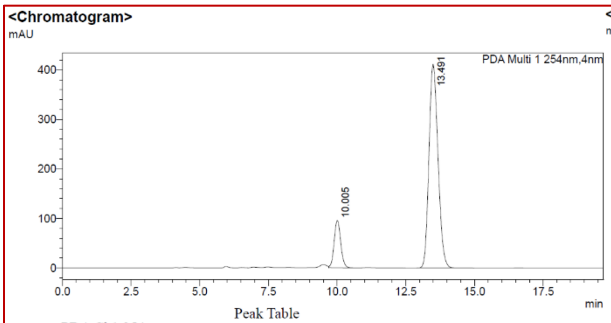

Peak Table

PDA Ch1 254nm

Peak#	Ret. Time	Area%
1	10.005	14.414
2	13.491	85.586
Total		100.000

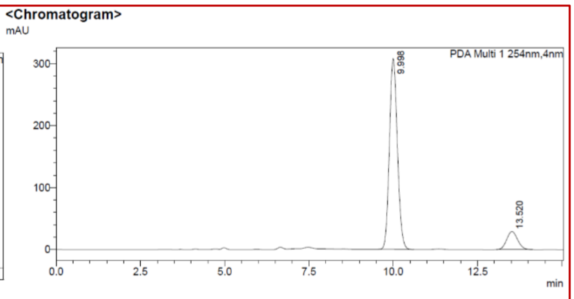

Peak Table

PDA Ch1 254nm

Peak#	Ret. Time	Area%
1	9.998	88.466
2	13.520	11.534
Total		100.000

1-(3-Chlorophenyl)-1-oxopropan-2-yl 4-methylbenzenesulfonate (9c)

Following **GP 5** Method **A** or Method **B**, chiral iodine catalyst (13 mg, 0.027 mmol), *m*CPBA (184 mg, 0.81 mmol), *p*-TsOH hydrate (154 mg, 0.81 mmol), and 3-chloropropiophenone (**8c)** (46 mg, 0.27 mmol) to afford **9c** as a white solid. **M.p.**: 145–147 °C. **^{1}H NMR (500 MHz, $CDCl_3$):** δ = 7.82 – 7.76 (m, 2H), 7.75 – 7.70 (m, 2H), 7.41 – 7.38 (m, 1H), 7.29 – 7.26 (m, 2H), 5.69 (q, *J* = 7.0 Hz, 1H), 2.42 (s, 3H), 1.60 (d, *J* = 6.9 Hz, 3H). ppm. **^{13}C NMR (126 MHz, $CDCl_3$):** δ = 194.1, 169.7, 145.4, 135.4, 135.3, 133.9, 133.4, 130.2, 130.0, 128.9, 128.1, 127.0, 77.6, 21.8, 18.8 ppm. The spectroscopic data are in agreement with the literature.[9]

(S)-9c: Prepared by Method **A:** white solid (81 mg, 89%), **$[\alpha]_D^{20}$** = –15.4 (c = 0.13 , $CHCl_3$). Enantiomeric excess is determined by HPLC YMC Chiral Amylose-C S-5µm

(25 cm), (*n*-hexane/*i*-PrOH = 95/5, flow rate = 1.0 mL/min, 254 nm): major isomer: t_R = 9.2 min, minor isomer: t_R = 8.2 min, *ee* = 74%.

(R)-9c: Prepared by Method **B:** white solid (83 mg, 91%), $[\alpha]_D^{20}$ =+20 (c=0.1, $CHCl_3$). Enantiomeric excess is determined by HPLC YMC Chiral Amylose-C S-5µm (25 cm), (*n*-hexane/*i*-PrOH = 95/5, flow rate = 1.0 mL/min, 254 nm): major isomer: t_R = 8.2 min, minor isomer: t_R = 9.2 min, *ee* = 60%.

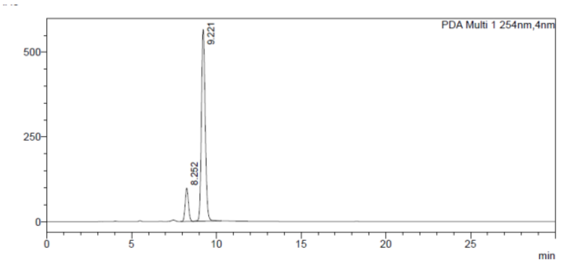

Peak Table

PDA Ch1 254nm

Peak#	Ret. Time	Area%
1	8.252	12.905
2	9.221	87.095
Total		100.000

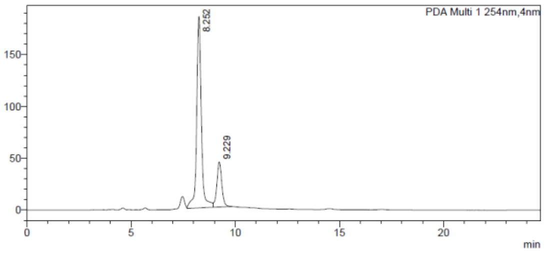

Peak Table

PDA Ch1 254nm

Peak#	Ret. Time	Area%
1	8.252	79.968
2	9.229	20.032
Total		100.000

1-Oxo-1-(3-(trifluoromethyl) phenyl) propan-2-yl 4-methylbenzenesulfonate (9d)

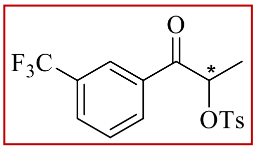

Following **GP 5** Method **A** and Method **B**, chiral iodine catalyst (13 mg, 0.027 mmol), *m*CPBA (184 mg, 0.81 mmol), *p*-TsOH hydrate (154 mg, 0.81 mmol), and 3′-(trifluoromethyl)propiophenone (**8d)** (55 mg, 0.27 mmol) to afford **9d** as a white solid. **M.p.**: 100–101 °C. **^{1}H NMR (400 MHz, $CDCl_3$):** δ = 8.10 (d, *J* = 8.1 Hz, 2H), 7.83 (d, *J* = 7.9 Hz, 1H), 7.74 – 7.69 (m, 2H), 7.61 (t, *J* = 7.7 Hz, 1H), 7.29 – 7.22 (m, 2H), 5.70 (q, *J* = 6.9 Hz, 1H), 2.41 (s, 3H), 1.61 (d, *J* = 6.9 Hz, 3H) ppm. **^{13}C NMR (101 MHz, $CDCl_3$):** δ = 194.2, 145.5, 134.4, 133.3, 132.1, 130.3, 130.0, 129.5, 128.1, 125.8, 77.7, 21.8, 18.7 ppm. The spectroscopic data are in agreement with the literature.[8]

(*S*)-9d: Prepared by Method **A:** white solid (91 mg, 91%), $[\alpha]_D^{20}$ = –1.2 (c = 5.0, $CHCl_3$). Enantiomeric excess is determined by HPLC YMC Chiral Amylose-C S-5µm (25 cm), (*n*-hexane/*i*-PrOH = 80/20, flow rate = 1.0 mL/min, 254 nm): major isomer: t_R = 7.07 min, minor isomer: t_R = 6.38 min, ee= 74%.

(*R*)-9d: Prepared by Method **B:** white solid (95 mg, 95%), $[\alpha]_D^{20}$ = +6.7 (c = 0.3, $CHCl_3$). Enantiomeric excess is determined by HPLC YMC Chiral Amylose-C S-5µm (25 cm), (*n*-hexane/*i*-PrOH = 80/20, flow rate = 1.0 mL/min, 254 nm): major isomer: t_R = 6.40 min, minor isomer: t_R = 7.11min, ee= 77%.

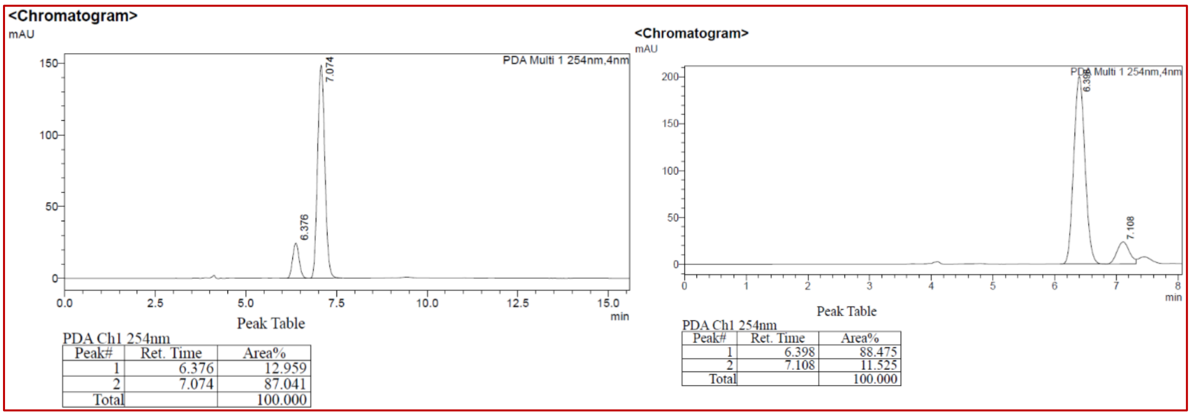

1-(3-Nitrophenyl)-1-oxopropan-2-yl 4-methylbenzenesulfonate (9e)

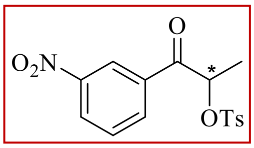

Following **GP 5** Method **A** or Method **B**, chiral iodine catalyst (13 mg, 0.027 mmol), *m*CPBA (184 mg, 0.81 mmol), *p*-TsOH hydrate (154 mg, 0.81 mmol), and 3-nitropropiophenone (**8e**) (48 mg, 0.27 mmol) to afford **9e** as a white solid. **M.p.**: 80–82 °C. **^{1}H NMR (400 MHz, $CDCl_3$):** δ = 8.67 (dd, *J* = 2.9, 1.1 Hz, 1H), 8.43 (ddd, *J* = 8.2, 2.3, 1.1 Hz, 1H), 8.27 (ddd, *J* = 7.8, 1.7, 1.1 Hz, 1H), 7.77 – 7.65 (m, 3H), 7.32 – 7.26 (m, 2H), 5.68 (q, *J* = 7.0 Hz, 1H), 2.42 (s, 3H), 1.63 (d, *J* = 7.0 Hz, 3H) ppm. **^{13}C NMR (101 MHz, $CDCl_3$):** δ = 193.6, 148.5, 145.7, 135.2, 134.5, 133.2, 130.2, 130.1, 128.1, 128.0, 123.9, 77.8, 21.8, 18.5 ppm. The spectroscopic data are in agreement with the literature.[8]

(S)-9e: Prepared by Method **A:** white solid (57 mg, 61%), **$[\alpha]_D^{20}$** =-7.4 (c=0.54, $CHCl_3$). Enantiomeric excess is determined by HPLC YMC Chiral Amylose-C S-5μm (25 cm), (*n*-hexane/*i*-PrOH = 95/5, flow rate = 1.0 mL/min, 254 nm): major isomer: t_R = 19.9 min, minor isomer: t_R = 16.5 min, *ee* = 72%.

(R)-9e: Prepared by Method **B:** white solid (50 mg, 53 %), **$[\alpha]_D^{20}$** =+11.1 (c=0.18, $CHCl_3$). Enantiomeric excess is determined by HPLC YMC Chiral Amylose-C S-5μm (25 cm), (*n*-hexane/*i*-PrOH = 95/5, flow rate = 1.0 mL/min, 254 nm): major isomer: t_R = 16.5 min, minor isomer: t_R = 19.9 min, *ee* = 64%.

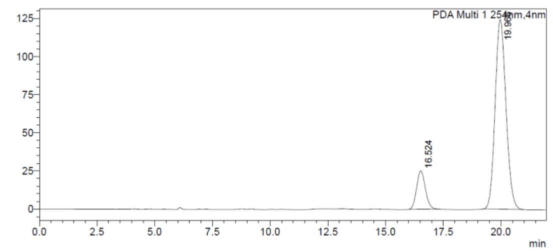

Peak Table

PDA Ch1 254nm

Peak#	Ret. Time	Area%
1	16.524	14.087
2	19.966	85.913
Total		100.000

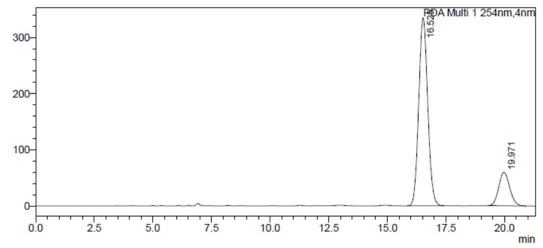

Peak Table

PDA Ch1 254nm

Peak#	Ret. Time	Area%
1	16.525	82.178
2	19.971	17.822
Total		100.000

1-Oxo-1-(p-tolyl) propan-2-yl 4-methylbenzenesulfonate (9f)

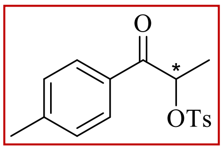

Following **GP 5** Method **A** and Method **B**: Chiral iodine catalyst (13 mg, 0.027 mmol), *m*CPBA (184 mg, 0.81 mmol), *p*-TsOH hydrate (154 mg, 0.81 mmol), and 4-methylpropiophenone (**8f)** (40 mg, 0.27 mmol) to afford **9f** as a white solid. **M.p.**: 85–86 °C. **^{1}H NMR (400 MHz, $CDCl_3$):** δ = 7.81-7.73 (m, 4H), 7.29 – 7.22 (m, 4H), 5.77 (q, *J* = 6.9 Hz, 1H), 2.41 (s, 3H), 2.40 (s, 3H), 1.58 (d, *J* = 6.9 Hz, 3H) ppm. **^{13}C NMR (101 MHz, $CDCl_3$):** δ = 194.5, 145.1 (2 peaks), 133.7, 131.3, 129.9, 129.6, 129.0, 128.1, 77.5, 21.9, 21.8, 19.0 ppm. The spectroscopic data are in agreement with the literature. [8]

(*S*)-9f: Prepared by Method **A:** white solid (27 mg, 31%), **$[\alpha]_D$ ^{20}** = -7.69 (c=0.26, $CHCl_3$). Enantiomeric excess is determined by HPLC YMC Chiral Amylose-C S-5μm (25 cm), (*n*-hexane/*i*-PrOH = 80/20, flow rate = 1.0 mL/min, 254 nm): major isomer: t_R = 11.81 min, minor isomer: t_R = 10.10 min, *ee* = 69%.

(*R*)-9f: Prepared by Method **B:** white solid (54 mg, 63%), **$[\alpha]_D$ ^{20}** = + 2.0 (c=1.0, CHCl3). Enantiomeric excess is determined by HPLC YMC Chiral Amylose-C S-5μm (25 cm), (*n*-hexane/*i*-PrOH = 80/20, flow rate = 1.0 mL/min, 254 nm): major isomer: t_R = 10.07 min, minor isomer: t_R = 11.83 min, *ee* = 78%.

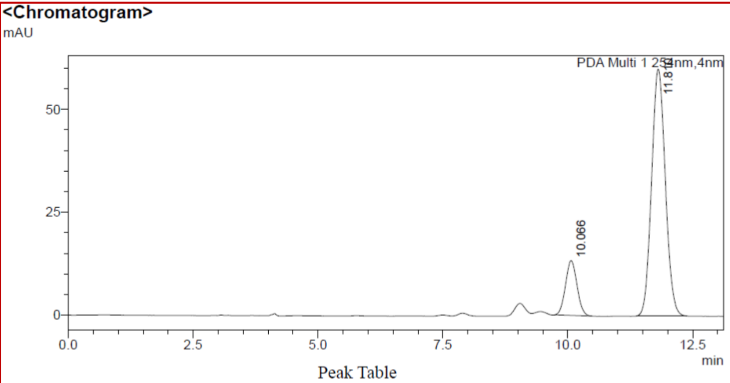

Peak Table

PDA Ch1 254nm

Peak#	Ret. Time	Area%
1	10.066	15.677
2	11.810	84.323
Total		100.000

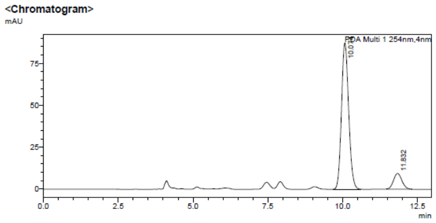

Peak Table

PDA Ch1 254nm

Peak#	Ret. Time	Area%
1	10.074	88.895
2	11.832	11.105
Total		100.000

1-(4-Methoxyphenyl)-1-oxopropan-2-yl 4-methylbenzenesulfonate (9g)

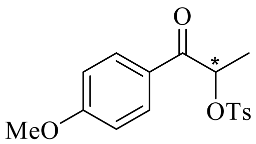

Following **GP 5** Method **A** and Method **B**: Chiral iodine catalyst (13 mg, 0.027 mmol), *m*CPBA (184 mg, 0.81 mmol), *p*-TsOH hydrate (154 mg, 0.81 mmol), and 4-methoxypropiophenone (**8g)** (44 mg, 0.27 mmol) to afford **9g** as a white solid. **M.p.**: 77–78 °C. **^{1}H NMR (400 MHz, $CDCl_3$):** δ = 7.92 – 7.86 (m, 2H), 7.75 (d, *J* = 8.3 Hz, 2H), 7.29 – 7.24 (m, 2H), 6.95 – 6.90 (m, 2H), 5.73 (q, *J* = 6.9 Hz, 1H), 3.88 (s, 3H), 2.41 (s, 3H), 1.58 (d, *J* = 6.9 Hz, 3H) ppm. **^{13}C NMR (101 MHz, $CDCl_3$):** δ = 193.2, 164.2, 145.1, 131.4, 129.9, 128.1, 126.7, 114.1, 77.4, 55.7, 21.8, 19.0 ppm. The spectroscopic data are in agreement with the literature.[8]

(*S*)-9g: Prepared by Method **A:** white solid (28 mg, 31%), **$[\alpha]_D$** ^{20} =-20 (c=0.1, $CHCl_3$). Enantiomeric excess is determined by HPLC YMC Chiral Amylose-C S-5μm (25 cm), (*n*-hexane/*i*-PrOH = 80/20, flow rate = 1.0 mL/min, 254 nm): major isomer: t_R = 17.66 min, minor isomer: t_R = 15.00 min, *ee* = 71%.

(*R*)-9g: Prepared by Method **B:** white solid (14 mg, 15%), **$[\alpha]_D$** ^{20} =+16.7 (c=0.12, $CHCl_3$). Enantiomeric excess is determined by HPLC YMC Chiral Amylose-C S-5μm (25 cm), (*n*-hexane/*i*-PrOH = 80/20, flow rate = 1.0 mL/min, 254 nm): major isomer: t_R = 14.97 min, minor isomer: t_R = 17.63 min, *ee* = 74%.

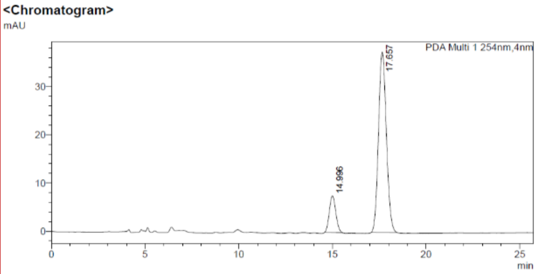

Peak Table

PDA Ch1 254nm

Peak#	Ret. Time	Area%
1	14.996	14.287
2	17.657	85.713
Total		100.000

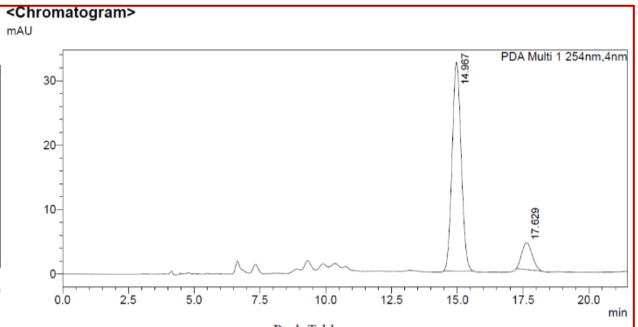

Peak Table

PDA Ch1 254nm

Peak#	Ret. Time	Area%
1	14.967	87.192
2	17.629	12.808
Total		100.000

1-(4-(*tert*-Butyl)phenyl)-1-oxopropan-2-yl 4-methylbenzenesulfonate (9h)

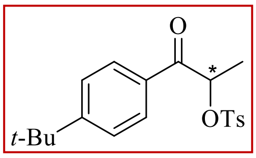

Following **GP 5** Method **A** or Method **B**, Chiral iodine catalyst (13 mg, 0.027 mmol), *m*CPBA (184 mg, 0.81 mmol), *p*-TsOH hydrate (154 mg, 0.81 mmol), and 3- *tert*-butylpropiophenone (**8h)** (51 mg, 0.27 mmol) to afford **9h** as a white solid. **M.p.**: 62–64 °C. **^{1}H NMR (400 MHz, $CDCl_3$):** δ = 7.85 – 7.79 (m, 2H), 7.79 – 7.72 (m, 2H), 7.48 – 7.43 (m, 2H), 7.27 – 7.22 (m, 2H), 5.78 (q, *J* = 6.9 Hz, 1H), 2.40 (s, 3H), 1.59 (d, *J* = 6.9 Hz, 3H), 1.34 (s, 9H) ppm. **^{13}C NMR (101 MHz, $CDCl_3$):** δ = 194.4, 157.9, 145.0, 133.7, 131.1, 129.8, 128.9, 128.1, 125.8, 77.6, 35.3, 31.1, 21.8, 18.9 ppm. **HRMS** (ESI): $[M+H]^+$ calc. 361.1474 for $[C_{20}H_{25}O_4S]^+$, found 361.1472. **IR** (neat): ν = 2932,1365, 1177, 1086, 921, 664 cm^{-1}.

(*S*)-9h: Prepared by Method **A:** white solid (19 mg, 20%), **$[\alpha]_D^{20}$** =-16.7 (c=0.12, $CHCl_3$). Enantiomeric excess is determined by HPLC YMC Chiral Amylose-C S-5µm (25 cm), (*n*-hexane/*i*-PrOH = 95/5, flow rate = 1.0 mL/min, 254 nm): major isomer: t_R = 8.7 min, minor isomer: t_R = 8.1 min, *ee* = 53%.

(*R*)-9h: Prepared by Method **B:** white solid (63 mg, 65%), **$[\alpha]_D^{20}$** =+5.9 (c=0.34, $CHCl_3$). Enantiomeric excess is determined by HPLC YMC Chiral Amylose-C S-5µm (25 cm), (*n*-hexane/*i*-PrOH = 95/5, flow rate = 1.0 mL/min, 254 nm): major isomer: t_R = 8.1 min, minor isomer: t_R = 8.8 min, *ee* = 66%.

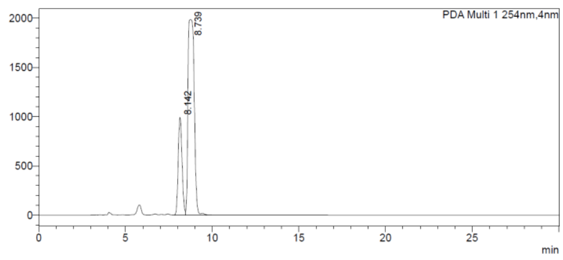

Peak Table

PDA Ch1 254nm

Peak#	Ret. Time	Area%
1	8.142	23.646
2	8.739	76.354
Total		100.000

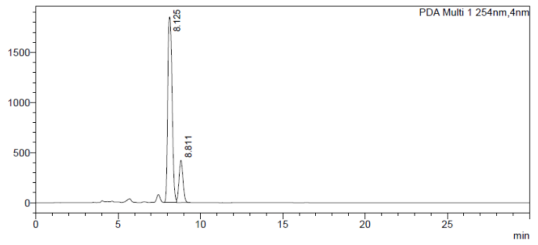

Peak Table

PDA Ch1 254nm

Peak#	Ret. Time	Area%
1	8.125	82.801
2	8.811	17.199
Total		100.000

1-(Naphthalen-2-yl)-1-oxopropan-2-yl 4-methylbenzenesulfonate (9i)

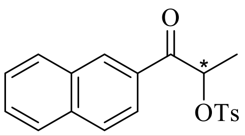

Following **GP 5** Method **A** and Method **B**, Chiral iodine catalyst (13 mg, 0.027 mmol), *m*CPBA (184 mg, 0.81 mmol), *p*-TsOH hydrate (154 mg, 0.81 mmol), and 1-naphthalen-2-yl-propan-1-one (**8i)** (50 mg, 0.27 mmol) to afford **9i** as a white solid. **^{1}H NMR (400 MHz, $CDCl_3$):** δ = 8.40 (s, 1H), 7.96 – 7.84 (m, 4H), 7.75 (d, *J* = 8.3 Hz, 2H), 7.66-7.55 (m, 2H), 7.22 – 7.18 (m, 2H), 5.97 – 5.90 (m, 1H), 2.36 (s, 3H), 1.67 (d, *J* = 6.9, 0.8 Hz, 3H) ppm. **^{13}C NMR (101 MHz, $CDCl_3$):** δ = 194.9, 145.1, 135.9, 133.6, 132.4, 131.1, 130.8, 129.9, 129.8, 129.2, 128.8, 128.0, 127.9, 127.1, 124.2, 77.6, 21.7, 19.0 ppm. The spectroscopic data are in agreement with the literature.[8]

(*S*)-9i: Prepared by Method **A:** white solid (58 mg, 60%), **$[\alpha]_D$** ^{20} = –10.5 (c = 0.19, $CHCl_3$). Enantiomeric excess is determined by HPLC YMC Chiral Amylose-C S-5μm (25 cm), (*n*-hexane/*i*-PrOH = 80/20, flow rate = 1.0 mL/min, 254 nm): major isomer: t_R = 13.68 min, minor isomer: t_R = 11.76 min, *ee* = 67%.

(*R*)-9i: Prepared by Method **B:** white solid (46 mg, 48%), **$[\alpha]_D$** ^{20} = +12.5 (c = 0.16, $CHCl_3$). Enantiomeric excess is determined by HPLC YMC Chiral Amylose-C S-5μm (25 cm), (*n*-hexane/*i*-PrOH = 80/20, flow rate = 1.0 mL/min, 254 nm): major isomer: t_R = 11.75 min, minor isomer: t_R = 13.67 min, *ee* = 78%.

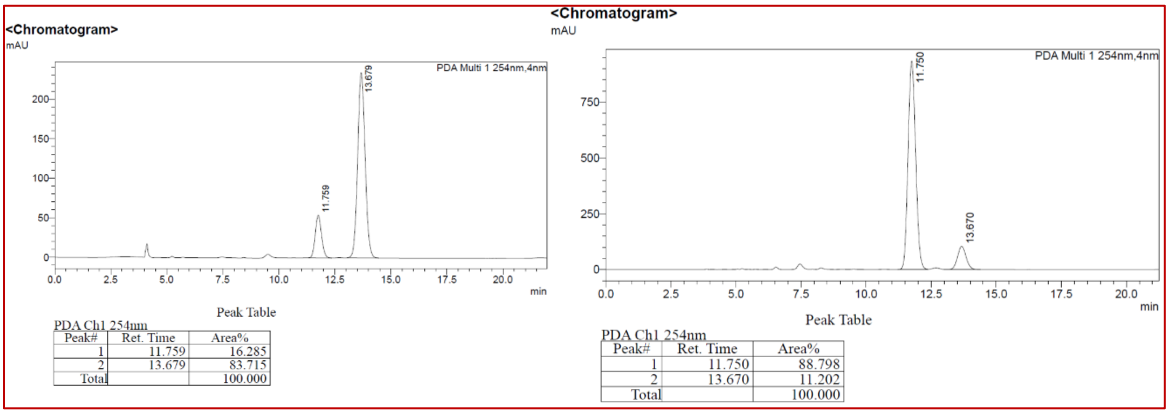

1-Oxo-1-phenylbutan-2-yl 4-methylbenzenesulfonate (9j)

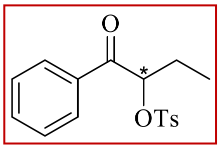

Following **GP 5** Method **A** and Method **B**, chiral iodine catalyst (13 mg, 0.027 mmol), *m*CPBA (184 mg, 0.81 mmol), *p*-TsOH hydrate (154 mg, 0.81 mmol), and butyrophenone (**8j)** (40 mg, 0.27 mmol) to afford **9j** as a white solid. **m.p.**: 63-65 °C. **^{1}H NMR (400 MHz, $CDCl_3$):** δ = 7.88 – 7.82 (m, 2H), 7.74 (d, *J* = 8.3 Hz, 2H), 7.58 (ddd, *J* = 7.0, 2.5, 1.2 Hz, 1H), 7.45 (dd, *J* = 7.9, 7.5 Hz, 2H), 7.24 (dd, *J* = 8.6, 0.6 Hz, 2H), 5.56 (dd, *J* = 7.8, 5.0 Hz, 1H), 2.40 (s, 3H), 2.04 – 1.88 (m, 2H), 0.98 (t, *J* = 7.4 Hz, 3H) ppm. **^{13}C NMR (101 MHz, $CDCl_3$):** δ = 195.0, 145.1, 134.3, 133.9, 133.5, 129.8, 128.9, 128.8, 128.2, 82.7, 26.4, 21.8, 9.7 ppm. The spectroscopic data are in agreement with the literature.[8]

(*S*)-9j: Prepared by Method **A:** white solid (81 mg, 94%), **$[\alpha]_D$ ^{20}** = –20 (c = 8.1, $CHCl_3$). Enantiomeric excess is determined by HPLC YMC Chiral Amylose-C S-5μm (25 cm), (*n*-hexane/*i*-PrOH = 80/20, flow rate = 1.0 mL/min, 254 nm): major isomer: t_R = 9.23 min, minor isomer: t_R = 8.14 min, *ee* = 77%.

(*R*)-9j: Prepared by Method **B:** white solid (78 mg, 91%), **$[\alpha]_D$ ^{20}** = +7.4 (c = 1.08, $CHCl_3$). Enantiomeric excess is determined by HPLC YMC Chiral Amylose-C S-5μm (25 cm), (*n*-hexane/*i*-PrOH = 80/20, flow rate = 1.0 mL/min, 254 nm): major isomer: t_R = 8.18 min, minor isomer: t_R = 9.29 min, *ee* = 80%.

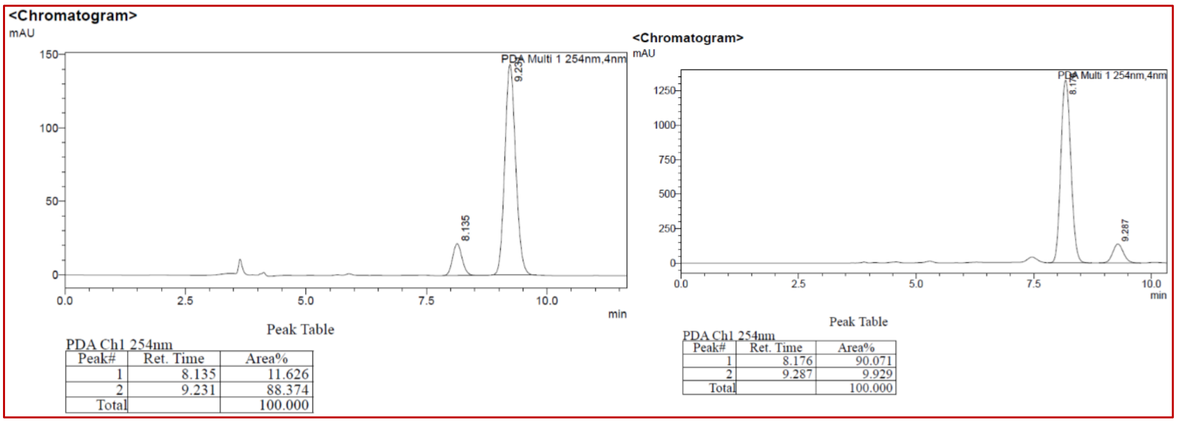

2-Oxo-1,2-diphenylethyl 4-methylbenzenesulfonate (9k)

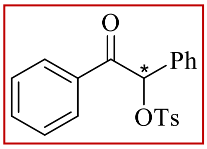

Following **GP 5** Method **A** and Method **B**, Chiral iodine catalyst (13 mg, 0.027 mmol), *m*CPBA (184 mg, 0.81 mmol), *p*-TsOH hydrate (154 mg, 0.81 mmol), and deoxy benzoin (**8k**) (53 mg, 0.27 mmol) to afford **9k** as a white solid. **M.p.**: 95–96 °C. **^{1}H NMR (400 MHz, $CDCl_3$):** δ = 7.84 (dd, *J* = 5.2, 3.3 Hz, 2H), 7.74 – 7.69 (m, 2H), 7.54 – 7.48 (m, 1H), 7.39 – 7.33 (m, 4H), 7.32 – 7.27 (m, 3H), 7.24 – 7.20 (m, 2H), 6.67 (s, 1H), 2.39 (s, 3H) ppm. **^{13}C NMR (101 MHz, $CDCl_3$):** δ = 192.2, 145.0, 134.2, 133.8, 133.7, 132.8, 129.7, 129.2, 129.1, 128.8, 128.3, 128.2, 82.4, 21.8 ppm. The spectroscopic data are in agreement with the literature.[8]

(*S*)-9k: Prepared by Method **A:** white solid (18.8 mg, 19%), Enantiomeric excess is determined by HPLC YMC Chiral Amylose-C S-5μm (25 cm), (*n*-hexane/*i*-PrOH = 80/20, flow rate = 1.0 mL/min, 254 nm): major isomer: t_R = 15.26 min, minor isomer: t_R = 18.68 min, *ee* = 3%.

(*R*)-9k: Prepared by Method **B:** white solid (39.6 mg, 40%), Enantiomeric excess is determined by HPLC YMC Chiral Amylose-C S-5μm (25 cm), (*n*-hexane/*i*-PrOH = 80/20, flow rate = 1.0 mL/min, 254 nm): major isomer: t_R = 18.69 min, minor isomer: t_R = 15.27 min, *ee* = 4%.

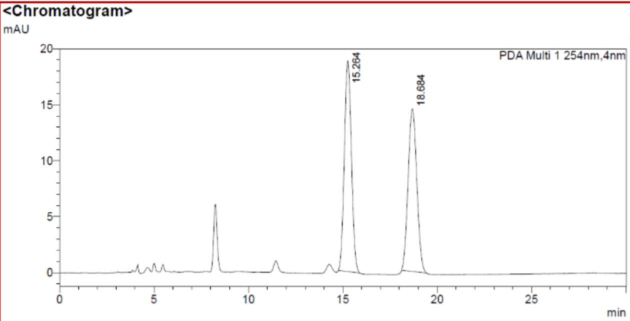

Peak Table

PDA Ch1 254nm

Peak#	Ret. Time	Area%
1	15.264	51.309
2	18.684	48.691
Total		100.000

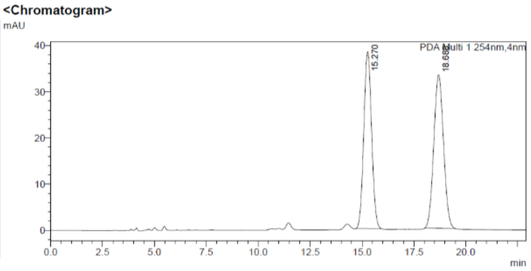

Peak Table

PDA Ch1 254nm

Peak#	Ret. Time	Area%
1	15.270	48.175
2	18.688	51.825
Total		100.000

1-(Furan-2-yl)-1-oxopropan-2-yl 4-methylbenzenesulfonate (9l)

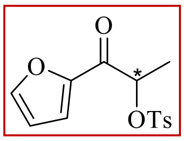

Following **GP 5** Method **A** and **B**, chiral iodine catalyst (13 mg, 0.027 mmol), *m*CPBA (184 mg, 0.81 mmol), *p*-TsOH hydrate (154 mg, 0.81 mmol), and 2-propionylfuran (**8l)** (34 mg, 0.27 mmol) to afford **9l** as a colourless oil. **^{1}H NMR (400 MHz, CDCl$_3$):** δ = 7.78 (d, *J* = 8.3 Hz, 2H), 7.61 (dd, *J* = 1.6, 0.6 Hz, 1H), 7.36 (dd, *J* = 3.6, 0.5 Hz, 1H), 7.30 (d, *J* = 8.0 Hz, 2H), 6.56 (dd, *J* = 3.6, 1.7 Hz, 1H), 5.51 (q, *J* = 6.9 Hz, 1H), 2.42 (s, 3H), 1.55 (d, *J* = 6.9 Hz, 3H) ppm. **^{13}C NMR (101 MHz, CDCl$_3$):** δ = 183.5, 149.7, 147.6, 145.3, 133.5, 130.0, 128.1, 120.2, 112.8, 77.5, 21.8, 18.6 ppm. The spectroscopic data are in agreement with the literature.[8]

(S)-9l: Prepared by Method **A:** colourless oil (73.5 mg, 93%), **$[\alpha]_D$ ^{20}** =- 18.5 (c=0.54, CHCl$_3$). Enantiomeric excess is determined by HPLC YMC Chiral Amylose-C S-5µm (25 cm), (*n*-hexane/*i*-PrOH = 80/20, flow rate = 1.0 mL/min, 254 nm): major isomer: t_R = 10.5 min, minor isomer: t_R = 9.9 min, *ee* = 57%.

(R)-9l: Prepared by Method **B:** colourless oil (52 mg, 66%), **$[\alpha]_D$ ^{20}** =+ 13.3 (c=0.15, CHCl$_3$). Enantiomeric excess is determined by HPLC YMC Chiral Amylose-C S-5µm (25 cm), (*n*-hexane/*i*-PrOH = 80/20, flow rate = 1.0 mL/min, 254 nm): major isomer: t_R = 10.0 min, minor isomer: t_R = 10.6 min, *ee* = 76%.

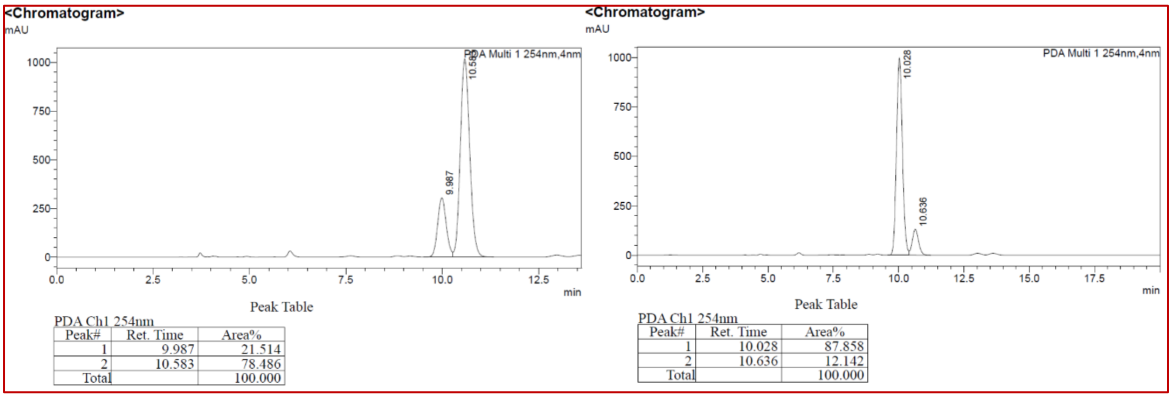

1-Oxo-1-(thiophen-2-yl)propan-2-yl 4-methylbenzenesulfonate (9m)

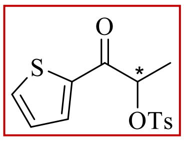

Following **GP 5** Method **A** and Method **B**, chiral iodine catalyst (13 mg, 0.027 mmol), *m*CPBA (184 mg, 0.81 mmol), *p*-TsOH hydrate (154 mg, 0.81 mmol), and 2-propionylthiophene (**8m)** (38 mg, 0.27 mmol) to afford **9m** as a colourless oil. **^{1}H NMR (400 MHz, $CDCl_3$):** δ = 7.88 (dd, *J* = 3.9, 0.9 Hz, 1H), 7.76 (d, *J* = 8.4 Hz, 2H), 7.71 (dd, *J* = 4.9, 1.0 Hz, 1H), 7.28 (d, *J* = 8.0 Hz, 2H), 7.15 (dd, *J* = 4.9, 3.9 Hz, 1H), 5.45 (q, *J* = 6.9 Hz, 1H), 2.42 (s, 3H), 1.60 (d, *J* = 6.9 Hz, 3H) ppm. **^{13}C NMR (101 MHz, $CDCl_3$):** δ = 188.2, 145.4, 140.0, 135.4, 134.0, 133.3, 130.0, 128.6, 128.2, 78.7, 21.8, 19.3 ppm. The spectroscopic data are in agreement with the literature.[8]

(*S*)-9m: Prepared by Method **A:** colourless oil (7 mg, 8%), **$[\alpha]_D^{20}$** = –40 (c = 0.1, $CHCl_3$). Enantiomeric excess is determined by HPLC YMC Chiral Amylose-C S-5μm (25 cm), (*n*-hexane/*i*-PrOH = 80/20, flow rate = 1.0 mL/min, 254 nm): major isomer: t_R = 10.81 min, minor isomer: t_R = 9.57 min, *ee* = 65%.

(*R*)-9m: Prepared by Method **B:** colourless oil (13 mg, 15%), **$[\alpha]_D^{20}$** = +20 (c = 0.1, $CHCl_3$). Enantiomeric excess is determined by HPLC YMC Chiral Amylose-C S-5μm (25 cm), (*n*-hexane/*i*-PrOH = 80/20, flow rate = 1.0 mL/min, 254 nm): major isomer: t_R = 9.60 min, minor isomer: t_R = 10.86 min, *ee* = 80%.

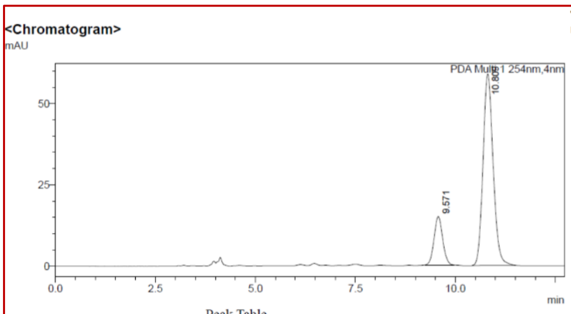

Peak Table

PDA Ch1 254nm

Peak#	Ret. Time	Area%
1	9.571	17.711
2	10.809	82.289
Total		100.000

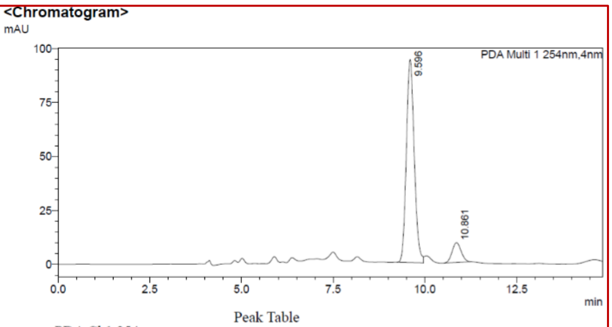

Peak Table

PDA Ch1 254nm

Peak#	Ret. Time	Area%
1	9.596	89.876
2	10.861	10.124
Total		100.000

1-Oxo-2,3-dihydro-1H-inden-2-yl 4-methylbenzenesulfonate (9n)

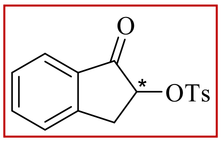

Following **GP 5** Method **A** and Method **B**, Chiral iodine catalyst (13 mg, 0.027 mmol), *m*CPBA (184 mg, 0.81 mmol), *p*-TsOH hydrate (154 mg, 0.81 mmol), and 1-indanone (**8n)** (36 mg, 0.27 mmol) to afford **9n** as a white solid. **M.p.**: 108–109 °C. **^{1}H NMR (400 MHz, CDCl$_3$):** δ = 7.93 (d, *J* = 8.3 Hz, 2H), 7.73 (d, *J* = 7.7 Hz, 1H), 7.65 (td, *J* = 7.7, 1,1 Hz, 1H), 7.46 – 7.41 (m, 1H), 7.41 – 7.36 (m, 3H), 5.13 (dd, *J* = 8.0, 4.8 Hz, 1H), 3.66 (dd, *J* = 17.3, 7.9 Hz, 1H), 3.28 (dd, *J* = 17.2, 4.7 Hz, 1H), 2.47 (s, 3H) ppm. **^{13}C NMR (101 MHz, CDCl$_3$):** δ = 197.7, 150.1, 145.3, 136.5, 133.8, 133.4, 130.0, 128.6, 128.4, 126.8, 124.9, 78.4, 34.1, 21.9 ppm. The spectroscopic data are in agreement with the literature.[6]

(*S*)-9n: Prepared by Method **A:** white solid (77 mg, 94%), **$[\alpha]_D^{20}$** = -2.1 (c=1.9, CHCl$_3$). Enantiomeric excess is determined by HPLC YMC Chiral Amylose-C S-5µm (25 cm), (*n*-hexane/*i*-PrOH = 80/20, flow rate = 1.0 mL/min, 254 nm): major isomer: t_R = 18.07 min, minor isomer: t_R = 17.07 min, *ee* = 48%.

(*R*)-9n: Prepared by Method **B:** white solid (55 mg, 67%), **$[\alpha]_D^{20}$** = +3.3 (c=1.2, CHCl$_3$). Enantiomeric excess is determined by HPLC YMC Chiral Amylose-C S-5µm (25 cm), (*n*-hexane/*i*-PrOH = 80/20, flow rate = 1.0 mL/min, 254 nm): major isomer: t_R = 17.15 min, minor isomer: t_R = 18.17 min, *ee* = 62%.

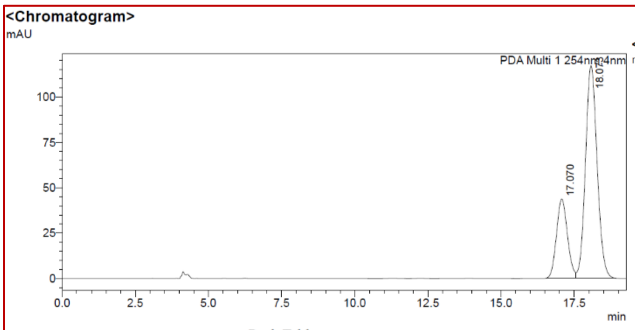

Peak Table

PDA Ch1 254nm

Peak#	Ret. Time	Area%
1	17.070	25.865
2	18.073	74.135
Total		100.000

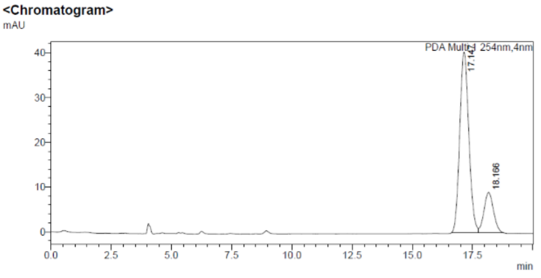

Peak Table

PDA Ch1 254nm

Peak#	Ret. Time	Area%
1	17.147	81.080
2	18.166	18.920
Total		100.000

1-Oxo-1,2,3,4-tetrahydronaphthalen-2-yl 4-methylbenzenesulfonate (9o)

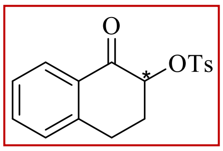

Following **GP 5** Method **A** and Method **B**, Chiral iodine catalyst (13 mg, 0.027 mmol), *m*CPBA (184 mg, 0.81 mmol), *p*-TsOH hydrate (154 mg, 0.81 mmol), and 1-tetralone (**8o)** (39 mg, 0.27 mmol) to afford **9o** as a colourless oil. **^{1}H NMR (400 MHz, $CDCl_3$):** δ = 7.95 (dd, *J* = 7.9, 0.9 Hz, 1H), 7.91 (d, *J* = 8.3 Hz, 2H), 7.50 (td, *J* = 7.6, 1.4 Hz, 1H), 7.36 (dd, *J* = 8.6, 0.6 Hz, 2H), 7.34 – 7.28 (m, 1H), 7.27 – 7.22 (m, 1H), 5.16 (dd, *J* = 12.2, 4.9 Hz, 1H), 3.13 (dd, *J* = 10.0, 4.0 Hz, 2H), 2.56 (dq, *J* = 13.5, 4.4 Hz, 1H), 2.48 – 2.35 (m, 1H), 2.45 (s, 3H) ppm. **^{13}C NMR (101 MHz, $CDCl_3$):** δ = 190.6, 145.0, 143.0, 134.4, 133.8, 131.3, 129.9, 128.8, 128.3, 128.2, 127.3, 80.2, 30.7, 27.5, 21.8 ppm. The spectroscopic data are in agreement with the literature.[8]

(*R*)-9o: Prepared by Method **A:** colourless oil (27 mg, 32%), Enantiomeric excess is determined by HPLC YMC Chiral Amylose-C S-5µm (25 cm), (*n*-hexane/*i*-PrOH = 80/20, flow rate = 1.0 mL/min, 254 nm): major isomer: t_R = 13.86 min, minor isomer: t_R = 14.51 min, ee = 3%.

(*S*)-9o: Prepared by Method **B:** colourless oil (14 mg, 17%), Enantiomeric excess is determined by HPLC YMC Chiral Amylose-C S-5µm (25 cm), (*n*-hexane/*i*-PrOH = 80/20, flow rate = 1.0 mL/min, 254 nm): major isomer: t_R = 14.59 min, minor isomer: t_R = 13.92 min, *ee* = 20%.

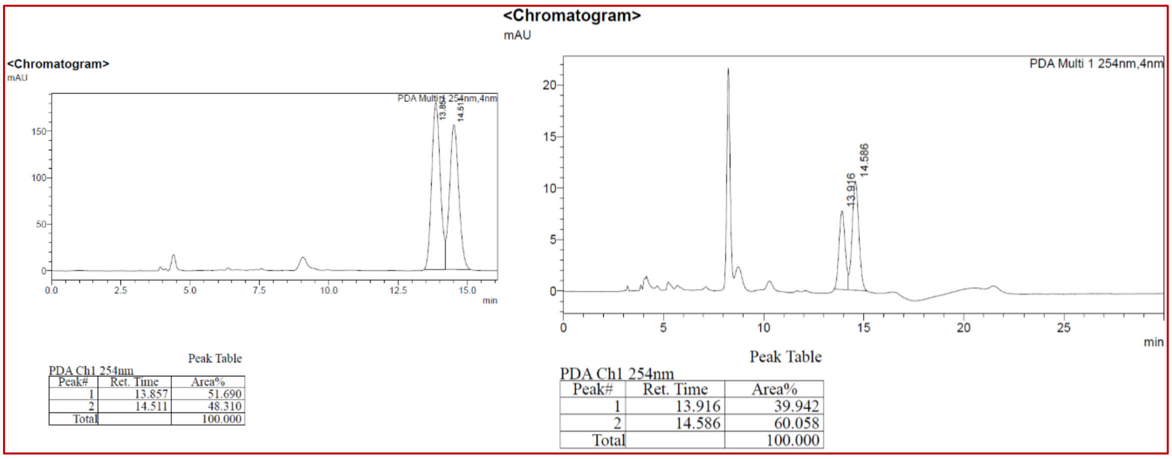

1-Oxo-1-phenylpropan-2-yl benzenesulfonate (9q)

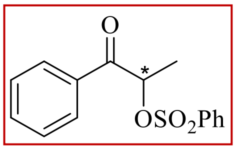

Following **GP 5** Method **A** or Method **B**, Chiral iodine catalyst (13 mg, 0.027 mmol), *m*CPBA (184 mg, 0.81 mmol), benzenesulfonic acid (128 mg, 0.81 mmol), and propiophenone (**8a)** (36.4 mg, 0.27 mmol) to afford **9q** as a white solid. **M.p.**: 62–63 °C. **^{1}H NMR (400 MHz, $CDCl_3$):** δ = 7.90 – 7.88 (m, 2H), 7.87 (dd, *J* = 3.5, 1.3 Hz, 2H), 7.64 – 7.56 (m, 2H), 7.52 – 7.43 (m, 4H), 5.83 (q, *J* = 6.9 Hz, 1H), 1.61 (d, *J* = 6.9 Hz, 3H) ppm. **^{13}C NMR (101 MHz, $CDCl_3$):** δ = 194.8, 136.7, 134.1, 134.0, 133.8, 129.3, 129.0, 128.9, 128.1, 77.7, 18.9 ppm. The spectroscopic data are in agreement with the literature.[6]

(*S*)-9q: Prepared by Method **A:** white solid (75 mg, 96%), **$[\alpha]_D^{20}$** = –1.0 (c = 1.90, $CHCl_3$). Enantiomeric excess is determined by HPLC YMC Chiral Amylose-C S-5μm (25 cm), (*n*-hexane/*i*-PrOH = 80/20, flow rate = 1.0 mL/min, 254 nm): major isomer: t_R = 9.78 min, minor isomer: t_R = 9.07 min, *ee* = 70%.

(*R*)-9q: Prepared by Method **B:** white solid (73 mg, 94%), **$[\alpha]_D^{20}$** = +1.8 (c = 2.24, $CHCl_3$). Enantiomeric excess is determined by HPLC YMC Chiral Amylose-C S-5μm (25 cm), (*n*-hexane/*i*-PrOH = 80/20, flow rate = 1.0 mL/min, 254 nm): major isomer: t_R = 9.07 min, minor isomer: t_R = 9.78 min, *ee* = 77%.

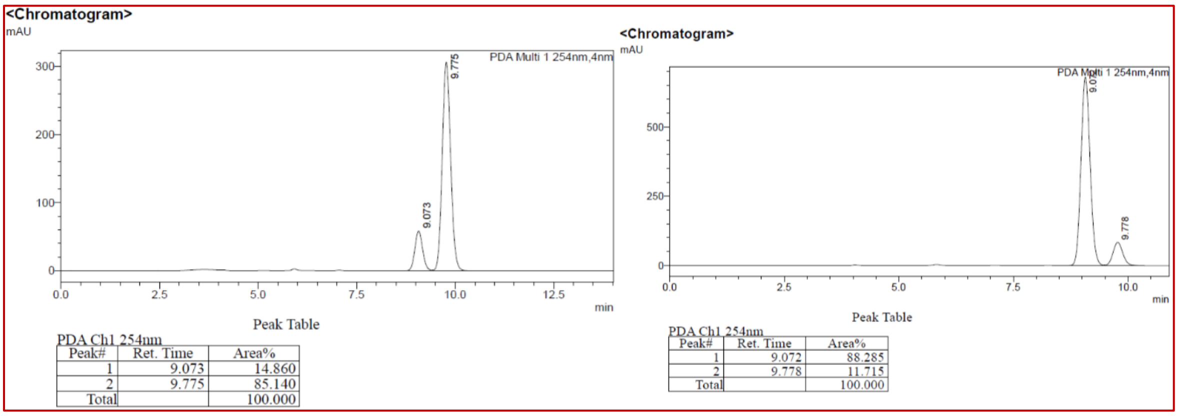

PDA Ch1 254nm

Peak#	Ret. Time	Area%
1	9.073	14.860
2	9.775	85.140
Total		100.000

PDA Ch1 254nm

Peak#	Ret. Time	Area%
1	9.072	88.285
2	9.778	11.715
Total		100.000

1-Oxo-1-phenylpropan-2-yl methanesulfonate (9r)

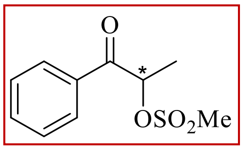

Following **GP 5** Method **A** or Method **B**, Chiral iodine catalyst (13 mg, 0.027 mmol), *m*CPBA (184 mg, 0.81 mmol), methanesulfonic acid (78 mg, 0.81 mmol), and propiophenone (**8a)** (36.4 mg, 0.27 mmol) to afford **9r** as a white solid. **M.p.**: 76–78 °C. **^{1}H NMR (400 MHz, $CDCl_3$):** δ = 7.97-7.91 (m, 2H), 7.68 – 7.59 (m, 1H), 7.55-7.48 (m, 2H), 6.05 (q, *J* = 7.0 Hz, 1H), 3.14 (s, 3H), 1.67 (d, *J* = 7.0 Hz, 3H) ppm. **^{13}C NMR (101 MHz, $CDCl_3$):** δ = 193.7, 134.3, 129.2, 128.8, 77.4, 39.6, 18.9 ppm. The spectroscopic data are in agreement with the literature.[6]

(*S*)-9r: Prepared by Method **A:** white solid (59.5 mg, 96%), **$[\alpha]_D^{20}$** = –3.0 (c = 0.66, $CHCl_3$). Enantiomeric excess is determined by HPLC YMC Chiral Amylose-C S-5µm (25 cm), (*n*-hexane/*i*-PrOH = 95/5, flow rate = 1.0 mL/min, 254 nm): major isomer: t_R = 18.9 min, minor isomer: t_R = 19.8 min, *ee* = 65%.

(*R*)-9r: Prepared by Method **B:** white solid (51 mg, 82%), **$[\alpha]_D^{20}$** = +9.0 (c = 0.22, $CHCl_3$). Enantiomeric excess is determined by HPLC YMC Chiral Amylose-C S-5µm (25 cm), (*n*-hexane/*i*-PrOH = 95/5, flow rate = 1.0 mL/min, 254 nm): major isomer: t_R = 19.8 min, minor isomer: t_R = 18.9 min, *ee* = 66%.

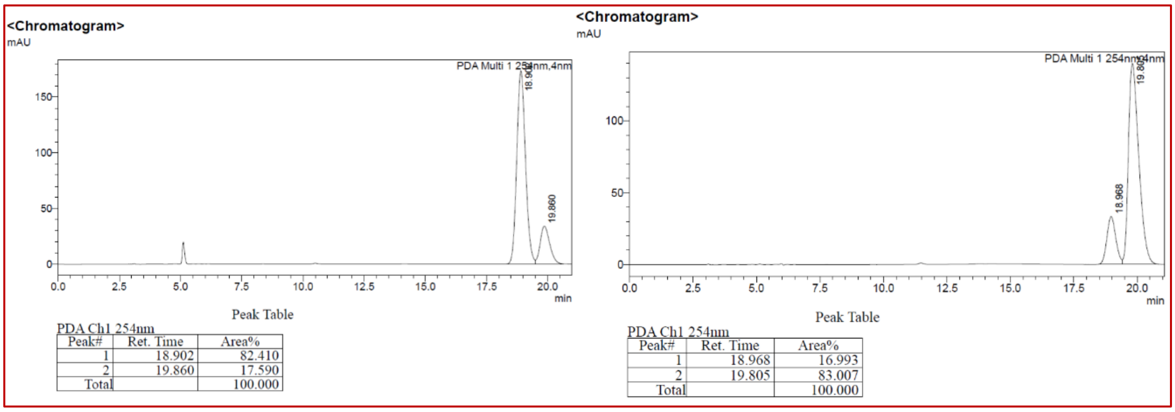

PDA Ch1 254nm

Peak#	Ret. Time	Area%
1	18.902	82.410
2	19.860	17.590
Total		100.000

PDA Ch1 254nm

Peak#	Ret. Time	Area%
1	18.968	16.993
2	19.805	83.007
Total		100.000

References

[1] R. Adepu, A. Rajitha, D. Ahuja, A. K. Sharma, B. Ramudu, R. Kapavarapu, K. V. L. Parsa, M. Pal, *Org. Biomol. Chem.* **2014**, *12*, 2514.

[2] D. B. Guthrie, S. J. Geib, D. P. Curran, *J. Am. Chem. Soc.* **2009**, *131*, 15492–15500.

[3] S. Yang, W. Chung, **1999**, *38*, 897–904.

[4] A. Le Pera, A. Leggio, A. Liguori, *Tetrahedron* **2006**, *62*, 6100–6106.

[5] P. Yang, X. Wang, L. Peng, F. Chen, F. Tian, C.-Z. Tang, L.-X. Wang, *Org. Process Res. Dev.* **2017**, *21*, 1682–1688.

[6] S. M. Altermann, R. D. Richardson, T. K. Page, R. K. Schmidt, E. Holland, U. Mohammed, S. M. Paradine, A. N. French, C. Richter, A. M. Bahar, et al., *Eur. J. Org. Chem.* **2008**, *2*, 5315–5328.

[7] A. A. Guilbault, B. Basdevant, V. Wanie, C. Y. Legault, *J. Org. Chem.* **2012**, *77*, 11283–11295.

[8] J. Yu, J. Cui, X. Sen Hou, S. S. Liu, W. C. Gao, S. Jiang, J. Tian, C. Zhang, *Tetrahedron Asymmetry* **2011**, *22*, 2039–2055.

[9] G. Levitre, A. Dumoulin, P. Retailleau, A. Panossian, F. R. Leroux, G. Masson, *J. Org. Chem.* **2017**, *82*, 11877–11883.

NMR spectra

2-Iodo-4,6-dimethylaniline (**5a**), ^{1}H NMR

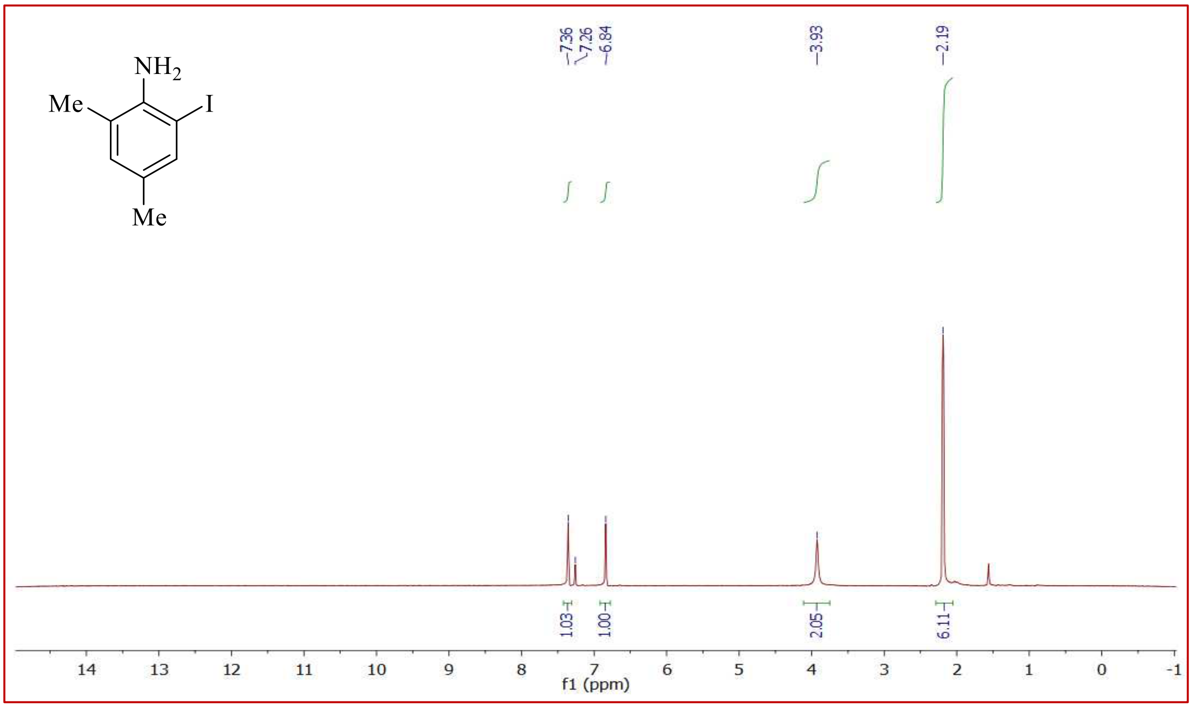

2-Iodo-4,6-dimethylaniline (**5a**), ^{13}C NMR

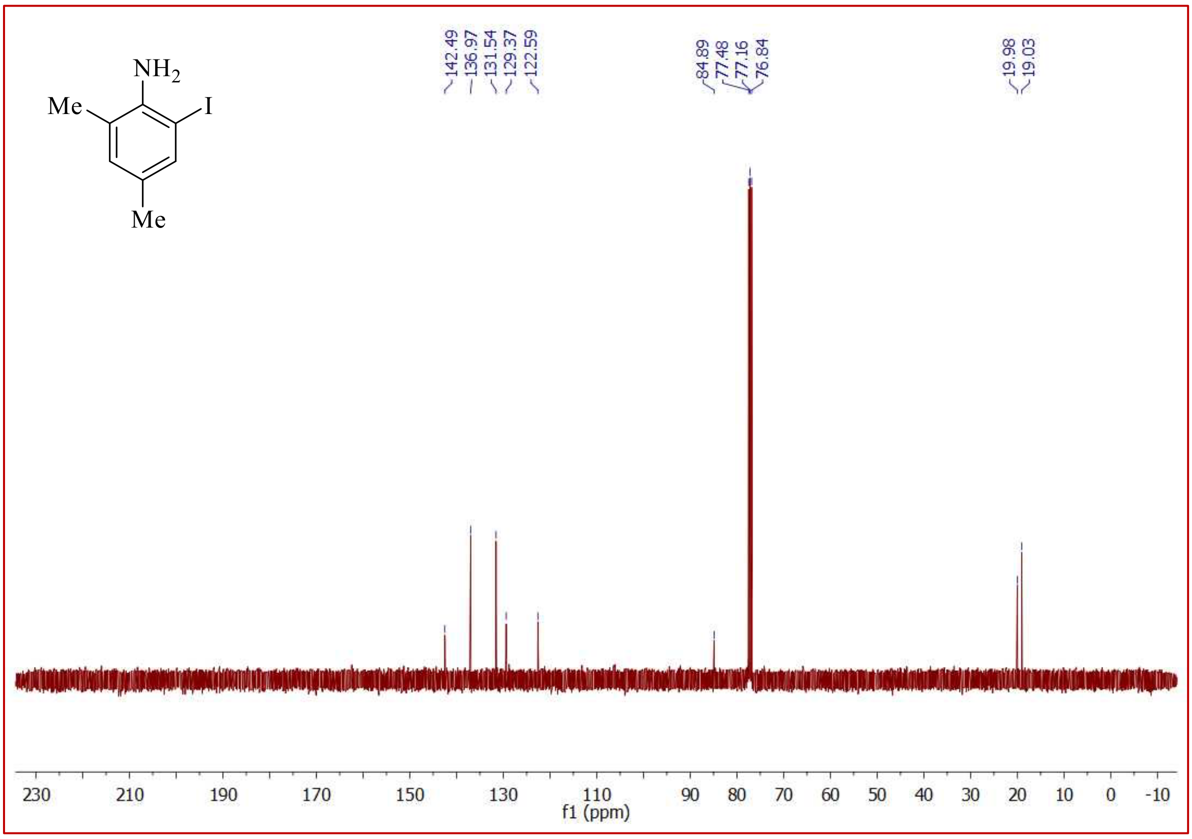

4-Chloro-2-iodo-6-methylaniline (**5b**), ^{1}H NMR

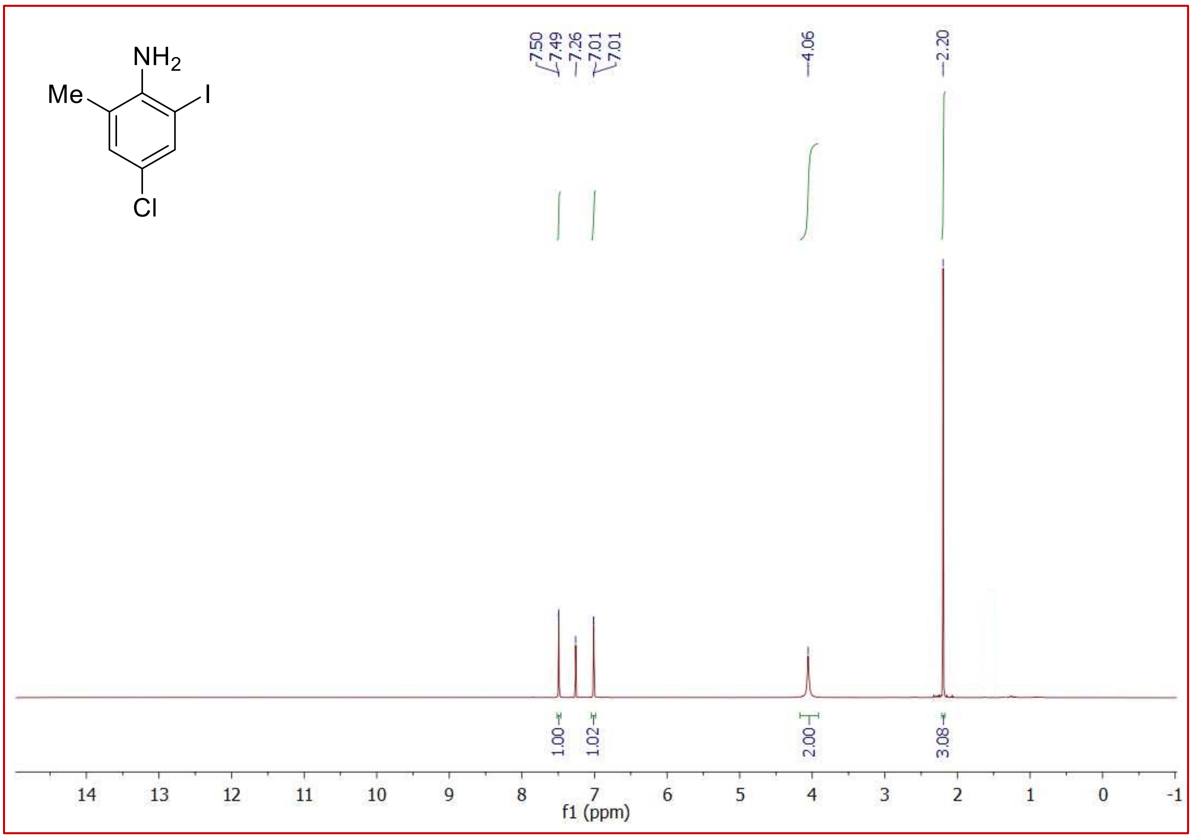

4-Chloro-2-iodo-6-methylaniline (**5b**), ^{13}C NMR

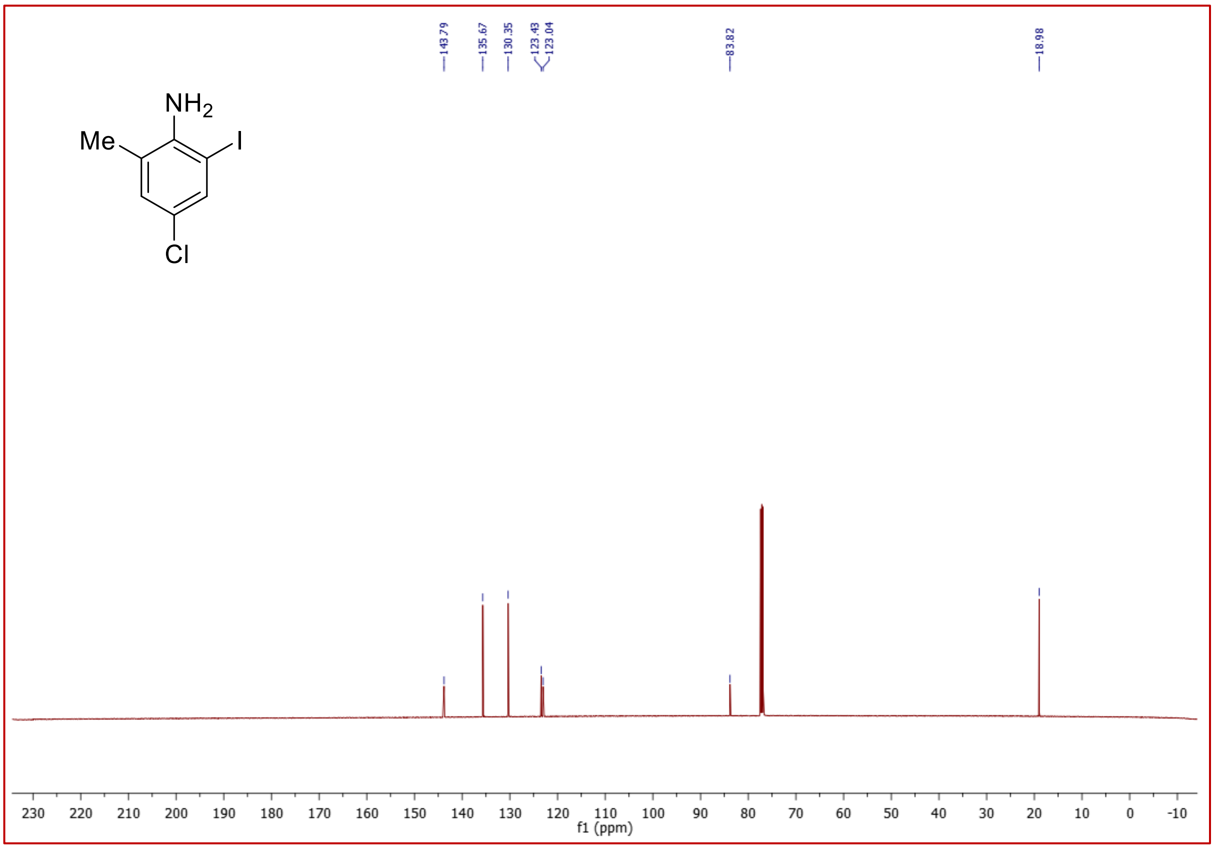

N-(2-Iodo-4,6-dimethylphenyl)-4-methylbenzenesulfonamide (**6a**), ^{1}H NMR

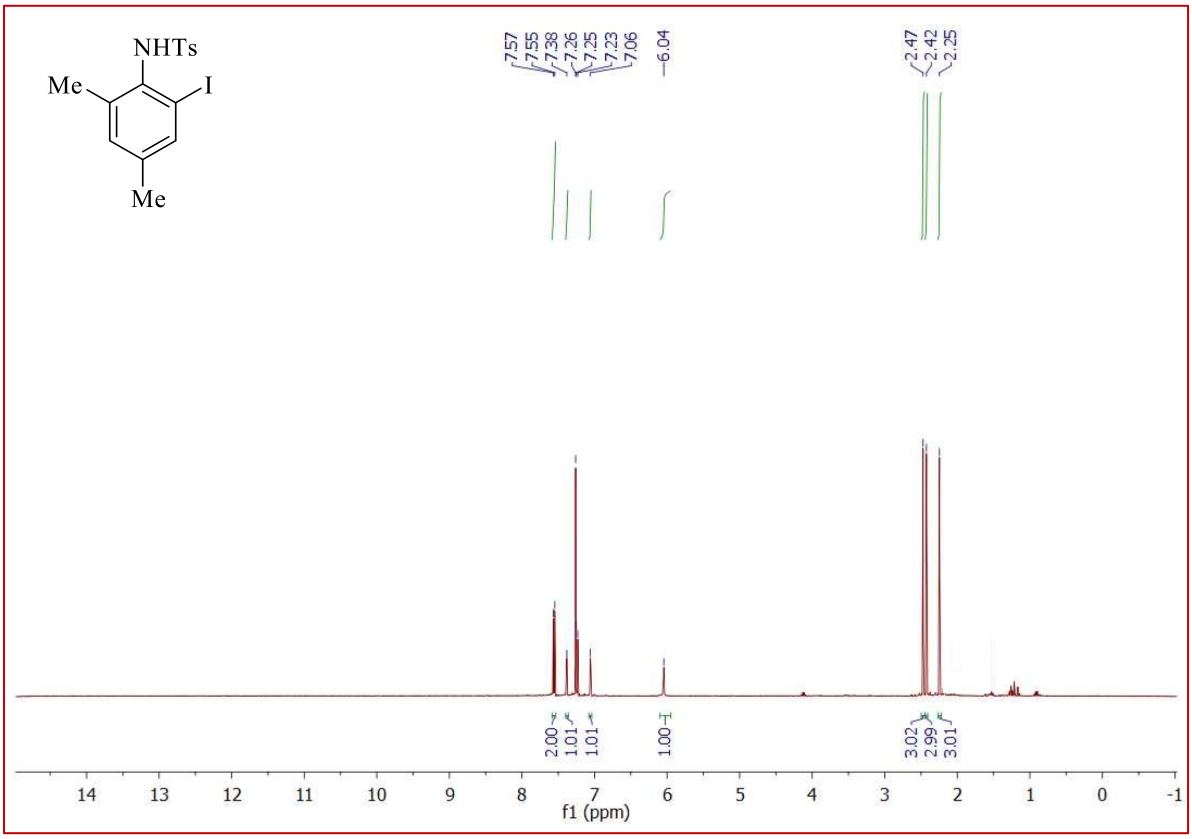

N-(2-Iodo-4,6-dimethylphenyl)-4-methylbenzenesulfonamide (**6a**), ^{13}C NMR

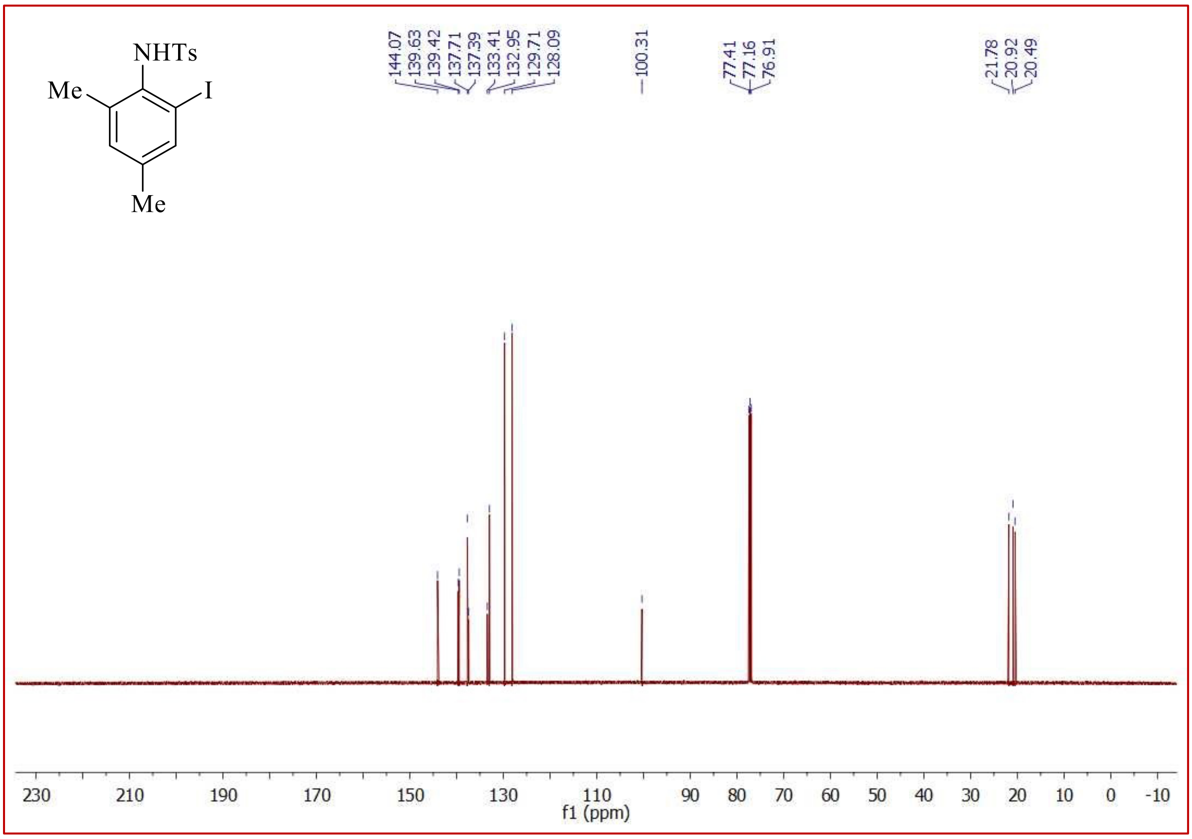

N-(2-Iodo-4,6-dimethylphenyl)-4-nitrobenzenesulfonamide (**6b**), ^{1}H NMR

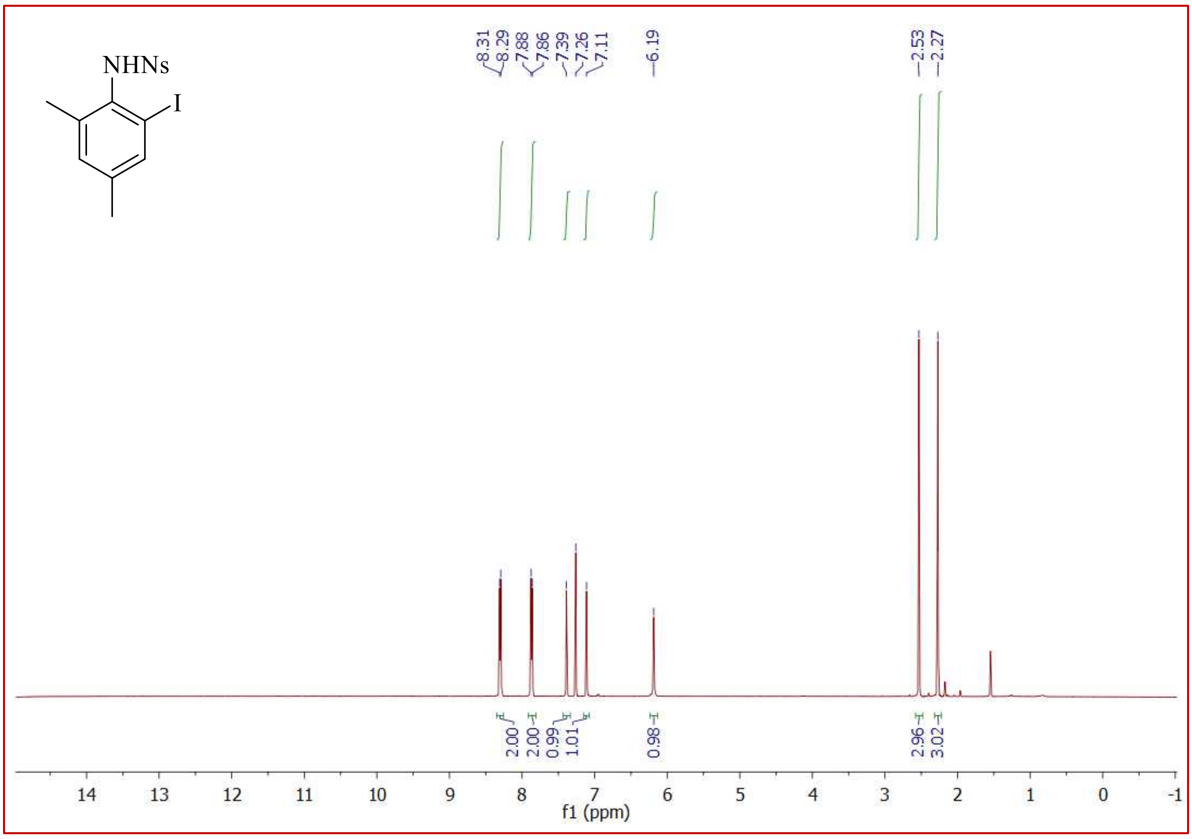

N-(2-Iodo-4,6-dimethylphenyl)-4-nitrobenzenesulfonamide (**6b**), ^{13}C NMR

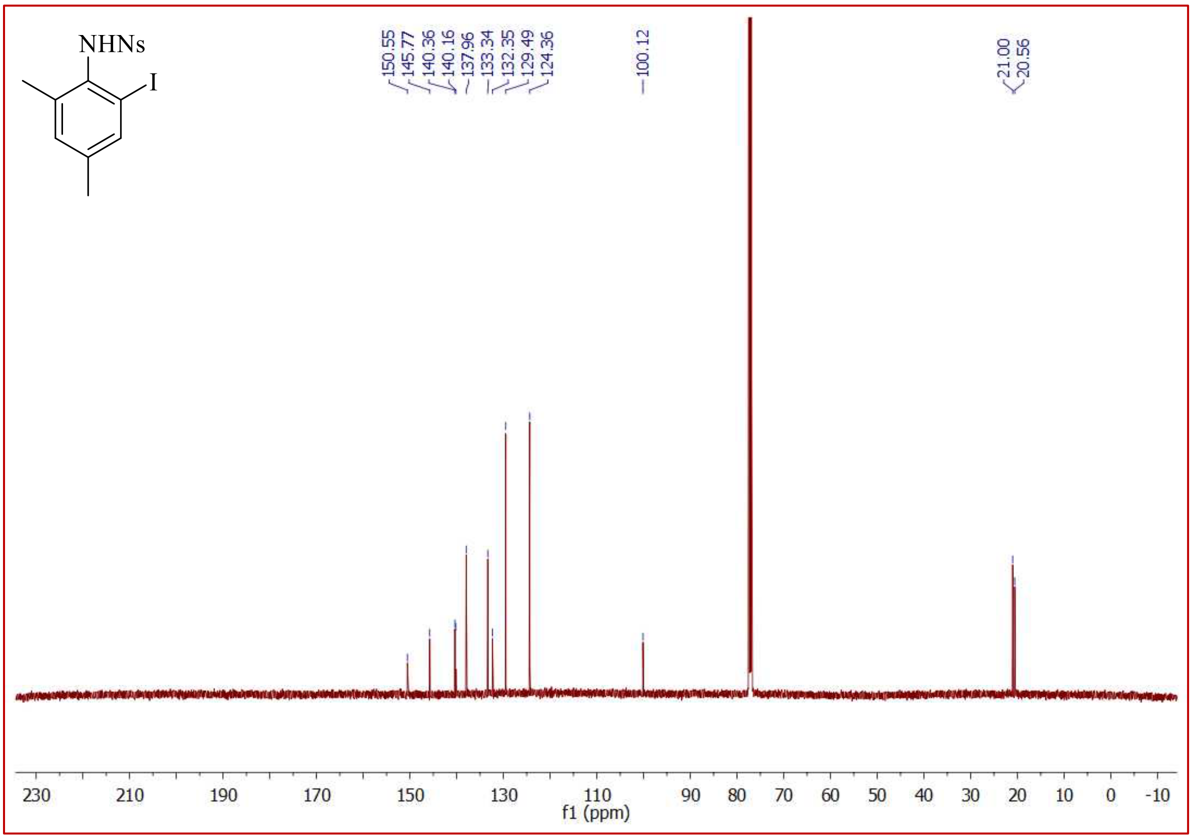

N-(2-Iodo-4,6-dimethylphenyl)-4-methoxybenzenesulfonamide (**6c**), ^{1}H NMR

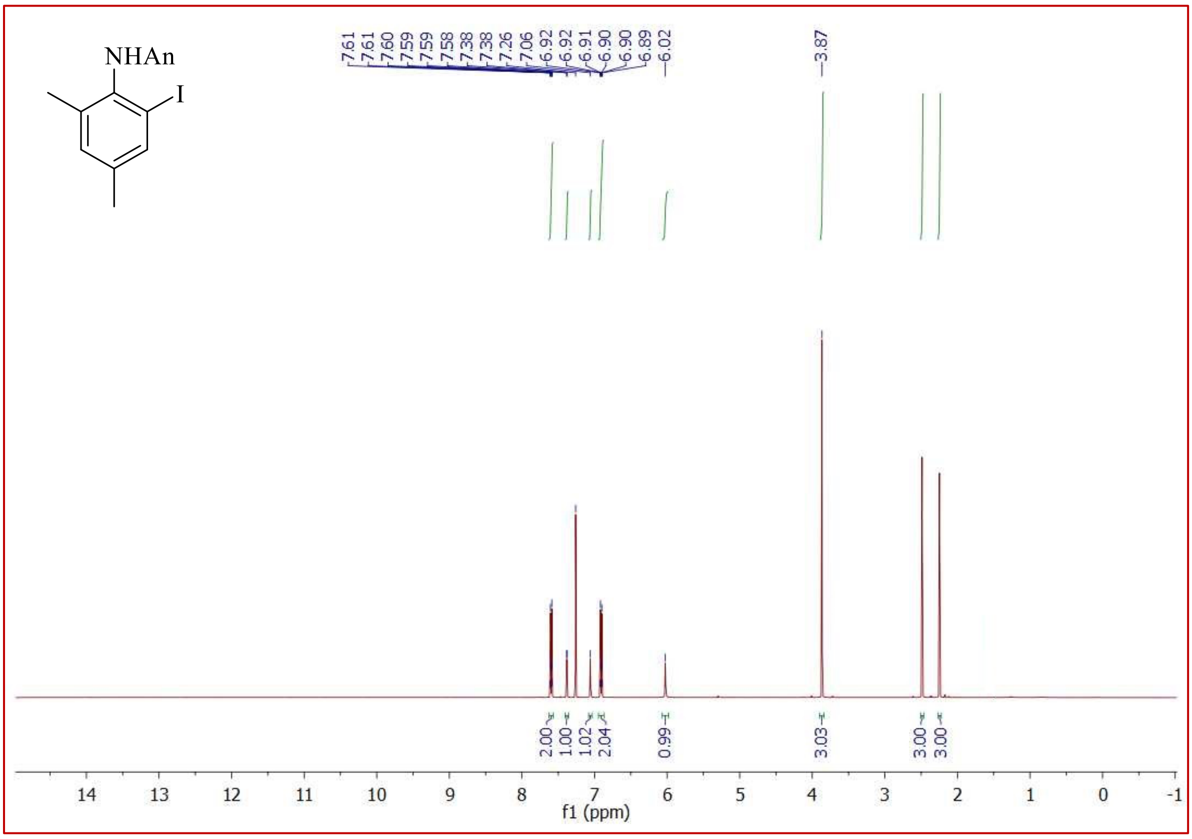

N-(2-Iodo-4,6-dimethylphenyl)-4-methoxybenzenesulfonamide (**6c**), ^{13}C NMR

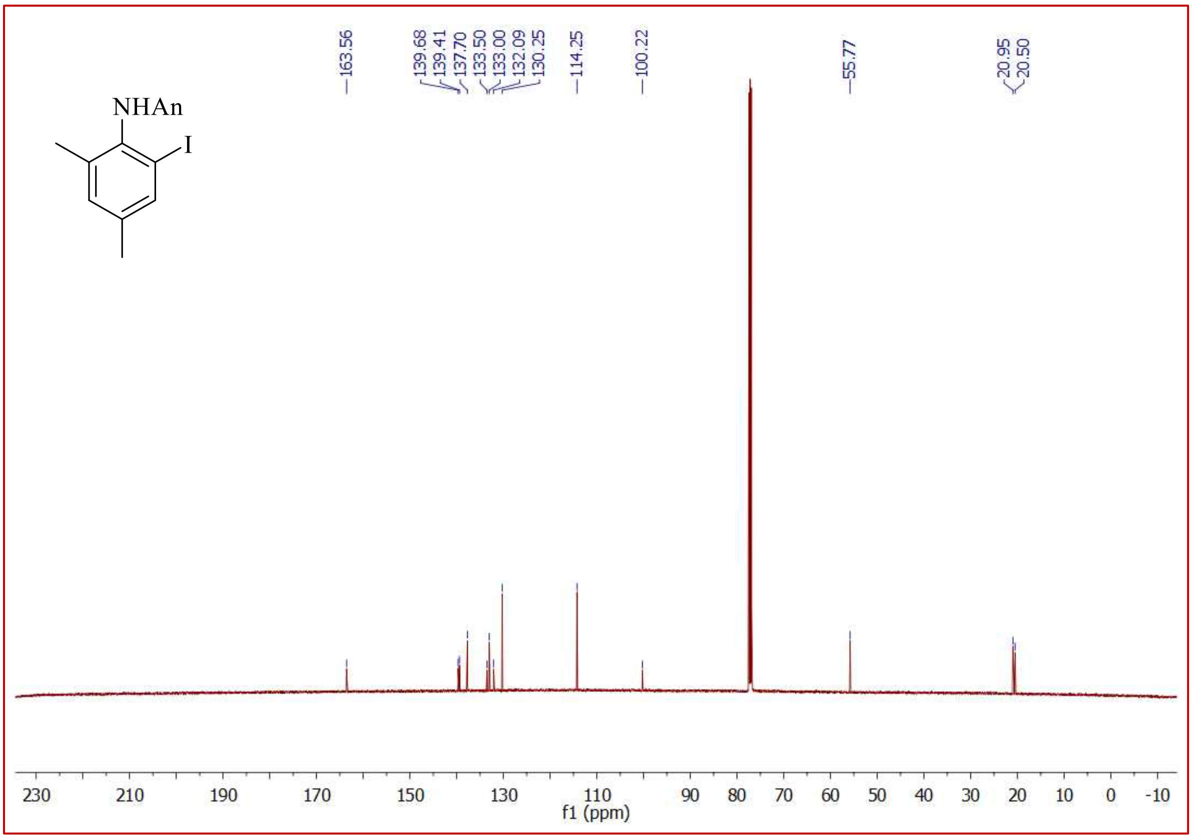

N-(4-Chloro-2-iodo-6-methylphenyl)-4-methylbenzenesulfonamide (**6d**), ^{1}H NMR

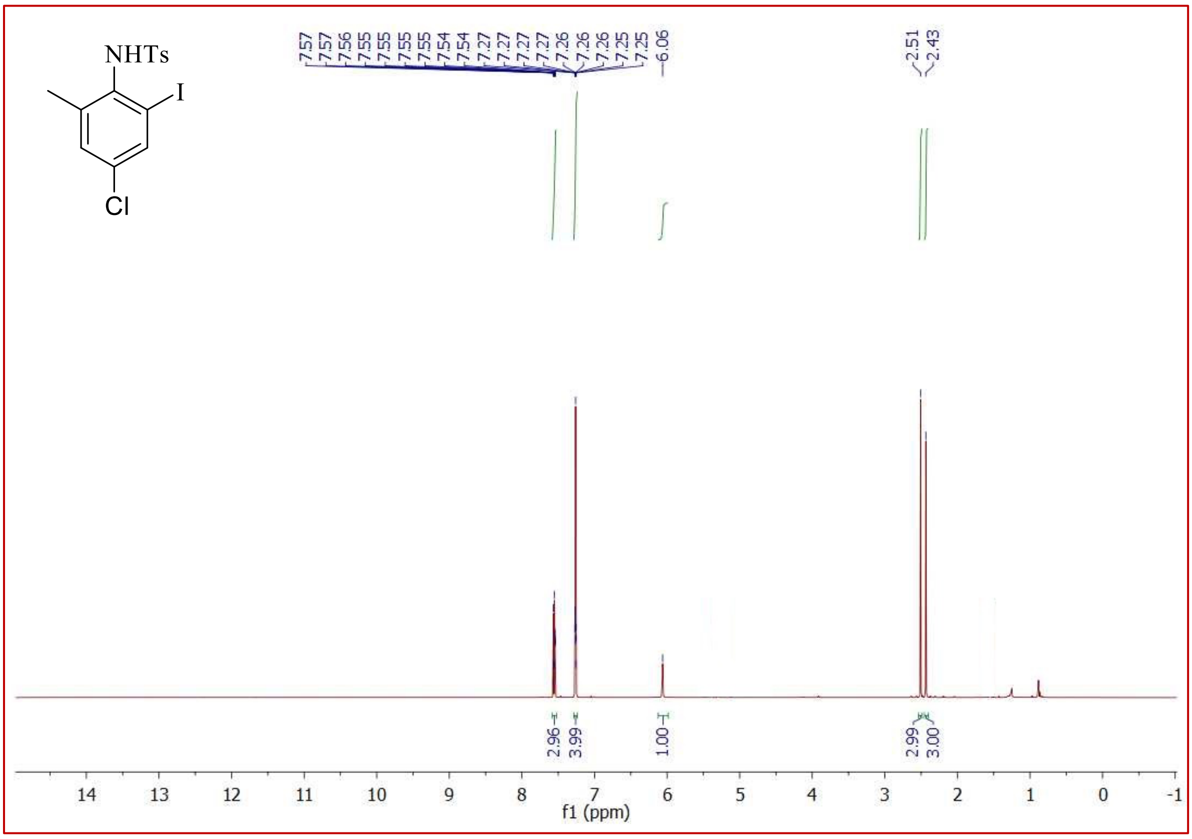

N-(4-Chloro-2-iodo-6-methylphenyl)-4-methylbenzenesulfonamide (**6d**), ^{13}C NMR

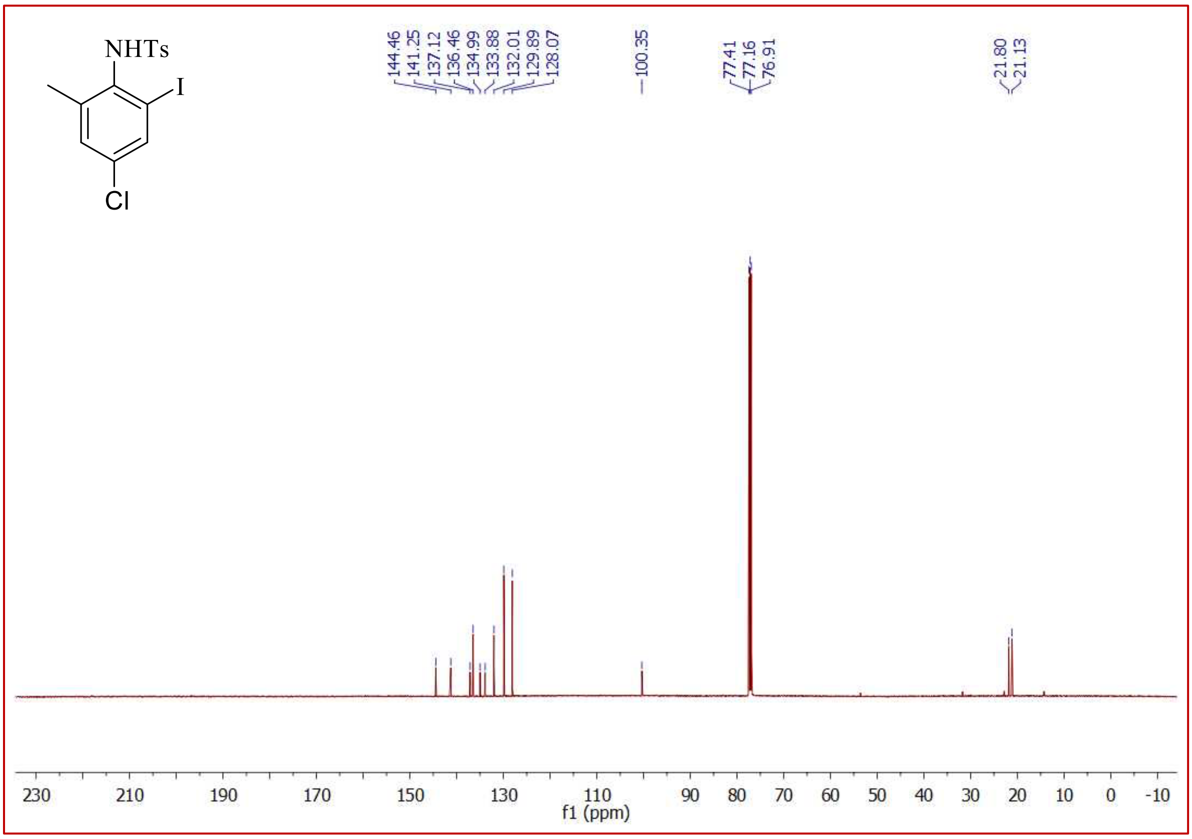

(*R, S*$_{N\text{-}C}$)-(Methyl *N*-(2-iodo-4,6-dimethylphenyl)-*N*-((4-nitrophenyl) sulfonyl)-alaninate (**7a**), ^{1}H NMR

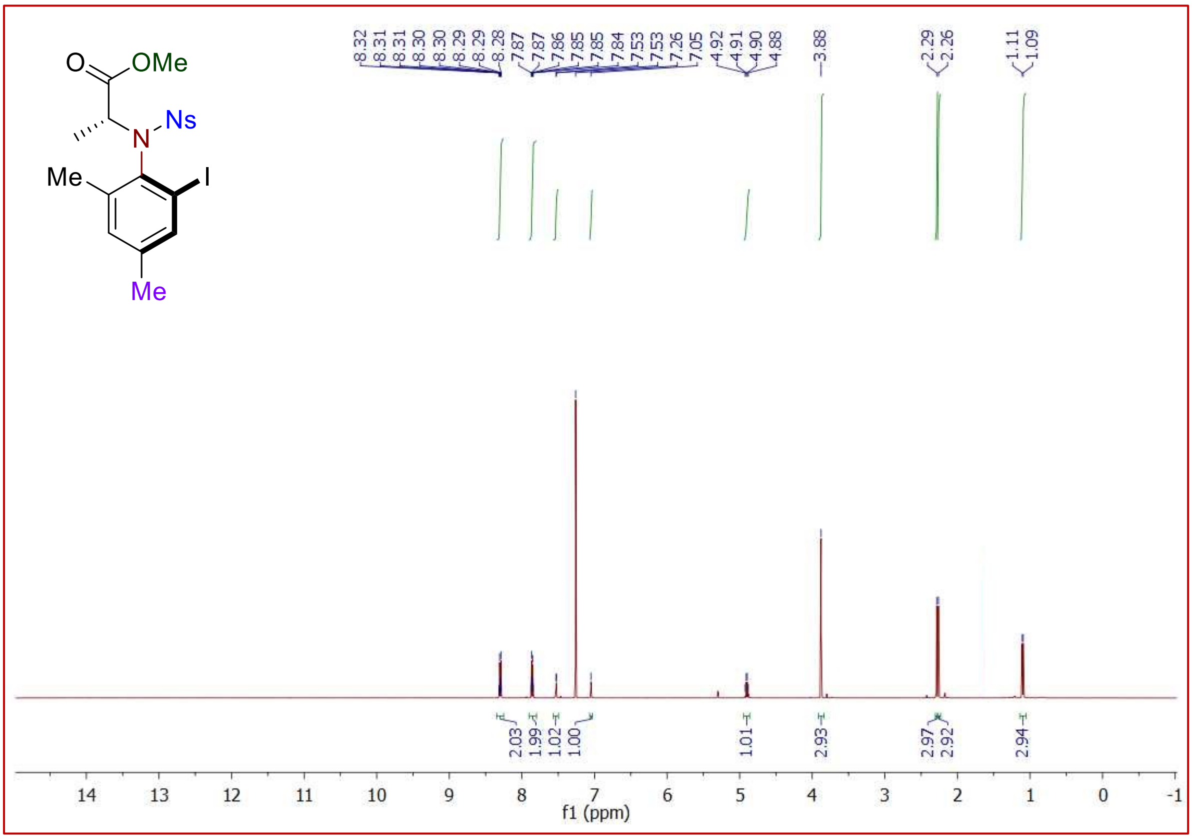

(*R, S*$_{N\text{-}C}$)-(Methyl *N*-(2-iodo-4,6-dimethylphenyl)-*N*-((4-nitrophenyl) sulfonyl)-alaninate (**7a**), ^{13}C NMR

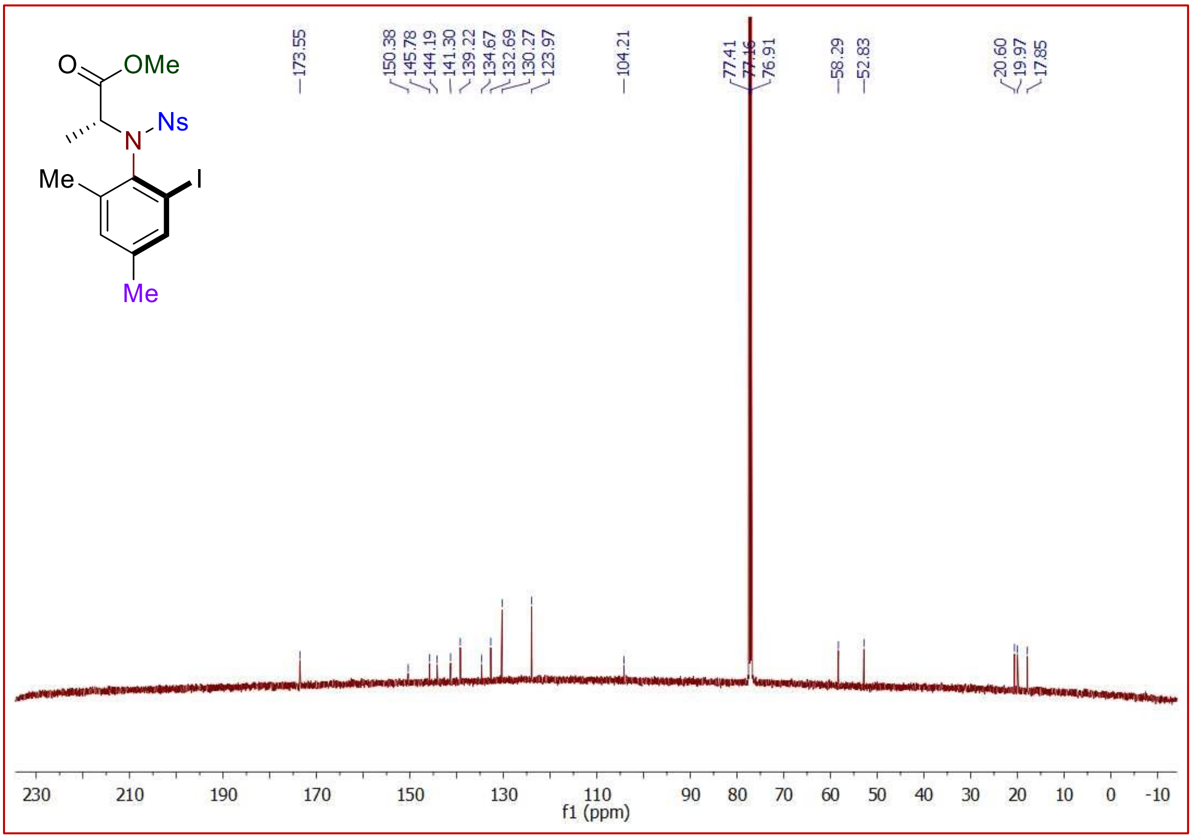

(R, $R_{N\text{-}C}$)-(Methyl *N*-(2-iodo-4,6-dimethylphenyl)-*N*-((4-nitrophenyl) sulfonyl)-alaninate (**7b**), ^{1}H NMR

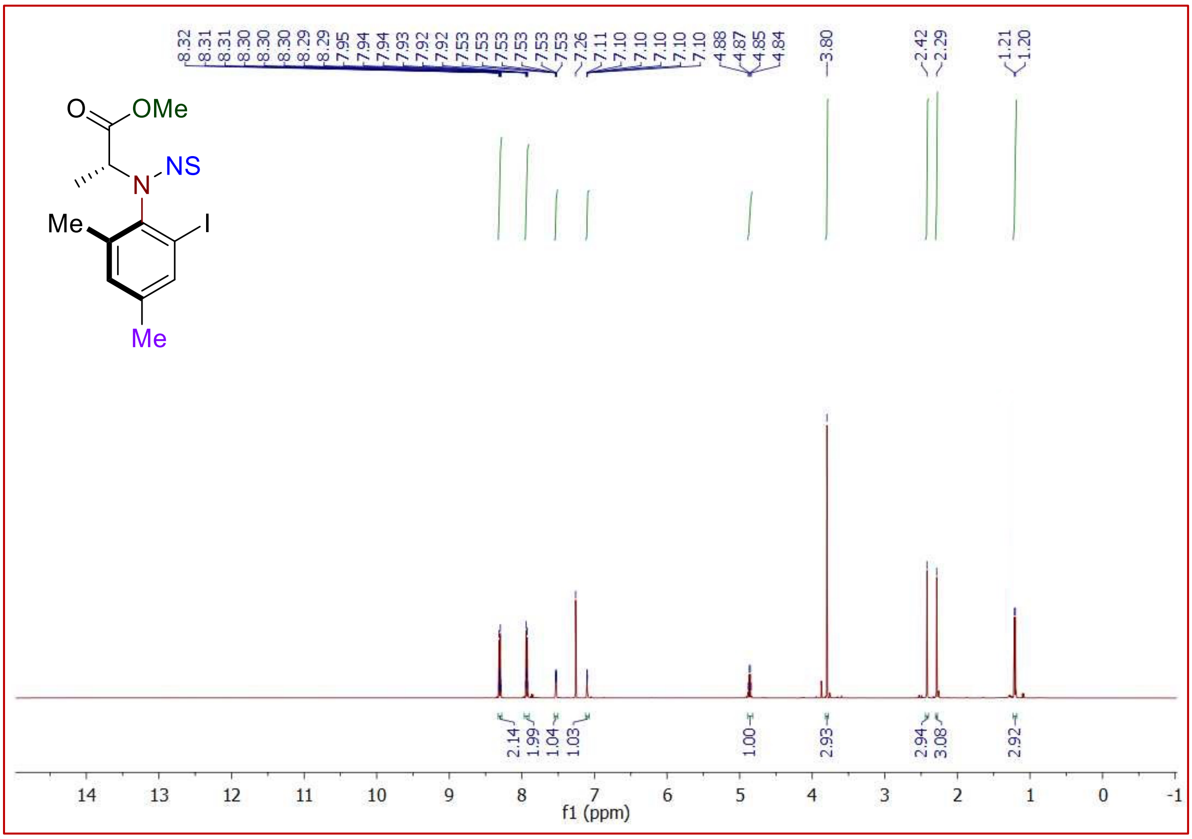

(R, $R_{N\text{-}C}$)-(Methyl *N*-(2-iodo-4,6-dimethylphenyl)-*N*-((4-nitrophenyl) sulfonyl)-alaninate (**7b**), ^{13}C NMR

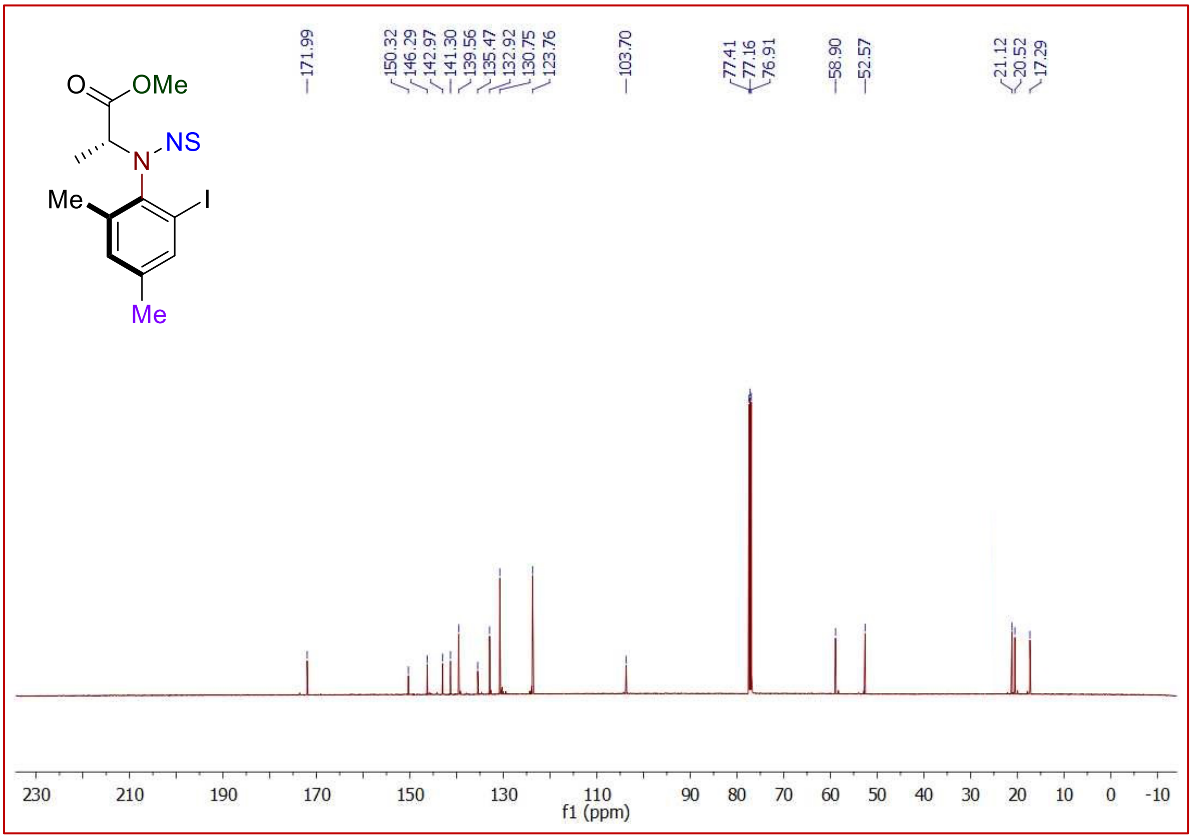

(*R, $S_{N\text{-}C}$*)-Methyl *N*-(2-iodo-4,6-dimethylphenyl)-*N*-tosyl-alaninate (**7c**), ^{1}H NMR

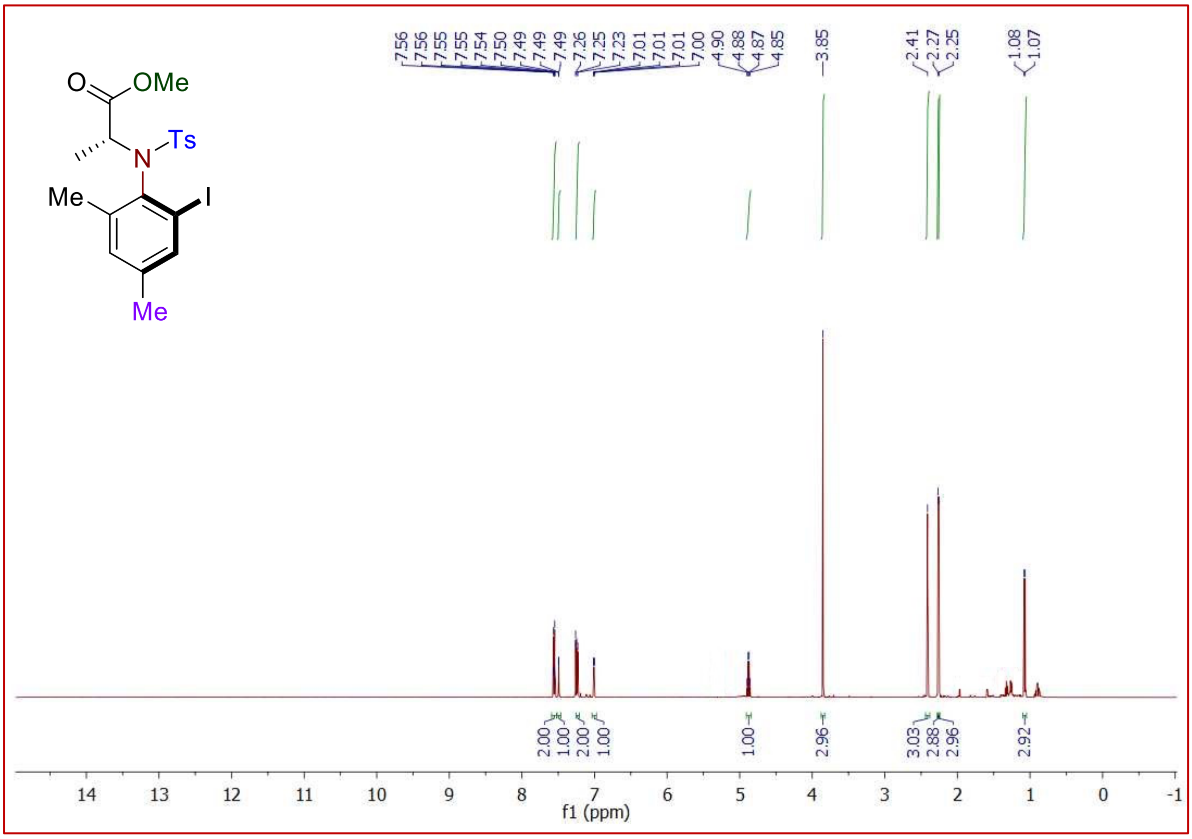

(*R, $S_{N\text{-}C}$*)-Methyl *N*-(2-iodo-4,6-dimethylphenyl)-*N*-tosyl-alaninate (**7c**), ^{13}C NMR

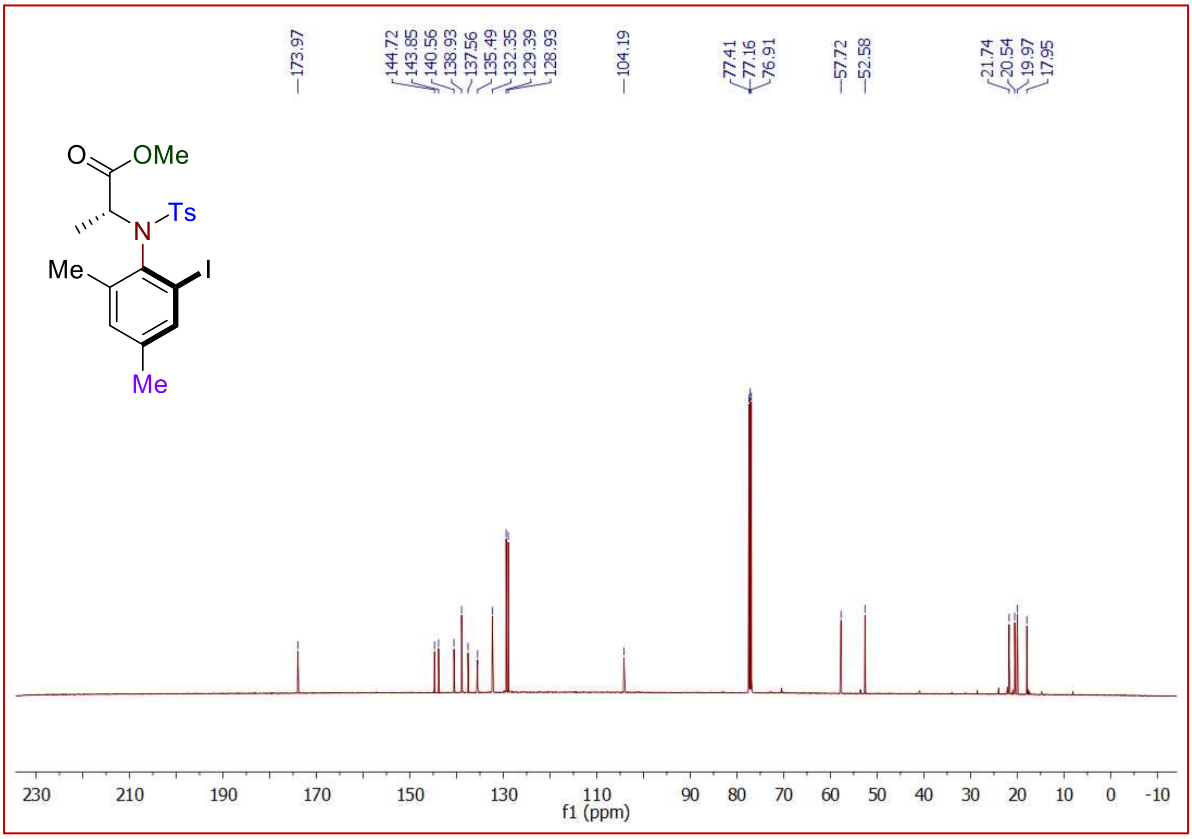

(*R, R*$_{N-C}$)-Methyl *N*-(2-iodo-4,6-dimethylphenyl)-*N*-tosyl-alaninate (**7d**), ^{1}H NMR

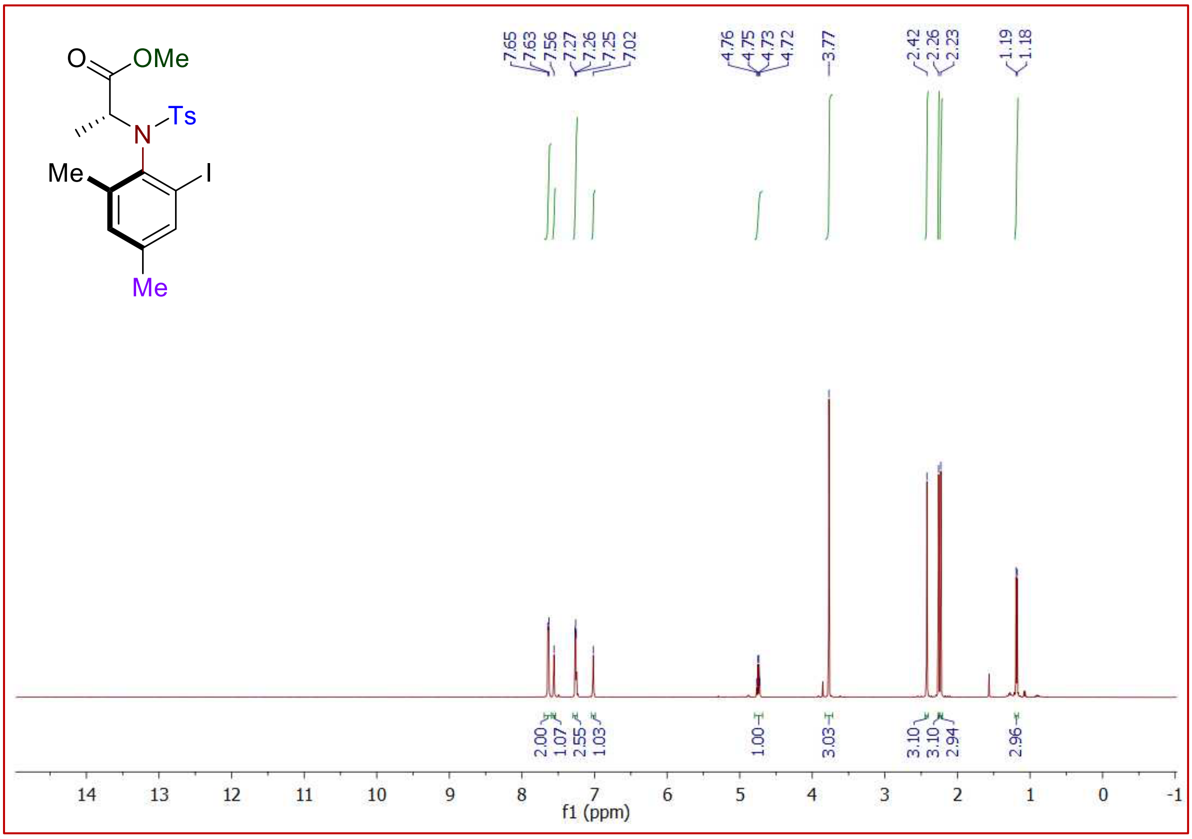

(*R, R*$_{N-C}$)-Methyl *N*-(2-iodo-4,6-dimethylphenyl)-*N*-tosyl-alaninate (**7d**), ^{13}C NMR

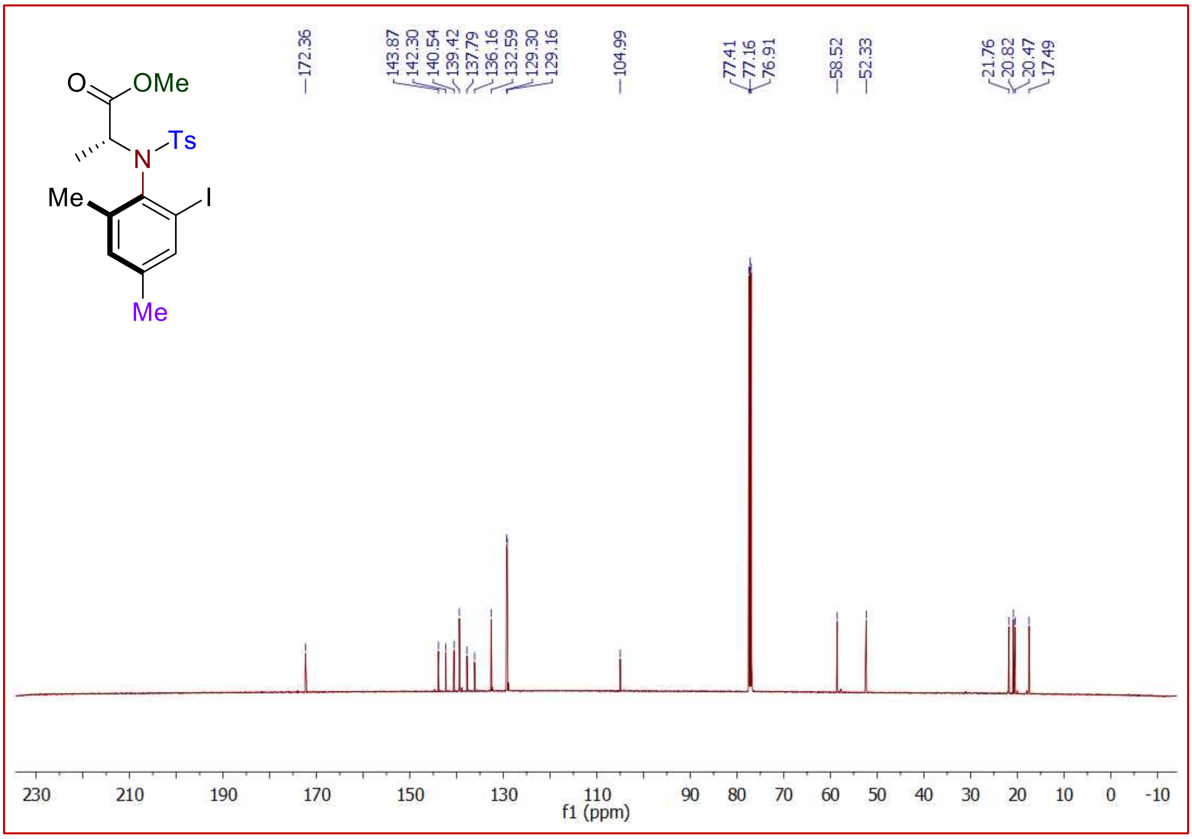

(*R*, $S_{N\text{-}C}$)-Methyl *N*-(2-iodo-4,6-dimethylphenyl)-*N*-((4-methoxyphenyl) sulfonyl)-D-alaninate (**7e**), ^{1}H NMR

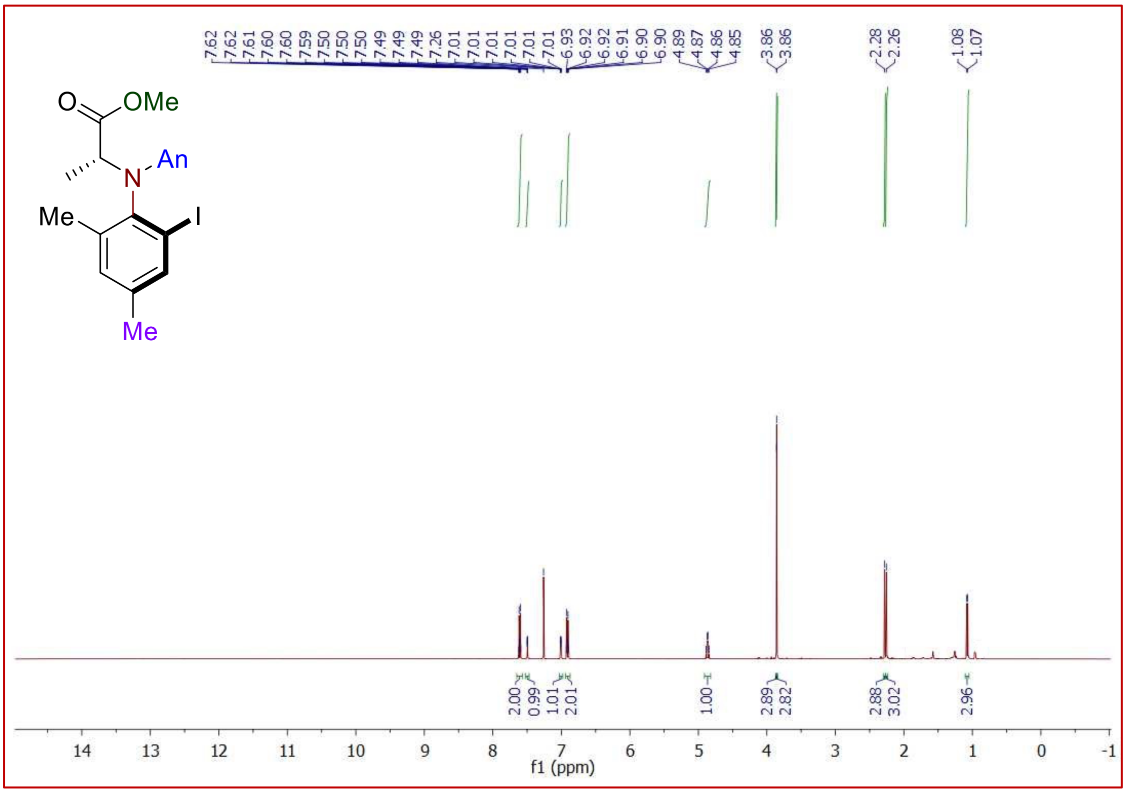

(*R*, $S_{N\text{-}C}$)-Methyl N-(2-iodo-4,6-dimethylphenyl)-*N*-((4-methoxyphenyl) sulfonyl)-D-alaninate (**7e**), ^{13}C NMR

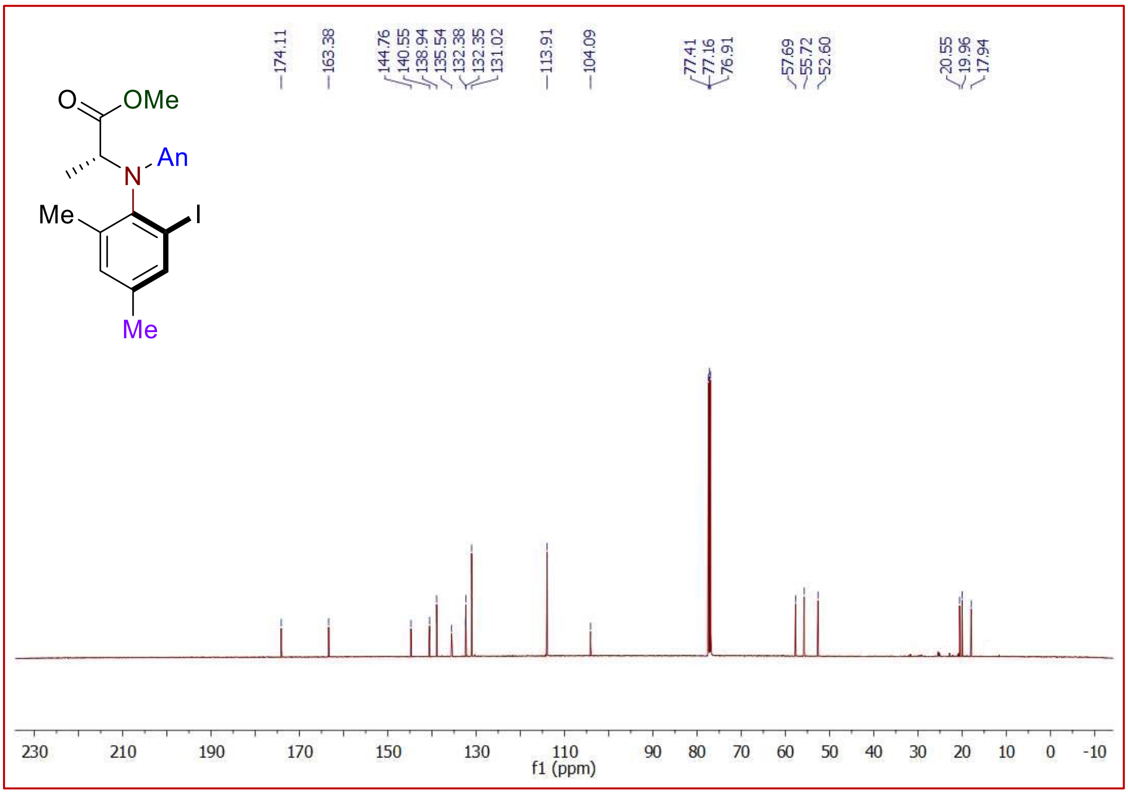

(*R*, R_{N-C})-Methyl *N*-(2-iodo-4,6-dimethylphenyl)-*N*-((4-methoxyphenyl) sulfonyl)-D-alaninate (**7f**), ^{1}H NMR

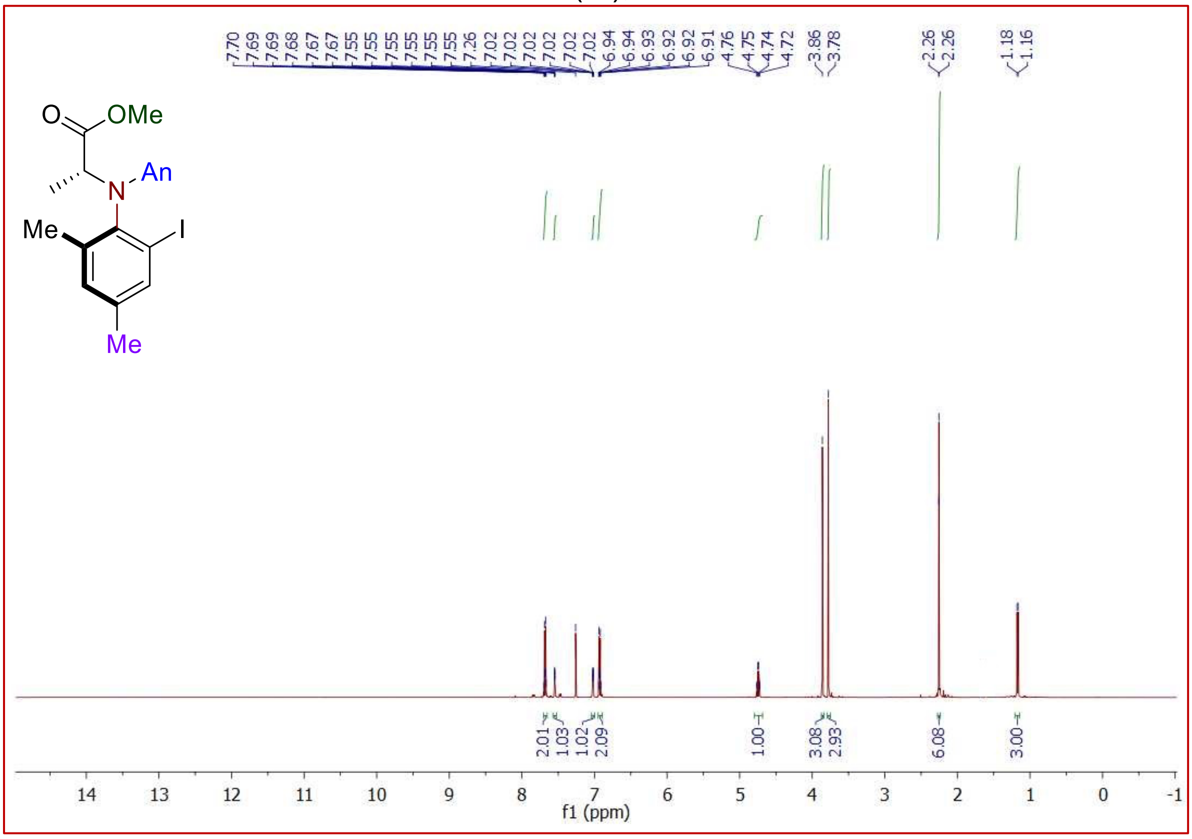

(*R*, R_{N-C})-Methyl *N*-(2-iodo-4,6-dimethylphenyl)-*N*-((4-methoxyphenyl) sulfonyl)-D-alaninate (**7f**), ^{13}C NMR

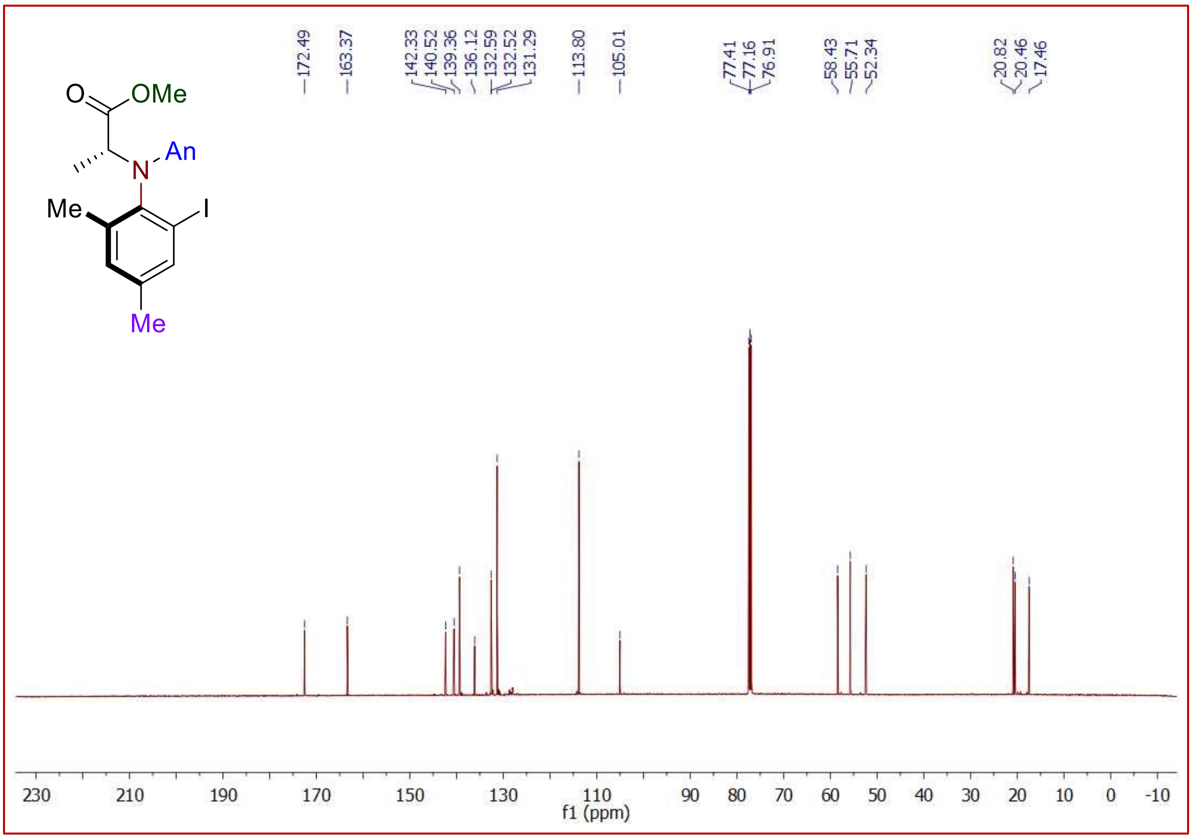

(*R, S*$_{N-C}$)-Methyl *N*-(4-chloro-2-iodo-6-methylphenyl)-*N*-tosyl-L-alaninate (**7g**), ^{1}H NMR

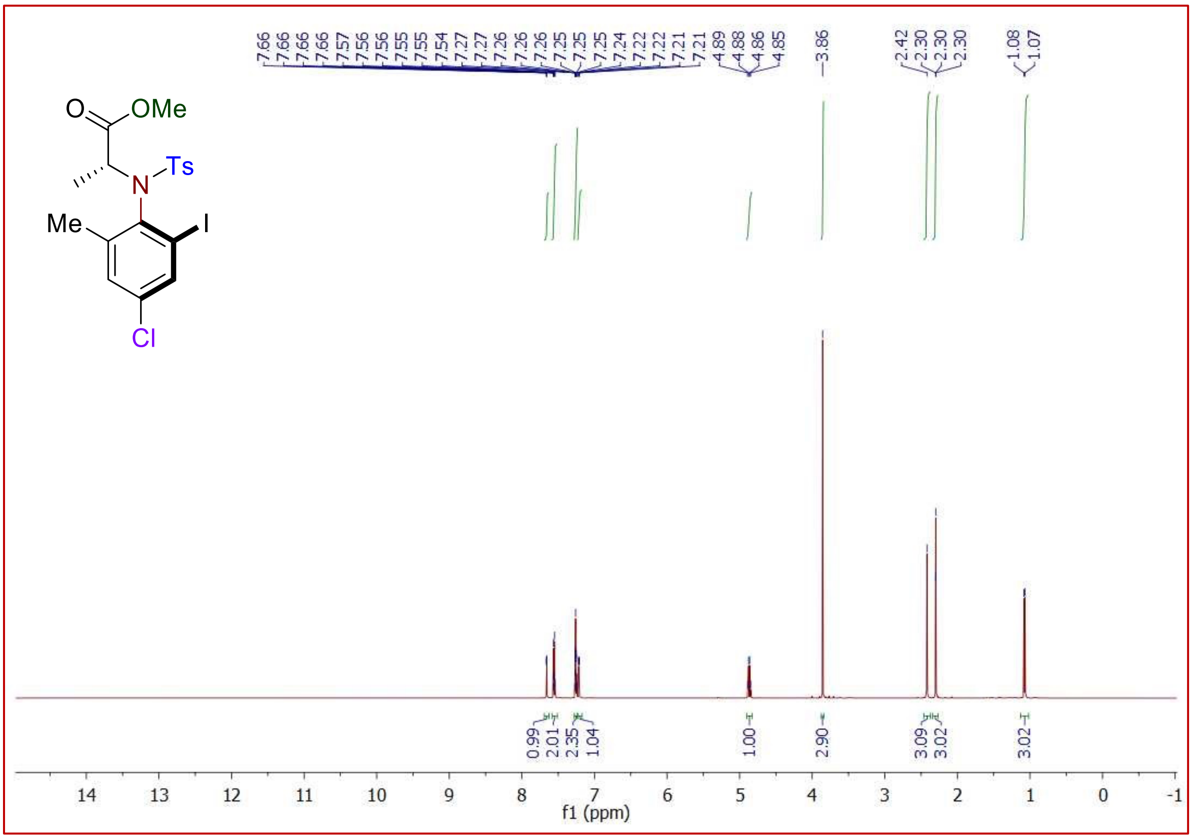

(*R, S*$_{N-C}$)-Methyl *N*-(4-chloro-2-iodo-6-methylphenyl)-*N*-tosyl-L-alaninate (**7g**), ^{13}C NMR

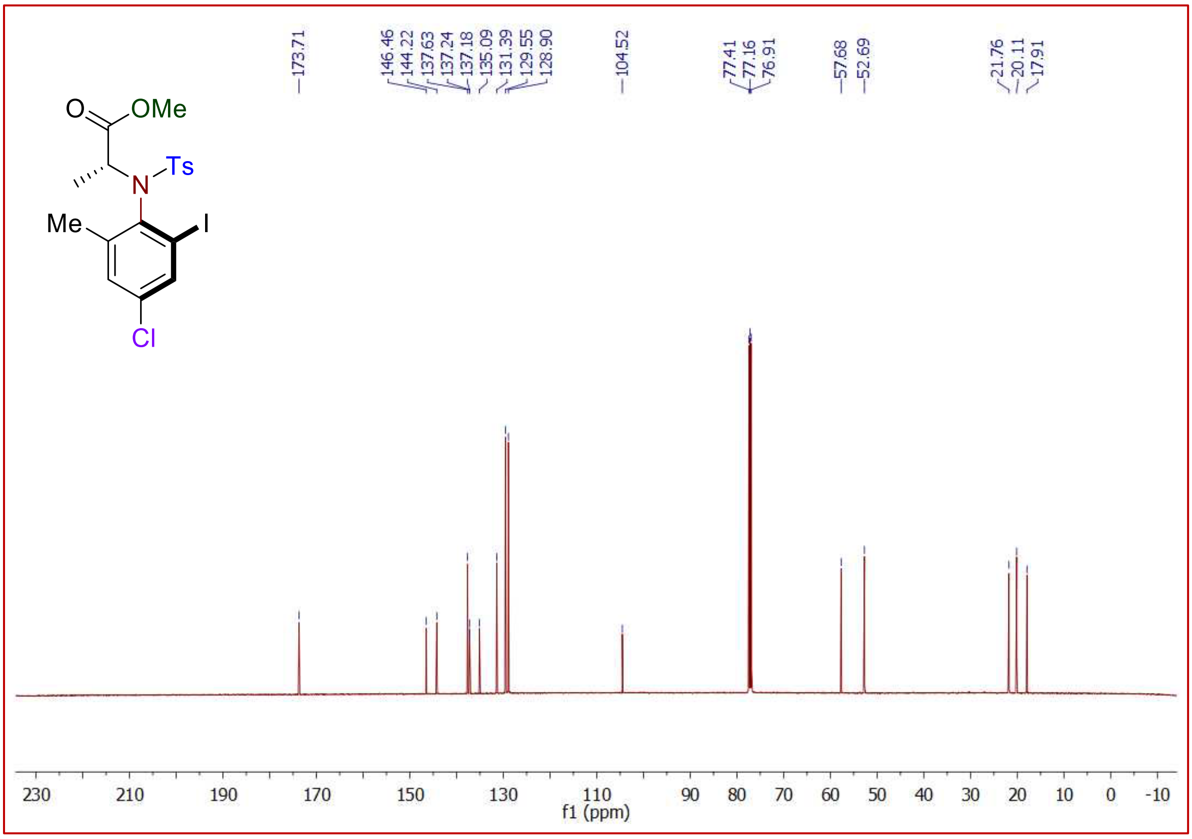

(*R, R$_{N\text{-}C}$*)-Methyl *N*-(4-chloro-2-iodo-6-methylphenyl)-*N*-tosyl-L-alaninate (**7h**), ^{1}H NMR

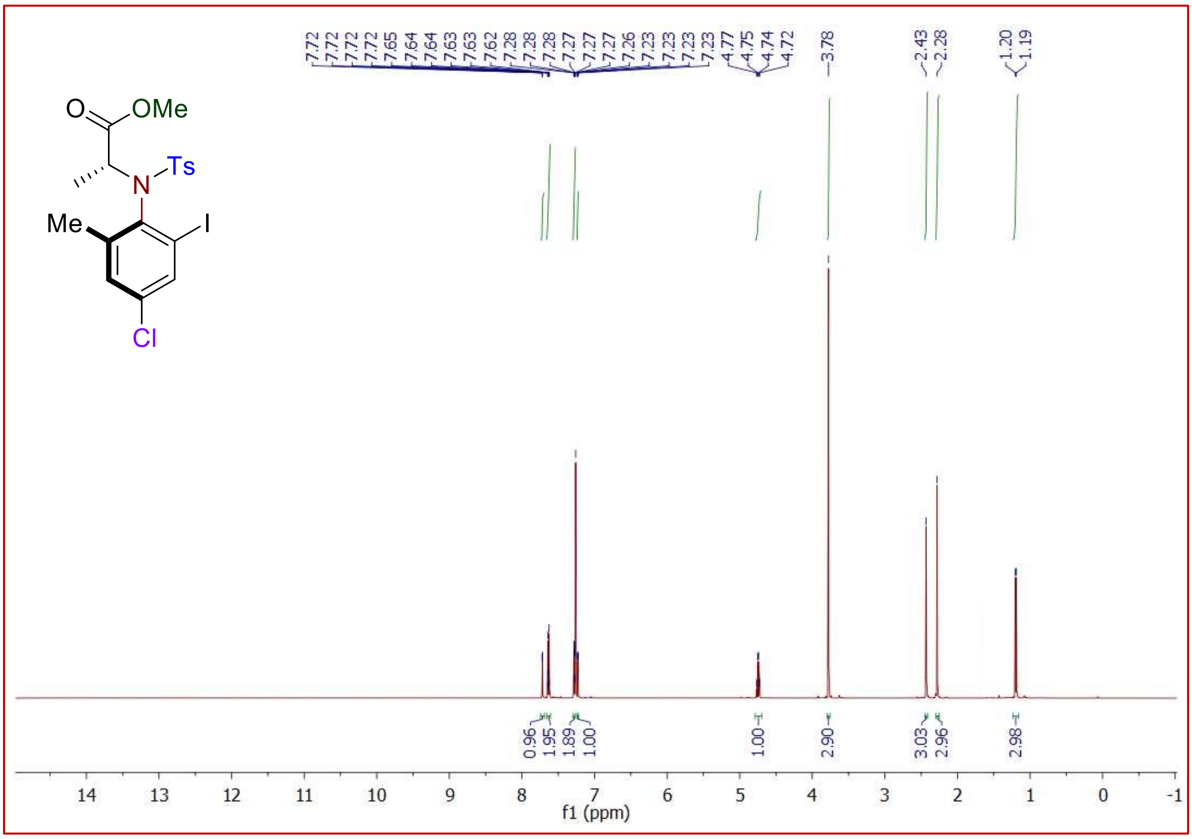

(*R, R$_{N\text{-}C}$*)-Methyl *N*-(4-chloro-2-iodo-6-methylphenyl)-*N*-tosyl-L-alaninate (**7h**), ^{13}C NMR

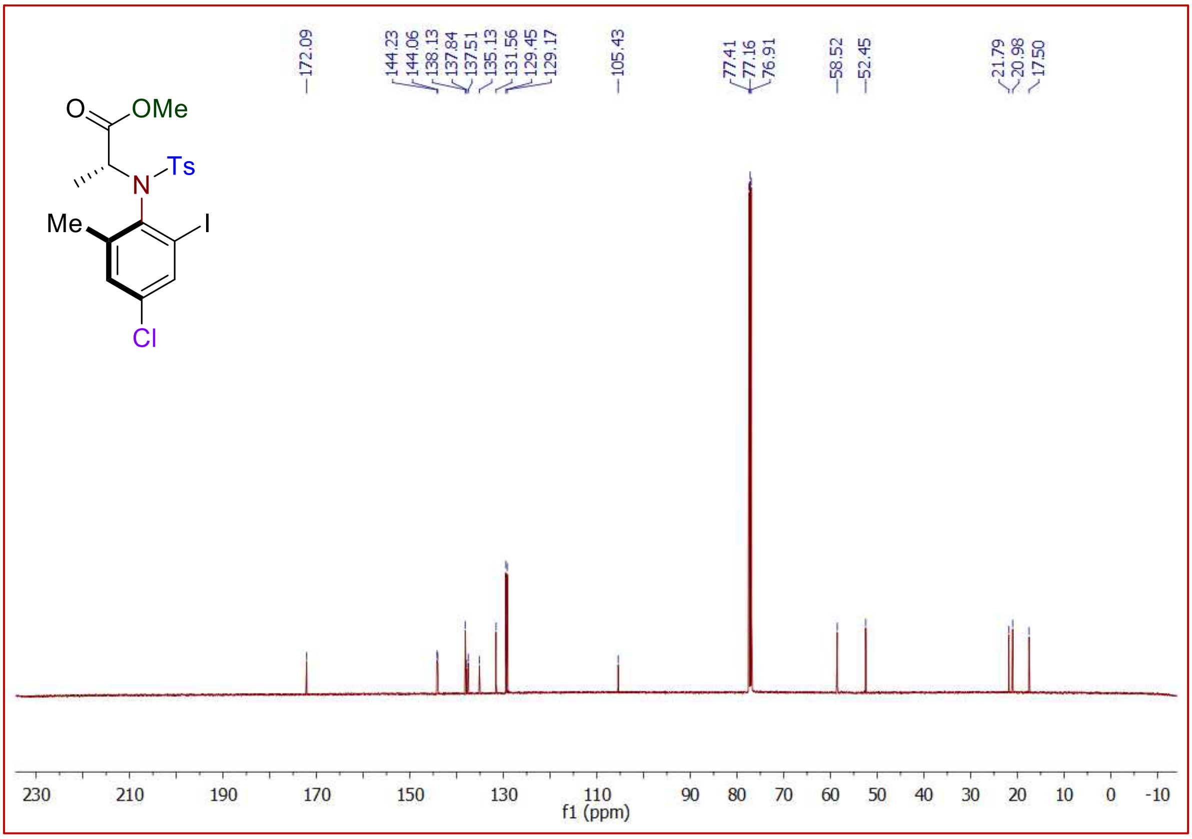

(*R, S*$_{N\text{-}C}$)-(Benzyl *N*-(2-iodo-4,6-dimethylphenyl)-*N*-((4-nitrophenyl) sulfonyl)-alaninate (**7i**), ^{1}H NMR

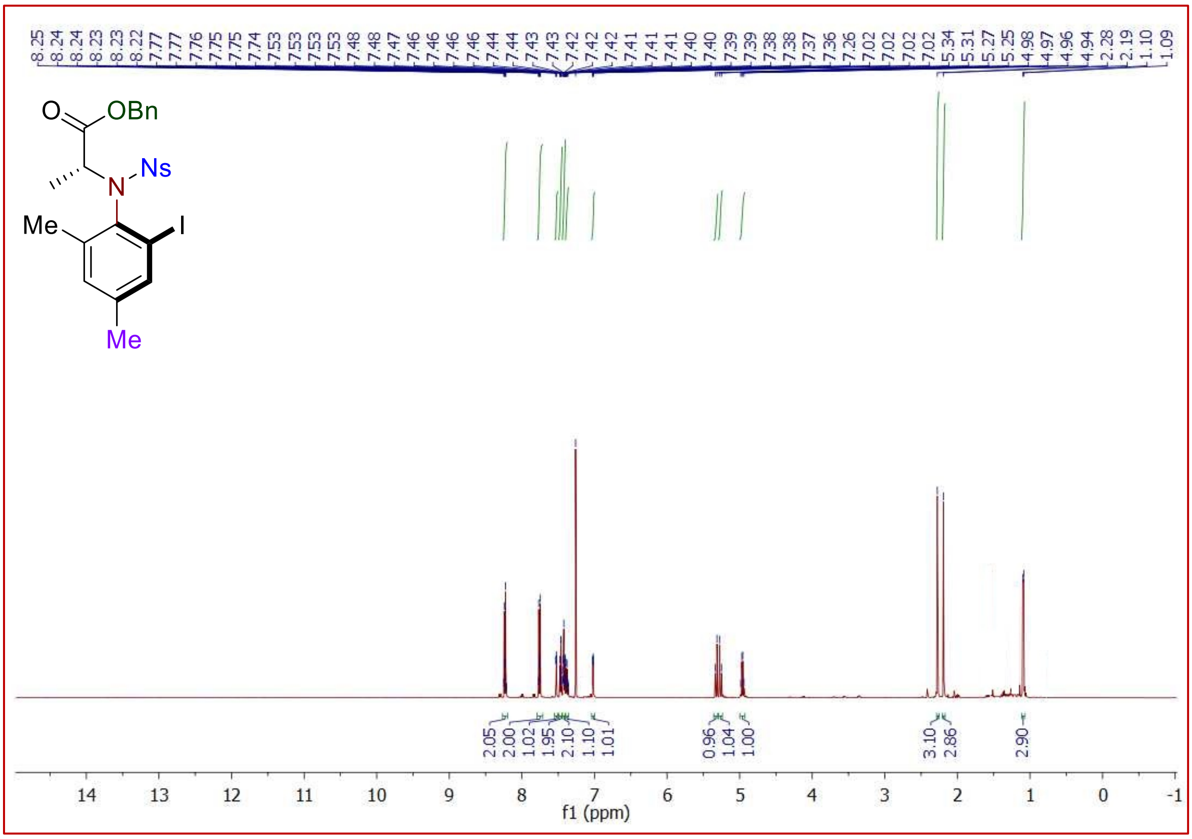

(*R, S*$_{N\text{-}C}$)-(Benzyl *N*-(2-iodo-4,6-dimethylphenyl)-*N*-((4-nitrophenyl) sulfonyl)-alaninate (**7i**), ^{13}C NMR

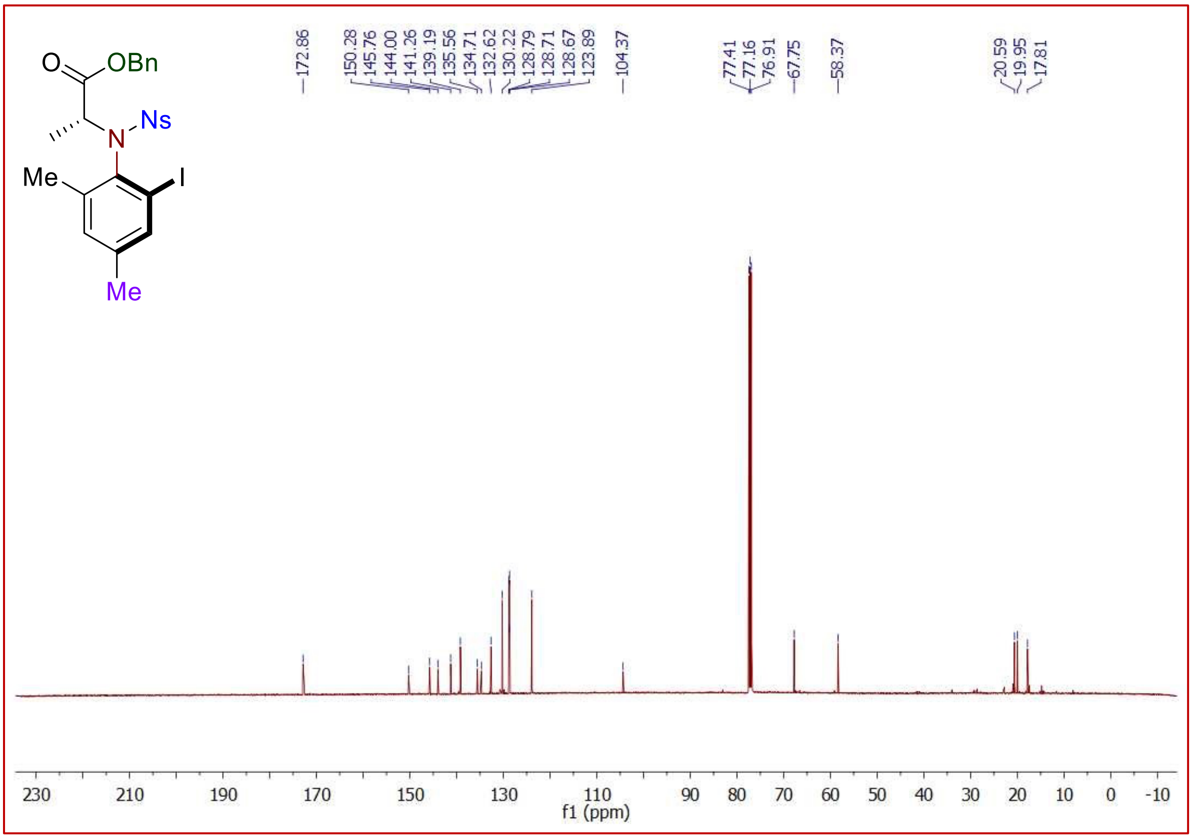

(*R*, *R*$_{N\text{-}C}$)-(Benzyl *N*-(2-iodo-4,6-dimethylphenyl)-*N*-((4-nitrophenyl) sulfonyl)-alaninate (**7j**), ^{1}H NMR

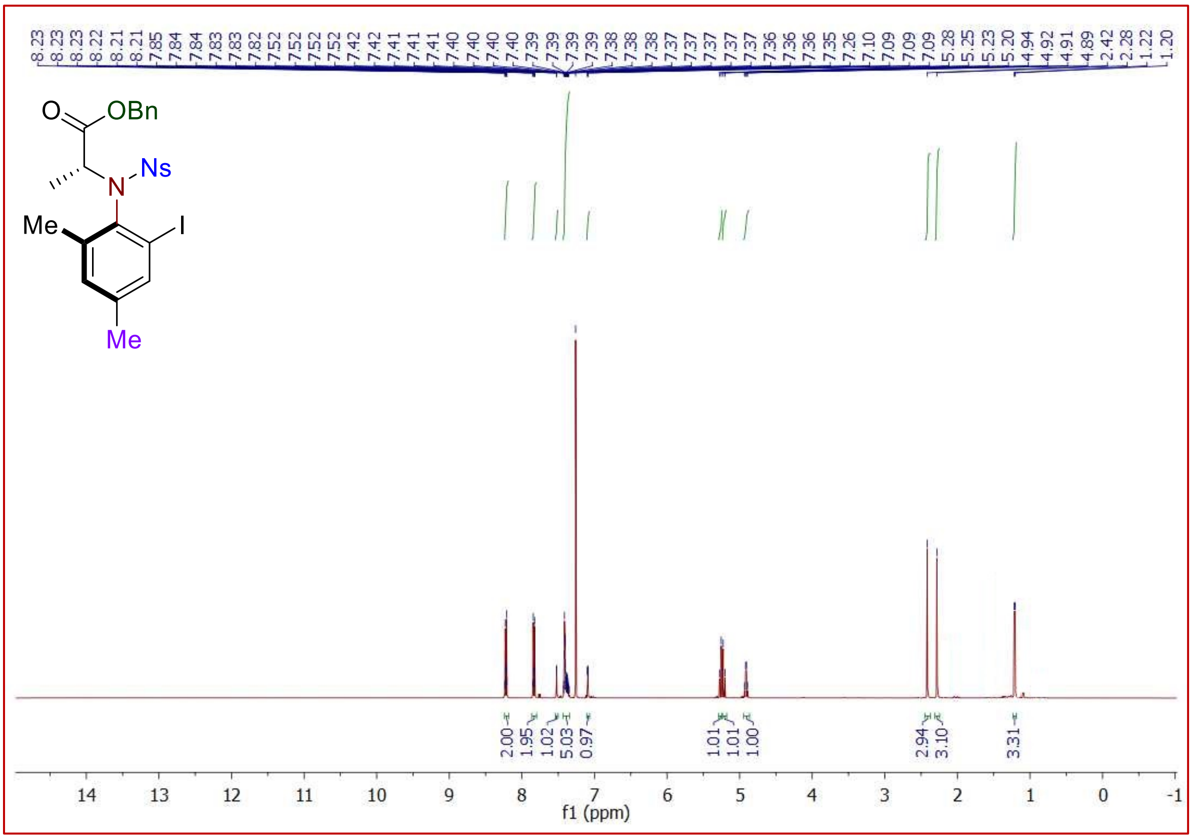

(*R*, *R*$_{N\text{-}C}$)-(Benzyl *N*-(2-iodo-4,6-dimethylphenyl)-*N*-((4-nitrophenyl) sulfonyl)-alaninate (**7j**), ^{13}C NMR

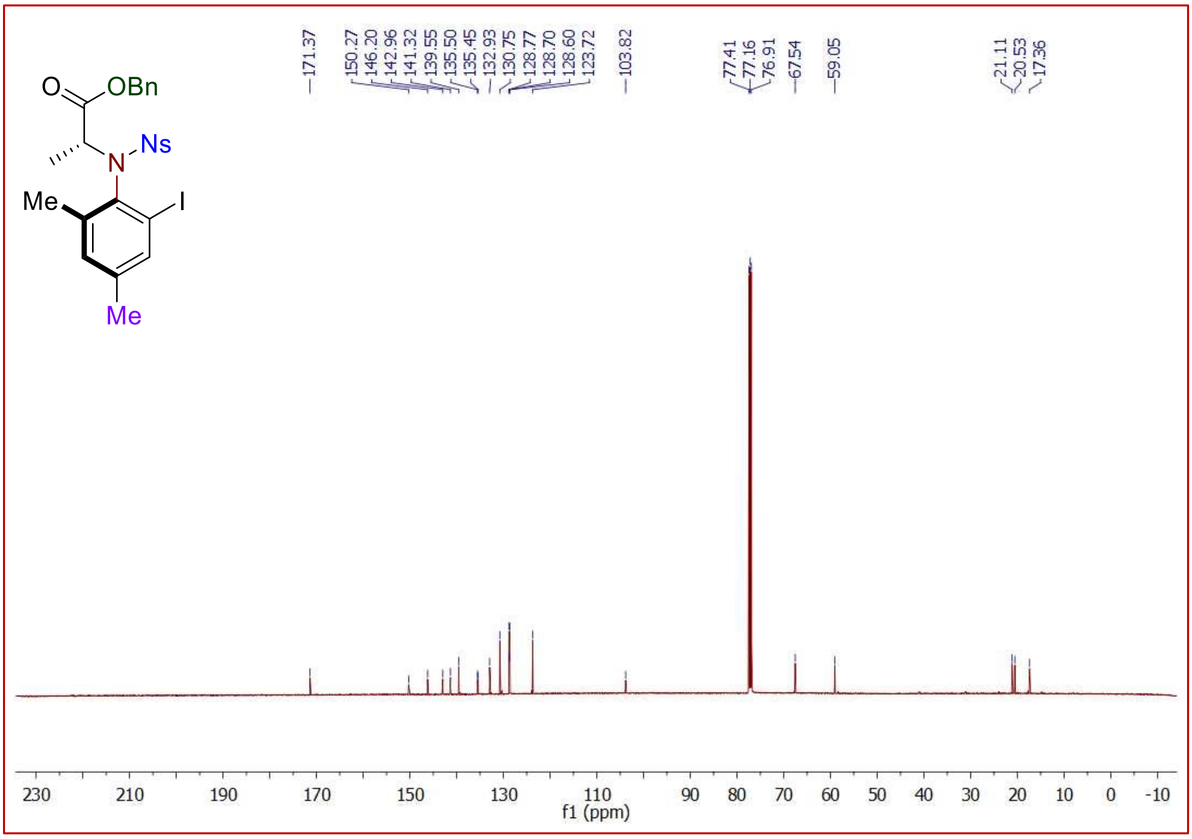

1-Oxo-1-phenylpropan-2-yl 4-methylbenzenesulfonate (**9a**), ^{1}H NMR

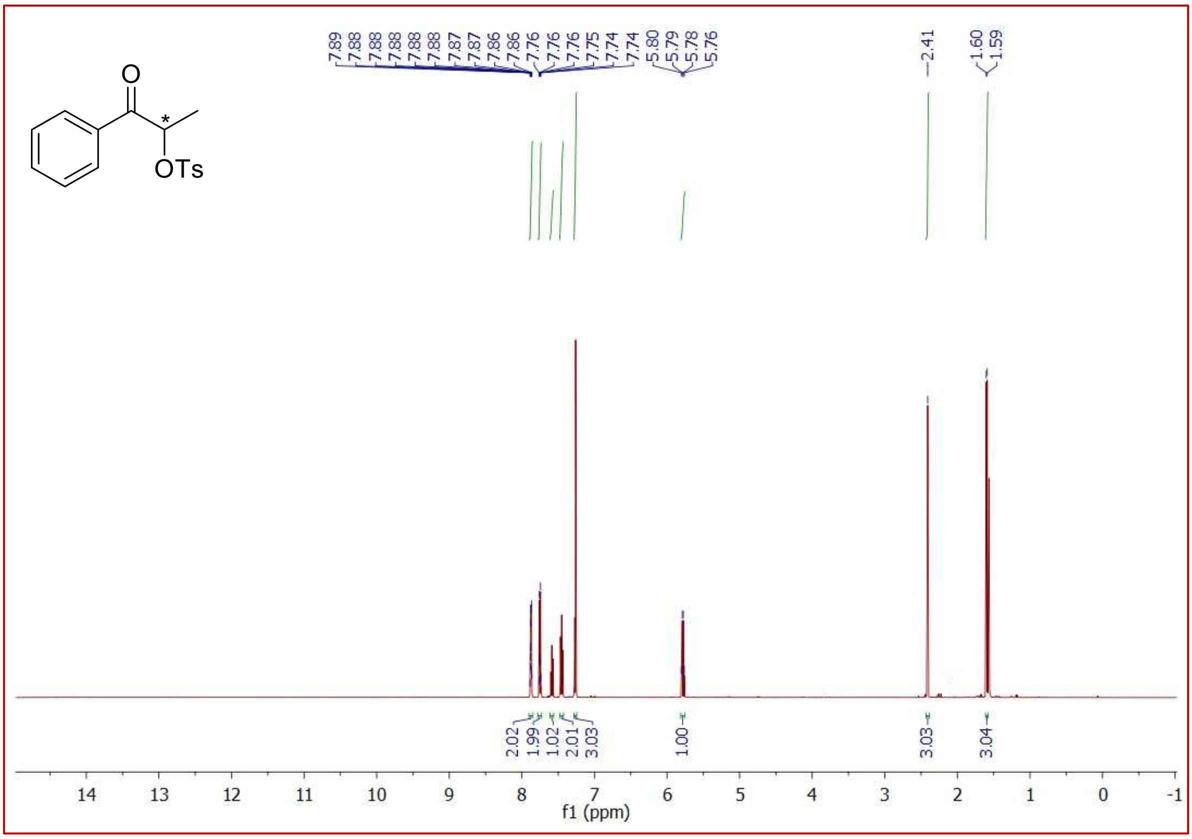

1-Oxo-1-phenylpropan-2-yl 4-methylbenzenesulfonate (**9a**), ^{13}C NMR

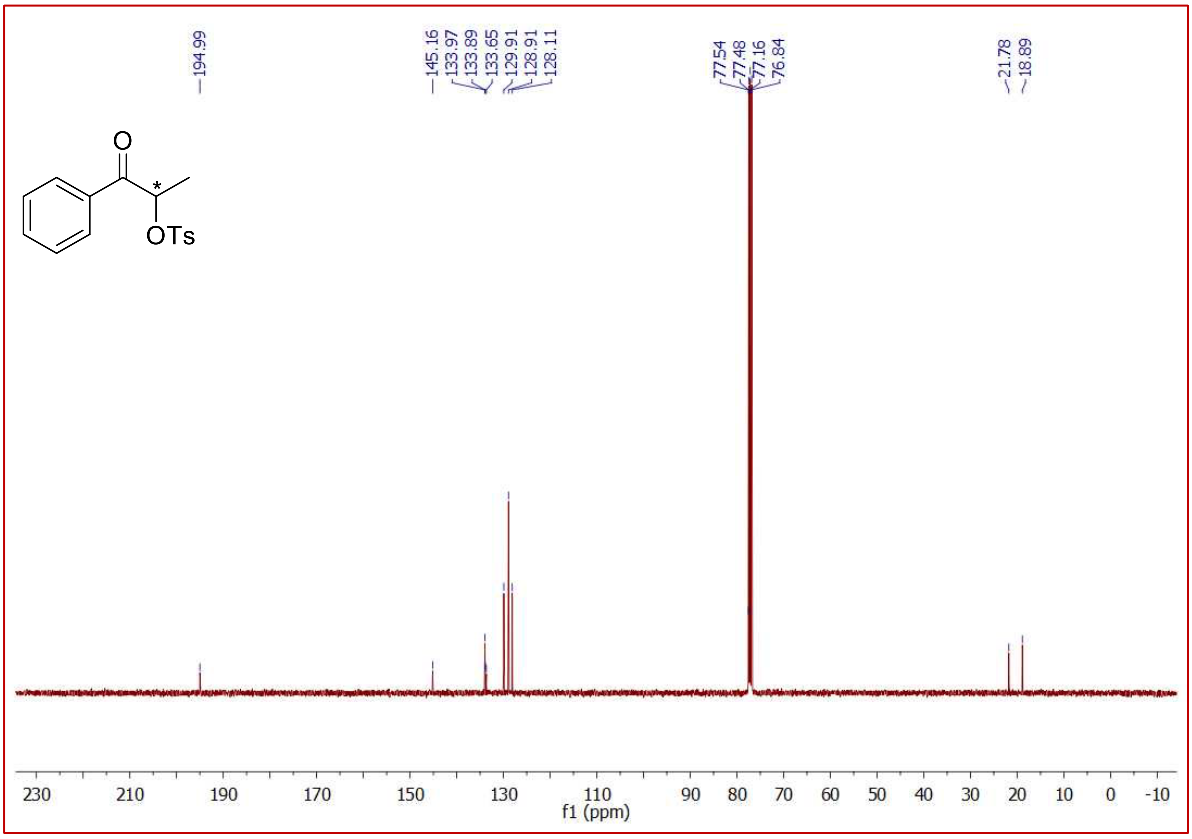

1-(4-Chlorophenyl)-1-oxopropan-2yl 4-methylbenzenesulfonate (**9b**), ^{1}H NMR

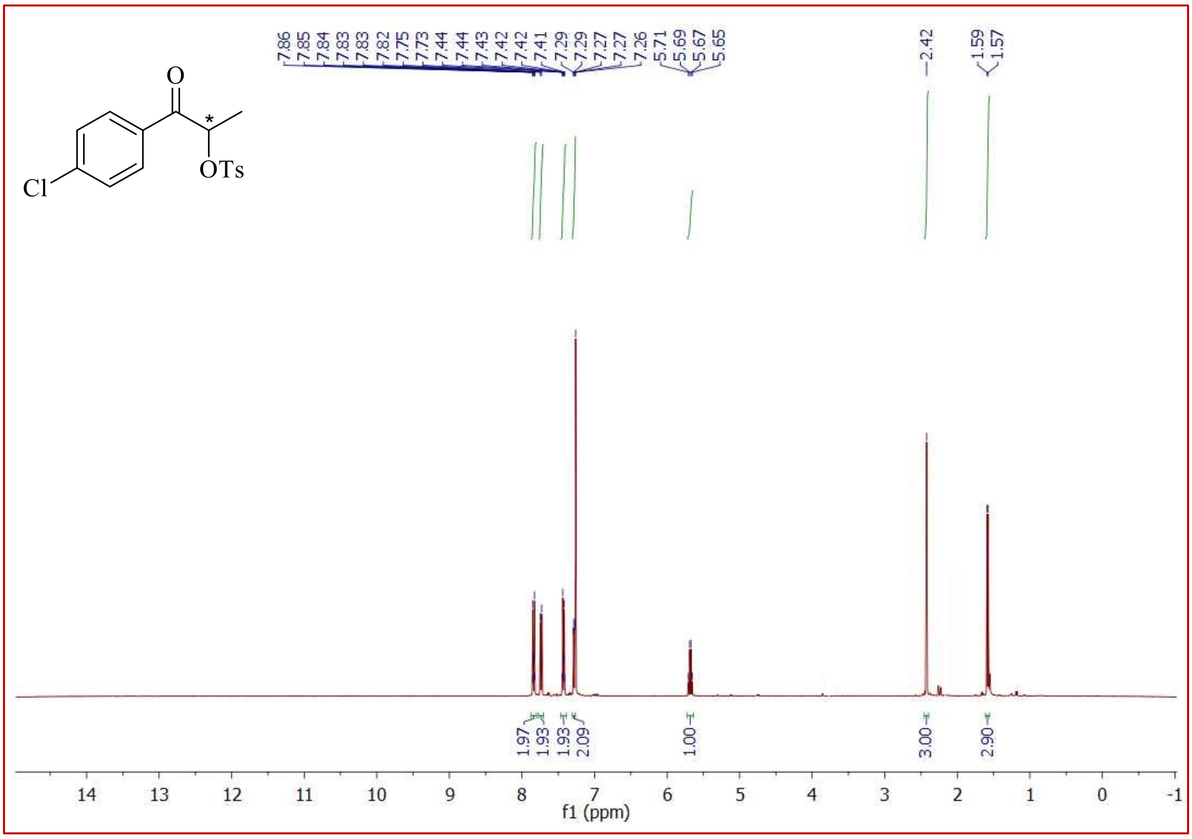

1-(4-Chlorophenyl)-1-oxopropan-2yl 4-methylbenzenesulfonate (**9b**), ^{13}C NMR

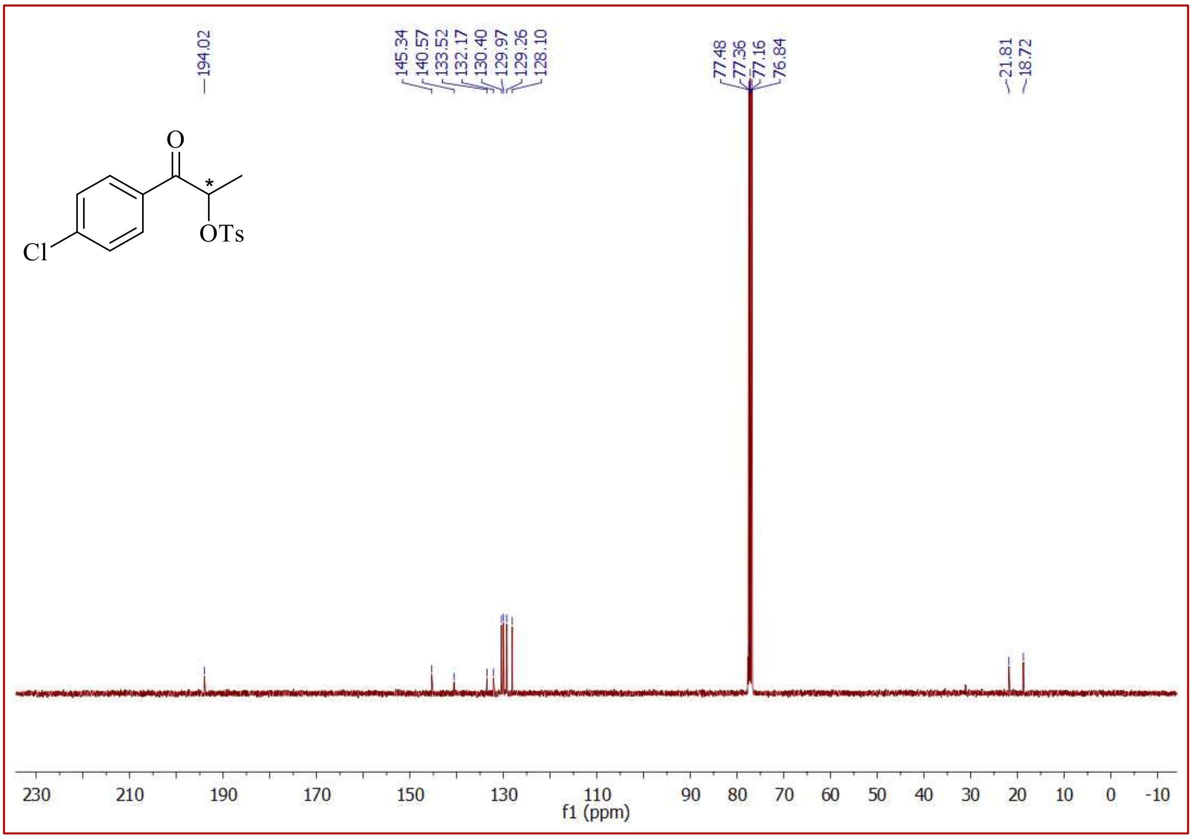

1-(3-Chlorophenyl)-1-oxopropan-2-yl 4-methylbenzenesulfonate (**9c**), ^{1}H NMR

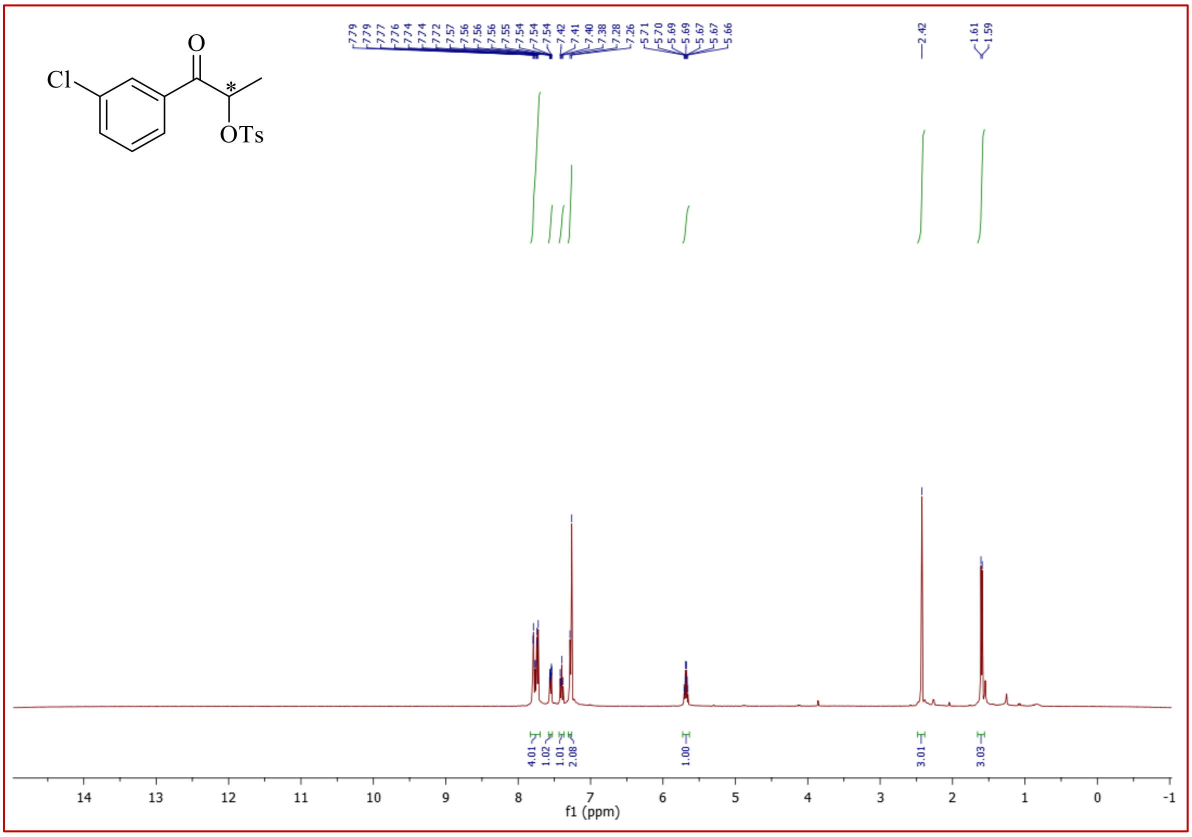

1-(3-Chlorophenyl)-1-oxopropan-2-yl 4-methylbenzenesulfonate (**9c**), ^{13}C NMR

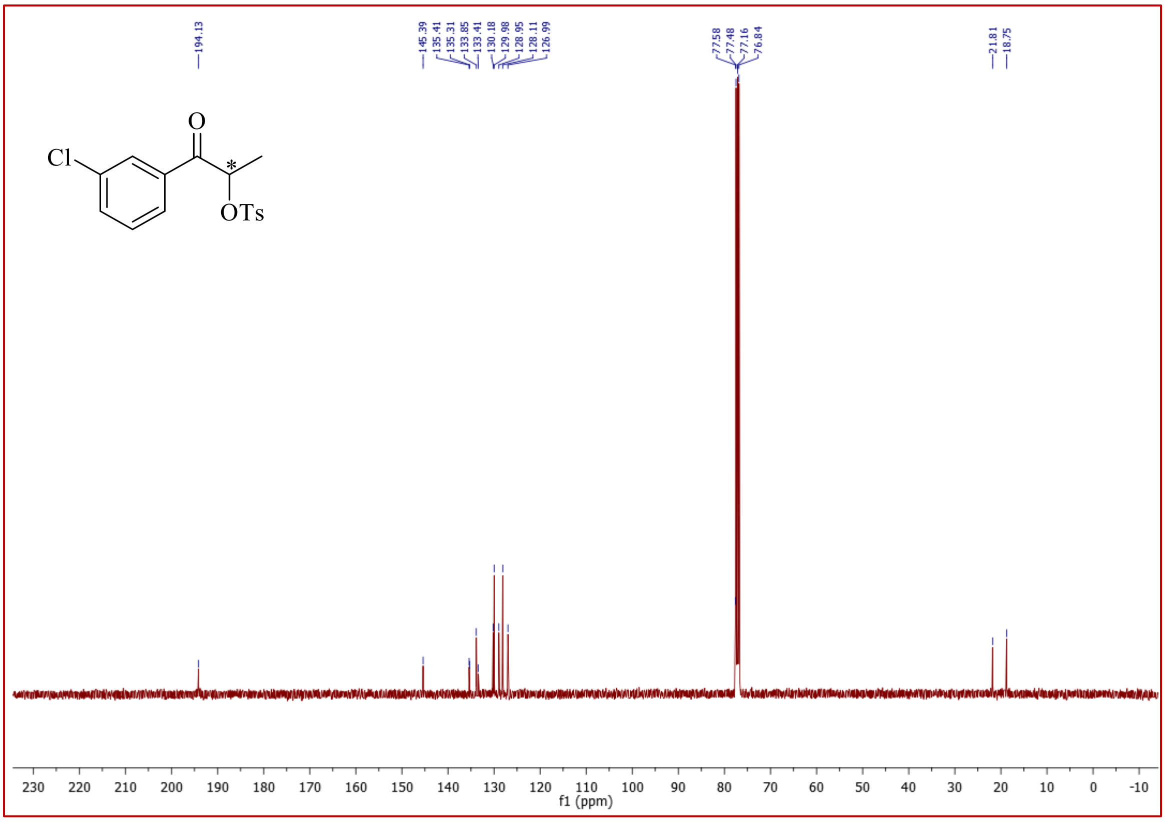

1-Oxo-1-(3-(trifluoromethyl) phenyl) propan-2-yl 4-methylbenzenesulfonate (**9d**), ^{1}H NMR

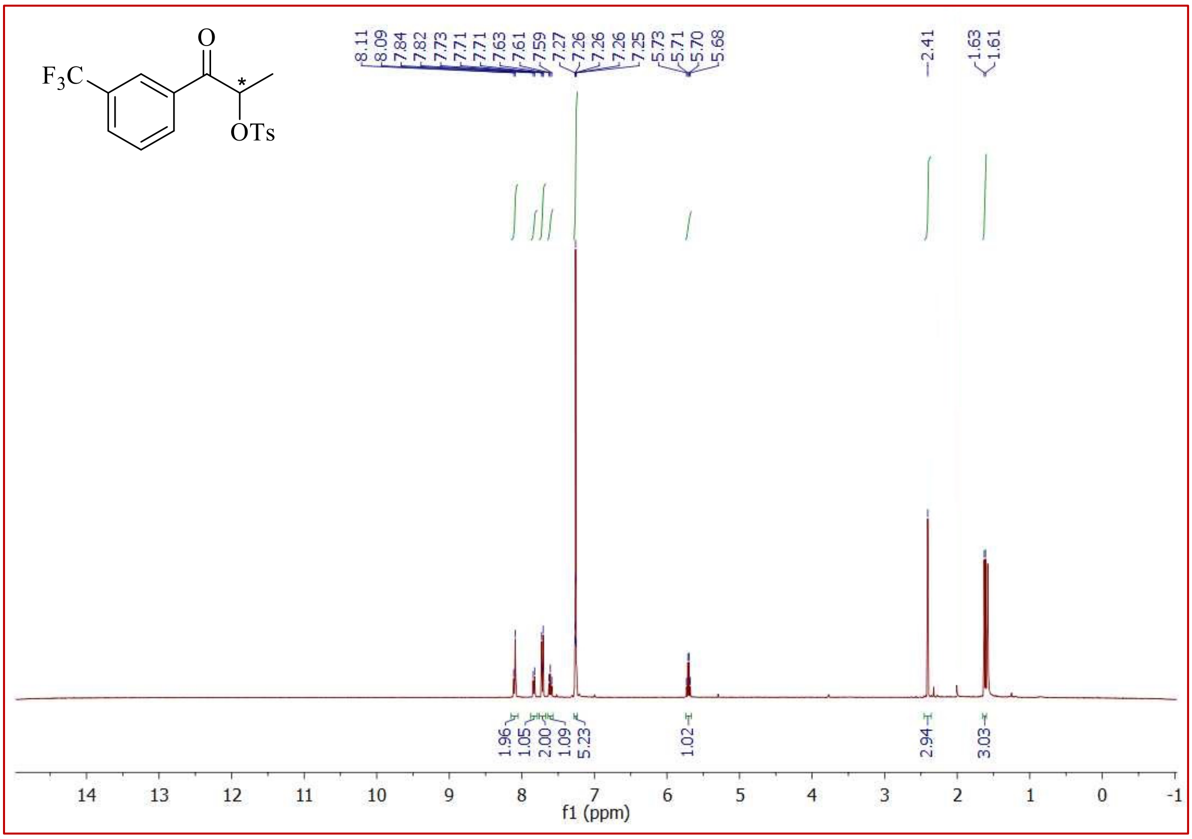

1-Oxo-1-(3-(trifluoromethyl) phenyl) propan-2-yl 4-methylbenzenesulfonate (**9d**), ^{13}C NMR

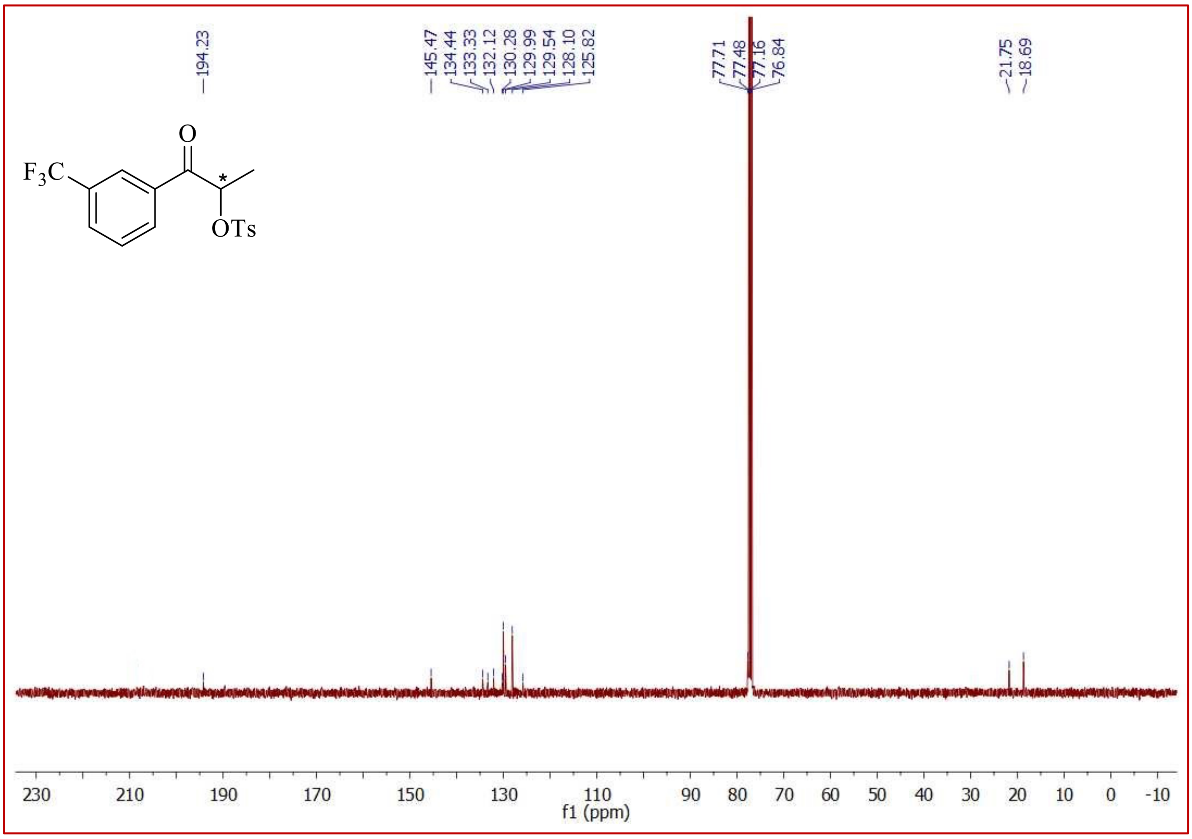

1-(3-Nitrophenyl)-1-oxopropan-2-yl 4-methylbenzenesulfonate (**9e**), ^{1}H NMR

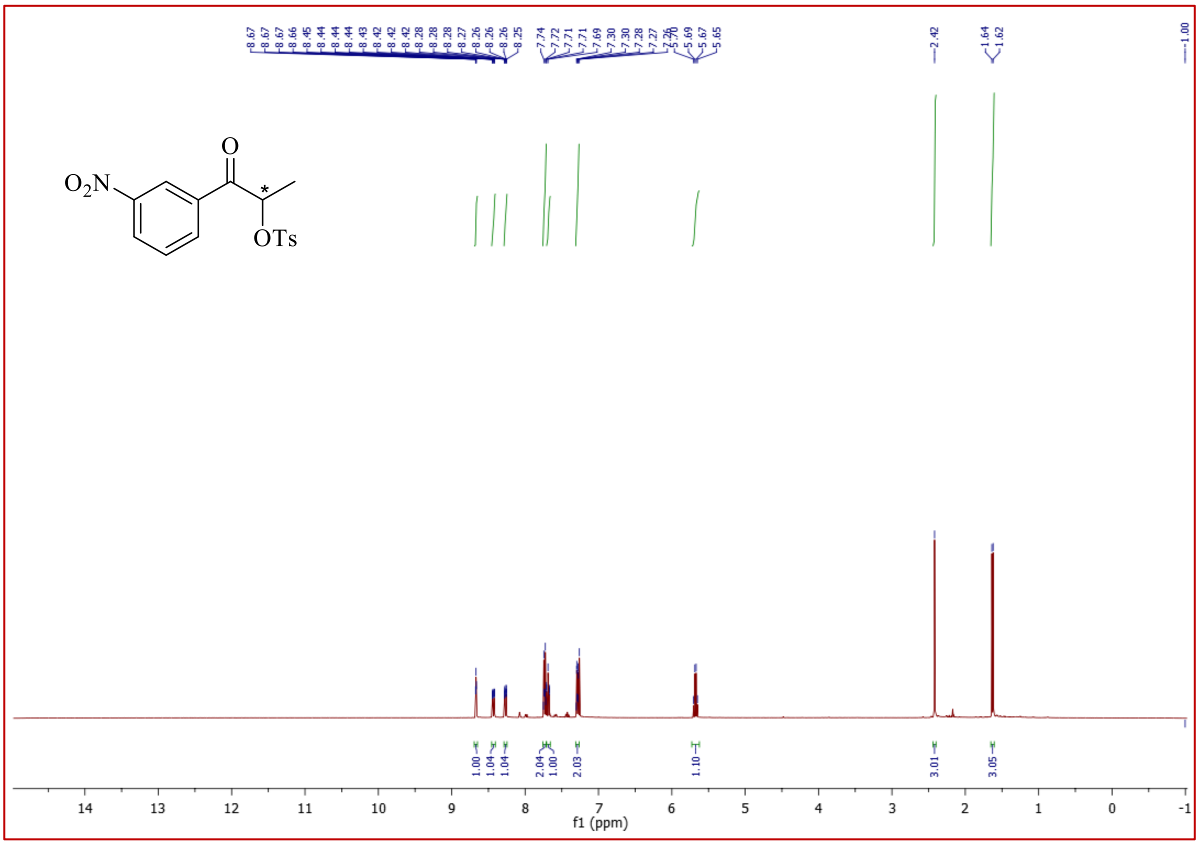

1-(3-Nitrophenyl)-1-oxopropan-2-yl 4-methylbenzenesulfonate (**9e**), ^{13}C NMR

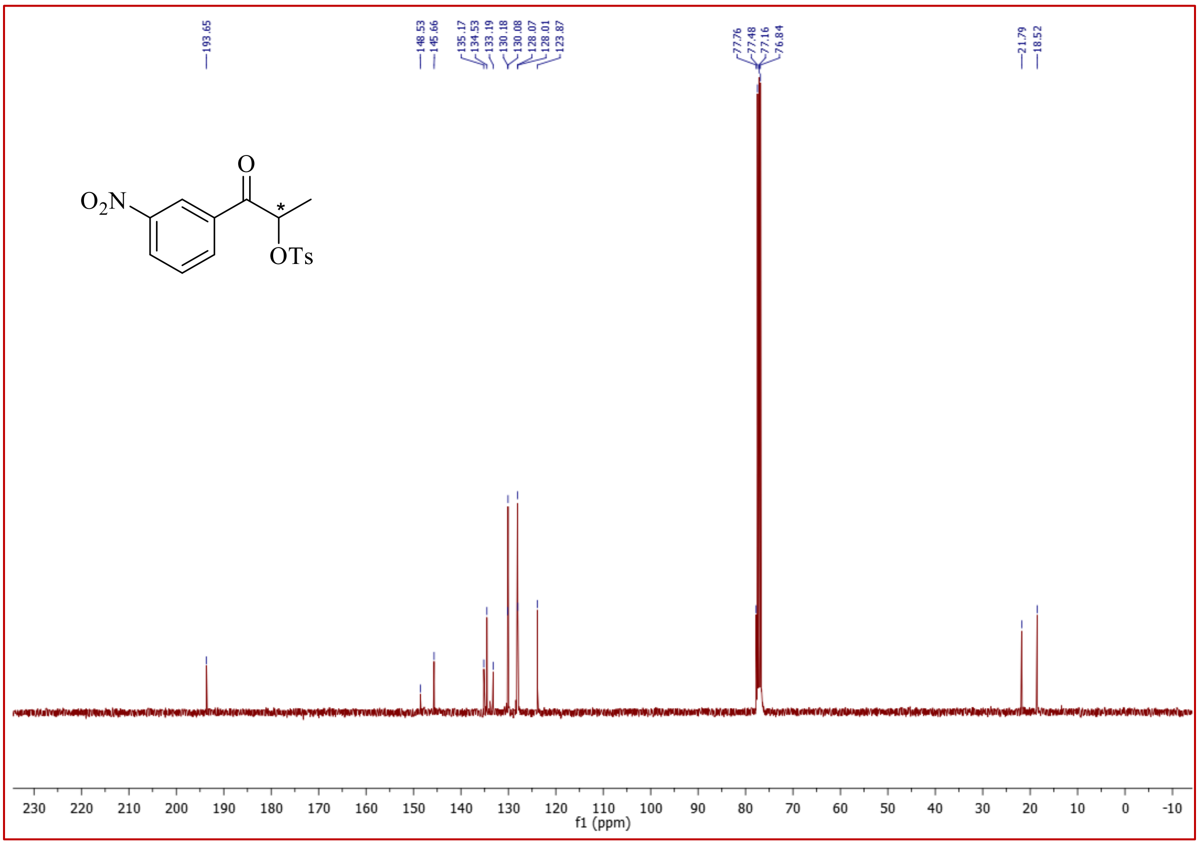

1-Oxo-1-(p-tolyl) propan-2-yl 4-methylbenzenesulfonate (**9f**), ^{1}H NMR

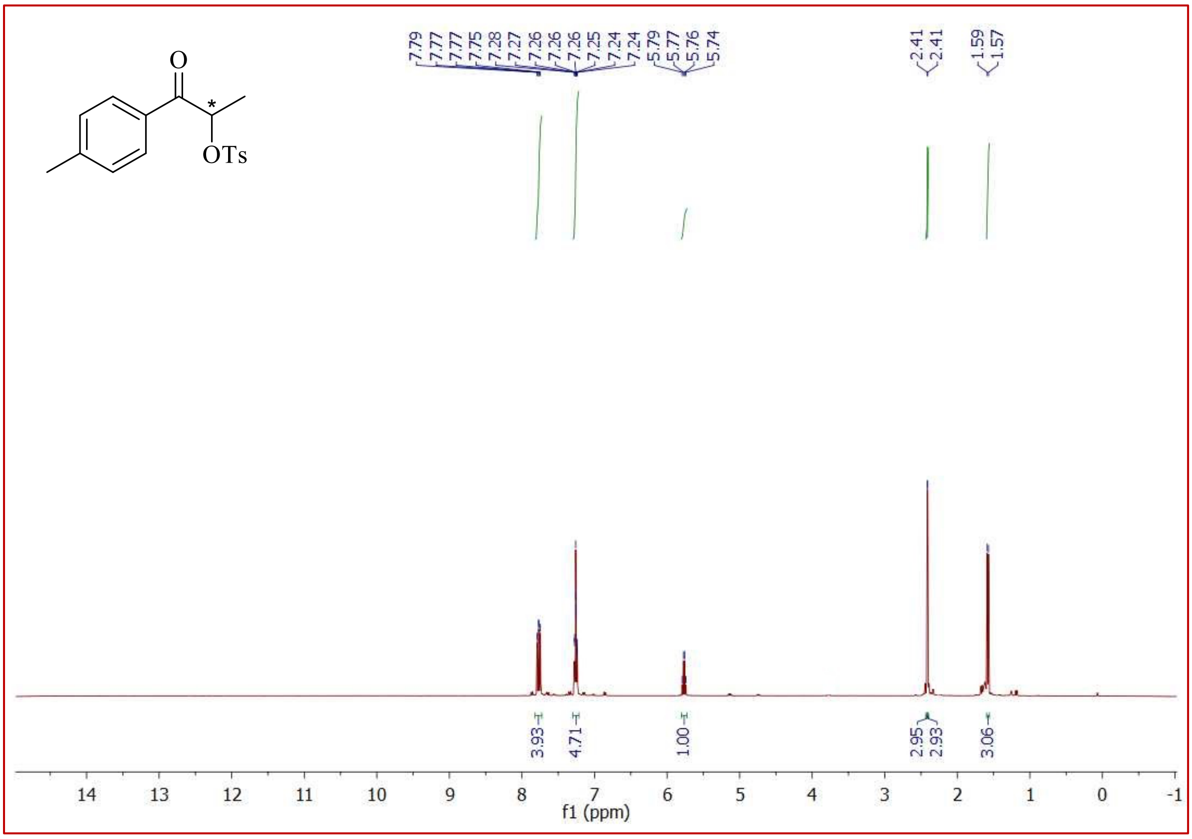

1-Oxo-1-(p-tolyl) propan-2-yl 4-methylbenzenesulfonate (**9f**), ^{13}C NMR

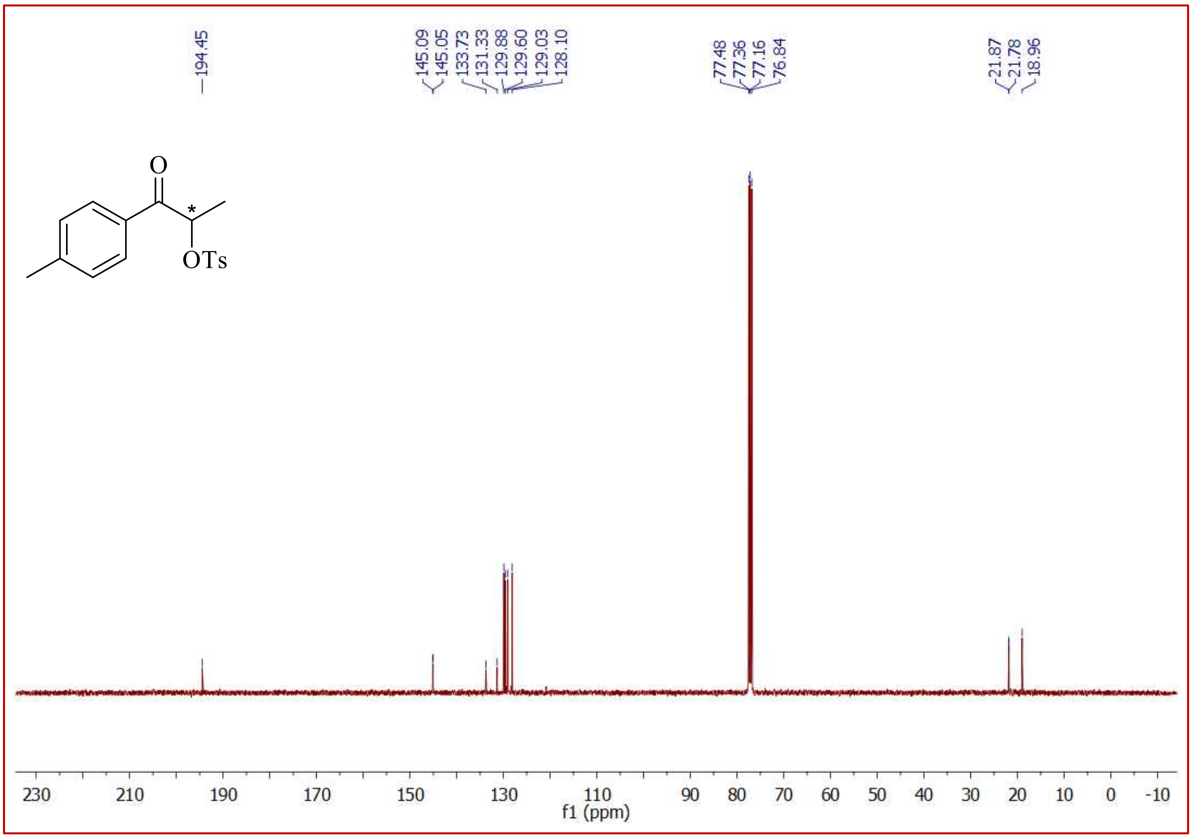

1-(4-Methoxyphenyl)-1-oxopropan-2-yl 4-methylbenzenesulfonate (**9g**), ^{1}H NMR

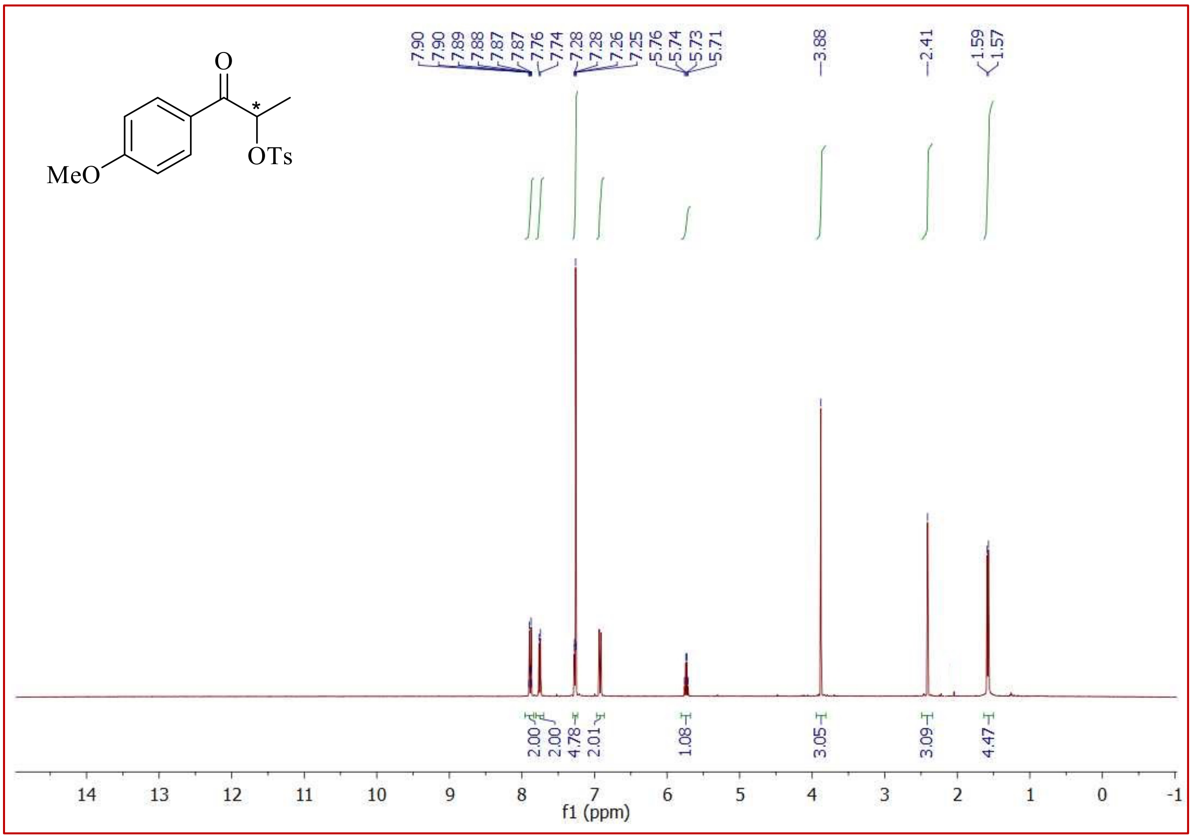

1-(4-Methoxyphenyl)-1-oxopropan-2-yl 4-methylbenzenesulfonate (**9g**), ^{13}C NMR

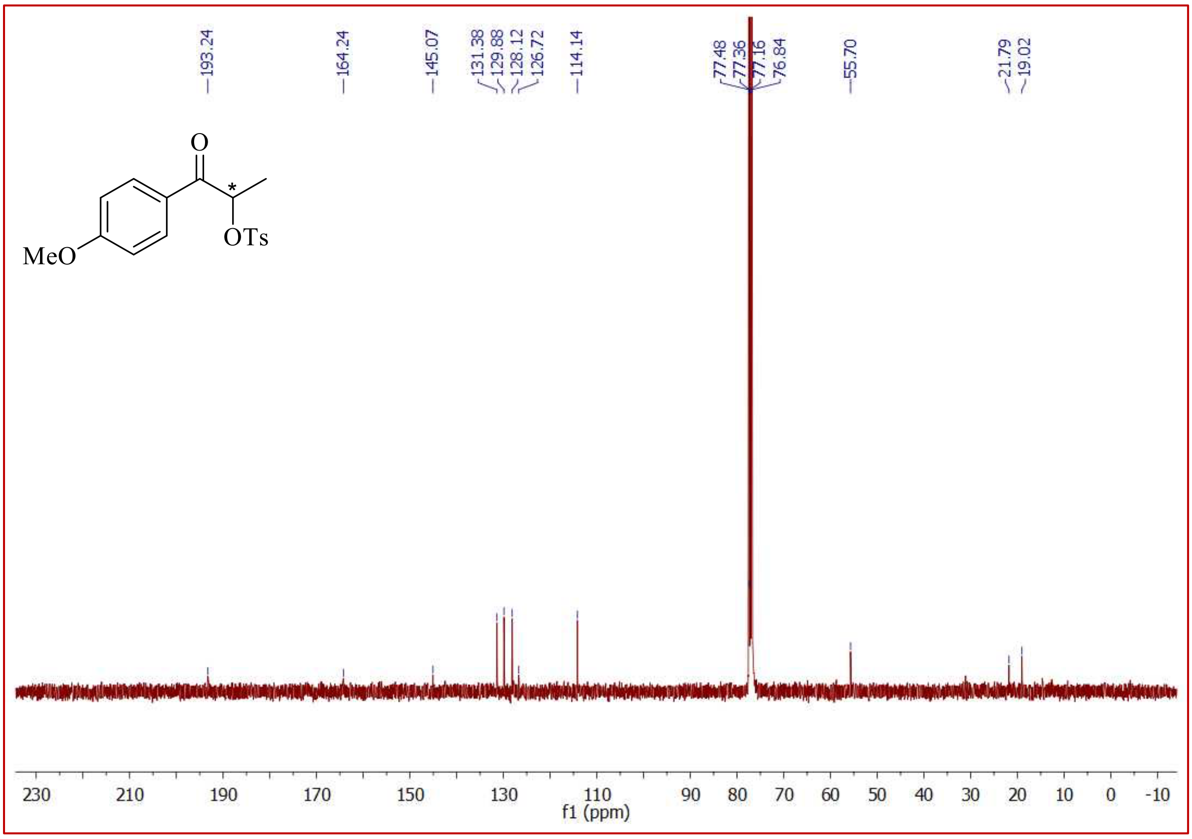

1-(4-(*tert*-Butyl)phenyl)-1-oxopropan-2-yl 4-methylbenzenesulfonate (**9h**), ^{1}H NMR

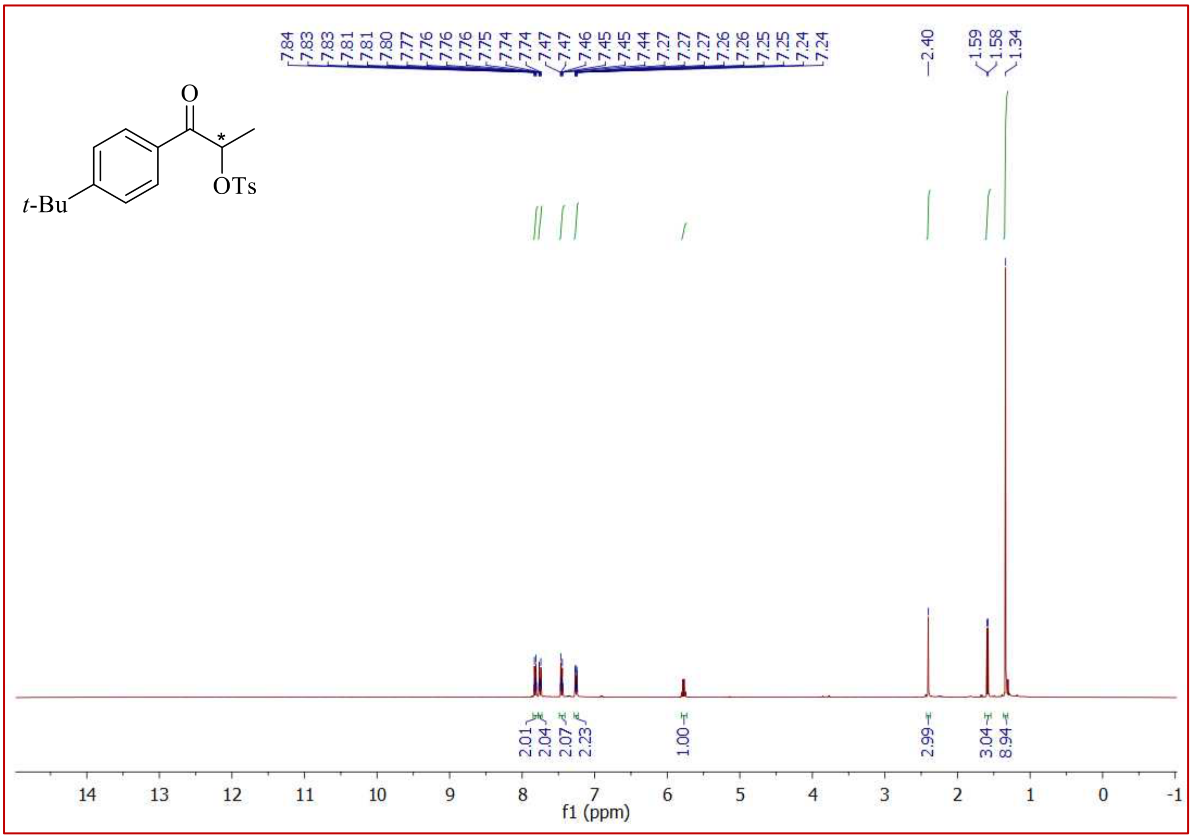

1-(4-(*tert*-Butyl)phenyl)-1-oxopropan-2-yl 4-methylbenzenesulfonate (**9h**), ^{13}C NMR

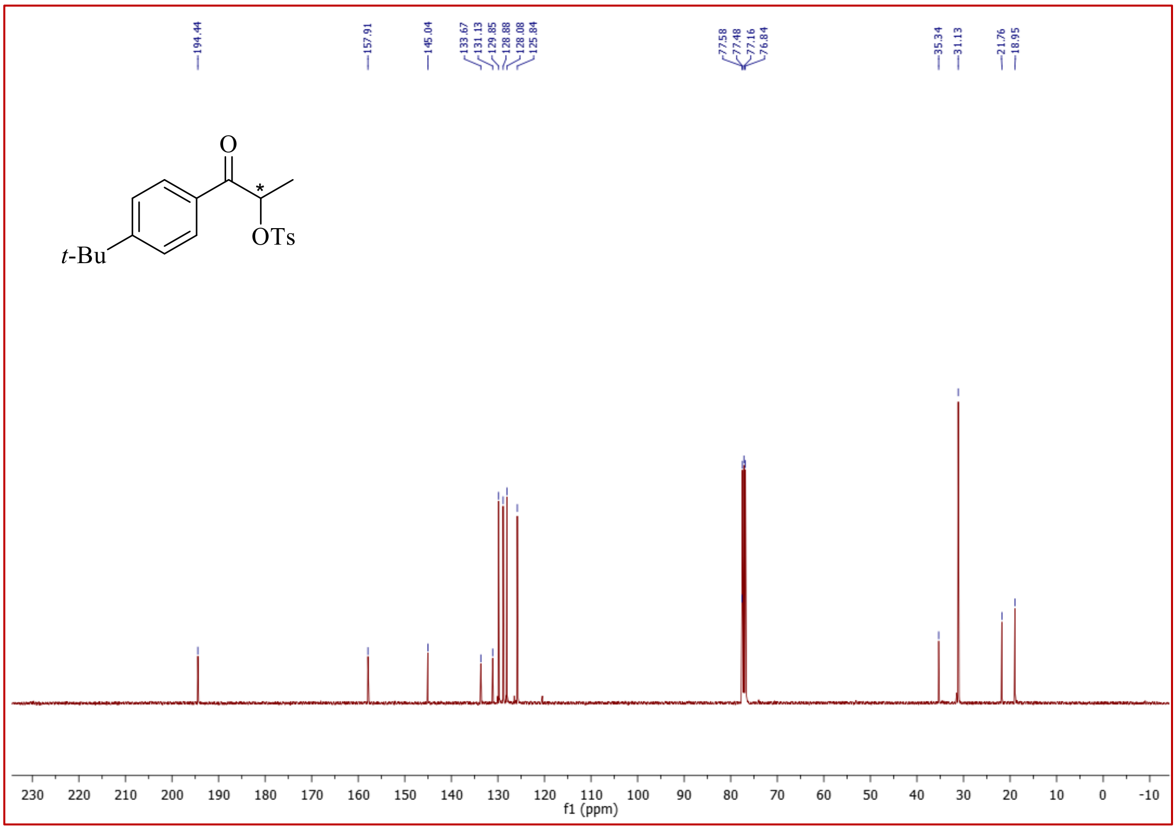

1-(Naphthalen-2-yl)-1-oxopropan-2-yl 4-methylbenzenesulfonate (**9i**), ^{1}H NMR

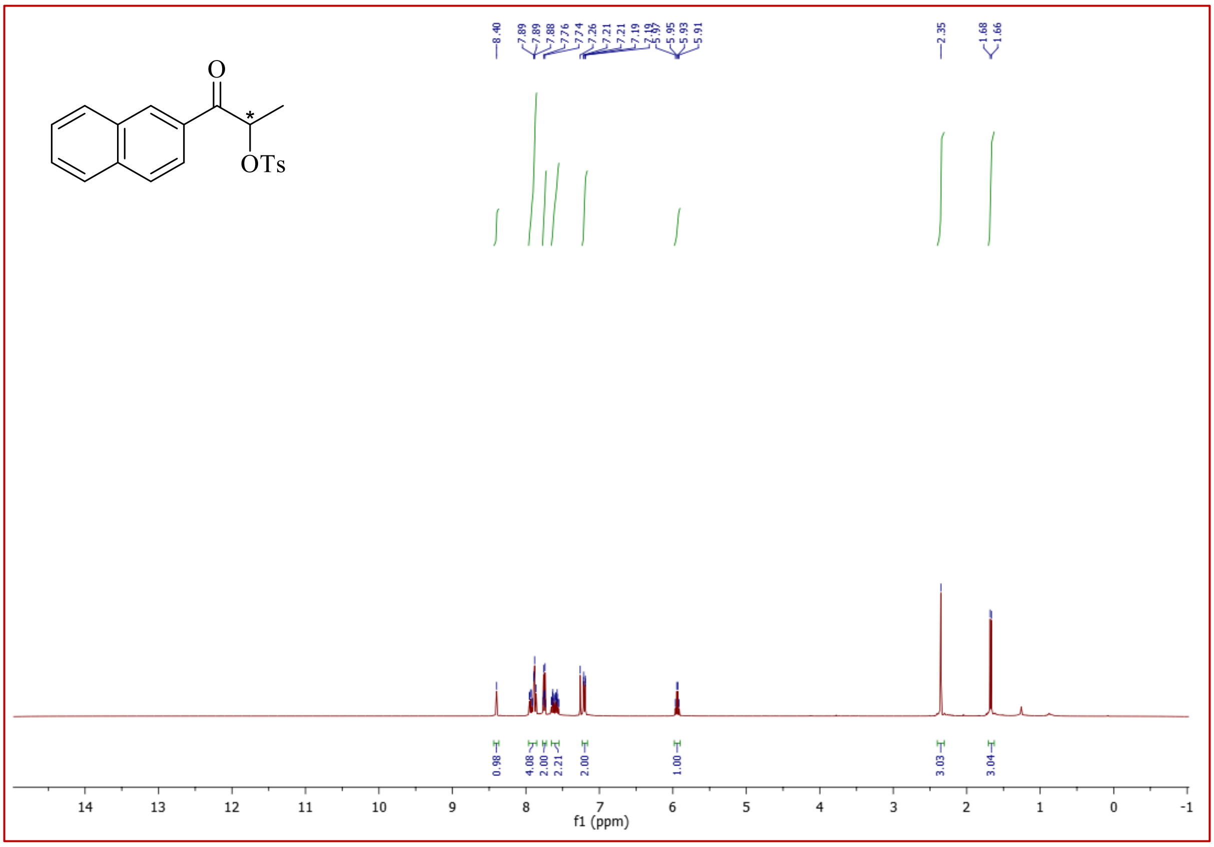

1-(Naphthalen-2-yl)-1-oxopropan-2-yl 4-methylbenzenesulfonate (**9i**), ^{13}C NMR

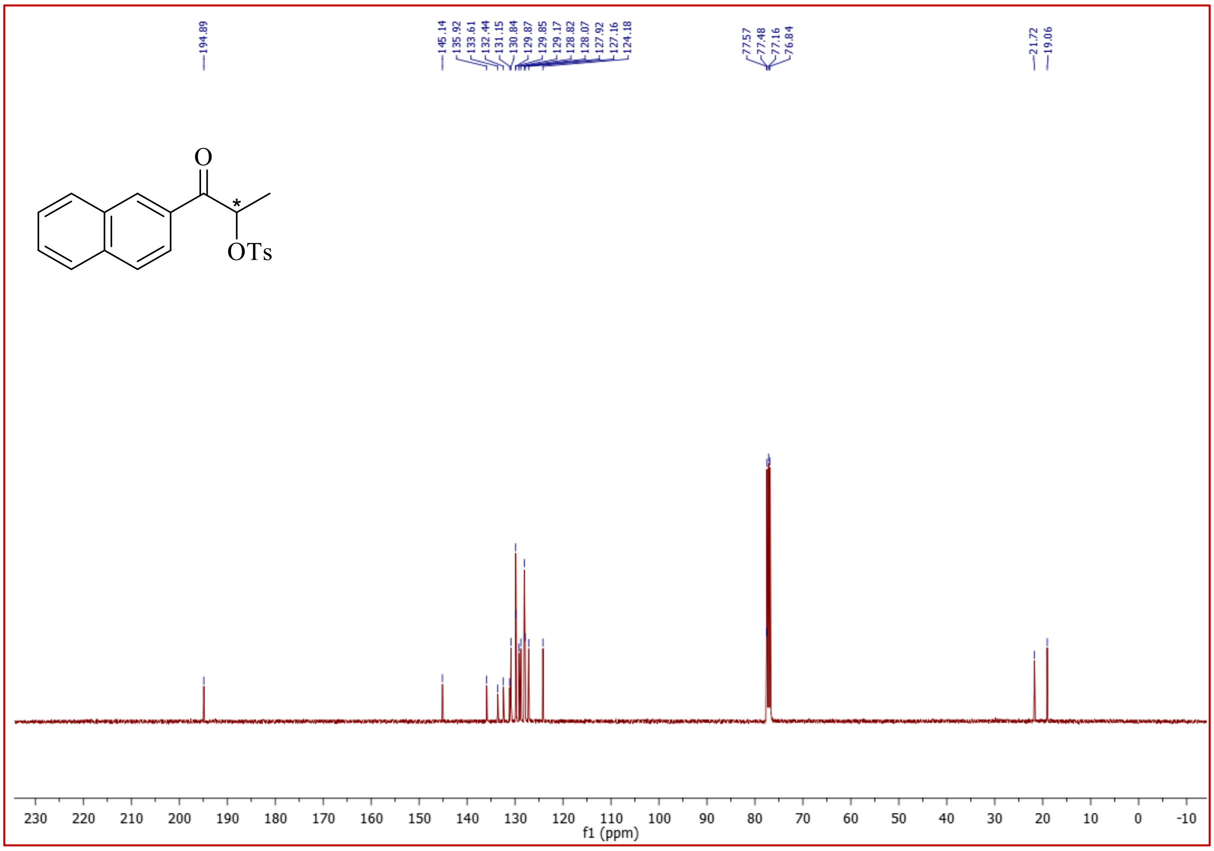

1-Oxo-1-phenylbutan-2-yl 4-methylbenzenesulfonate (**9j**), ^{1}H NMR

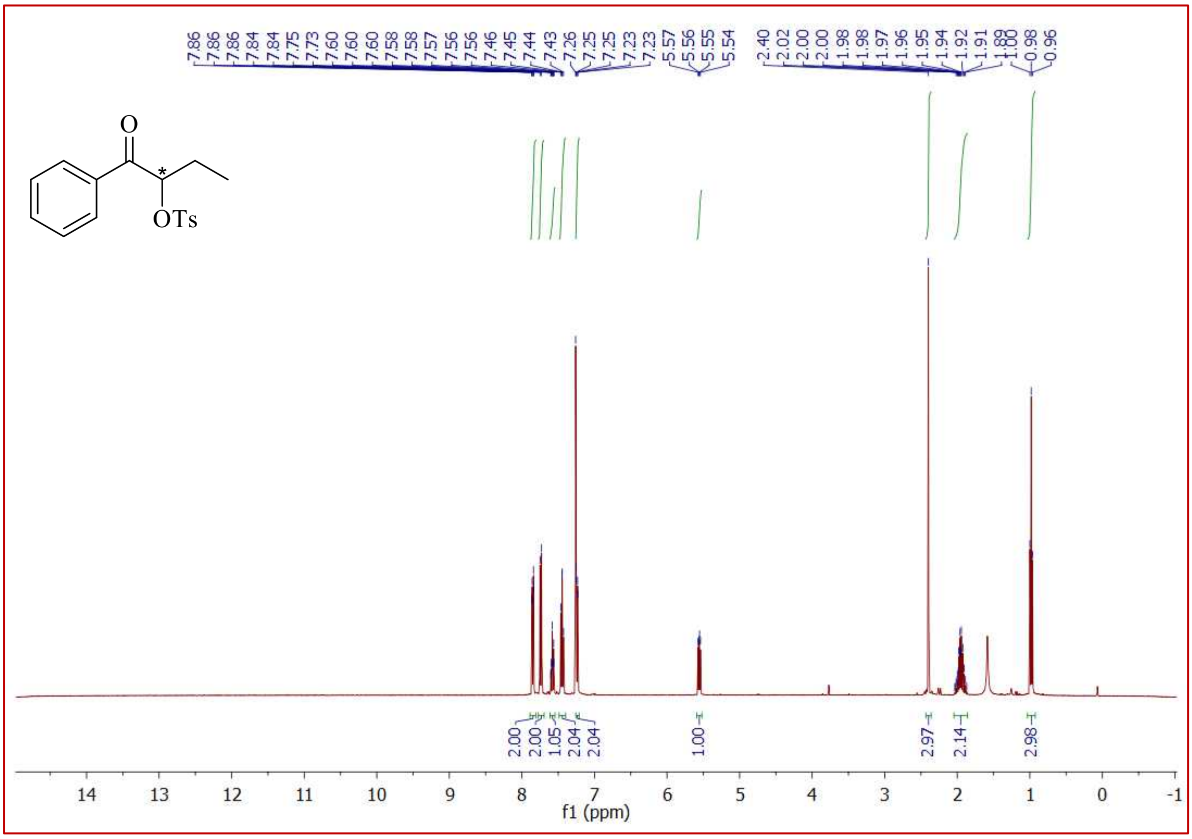

1-Oxo-1-phenylbutan-2-yl 4-methylbenzenesulfonate (**9j**), ^{13}C NMR

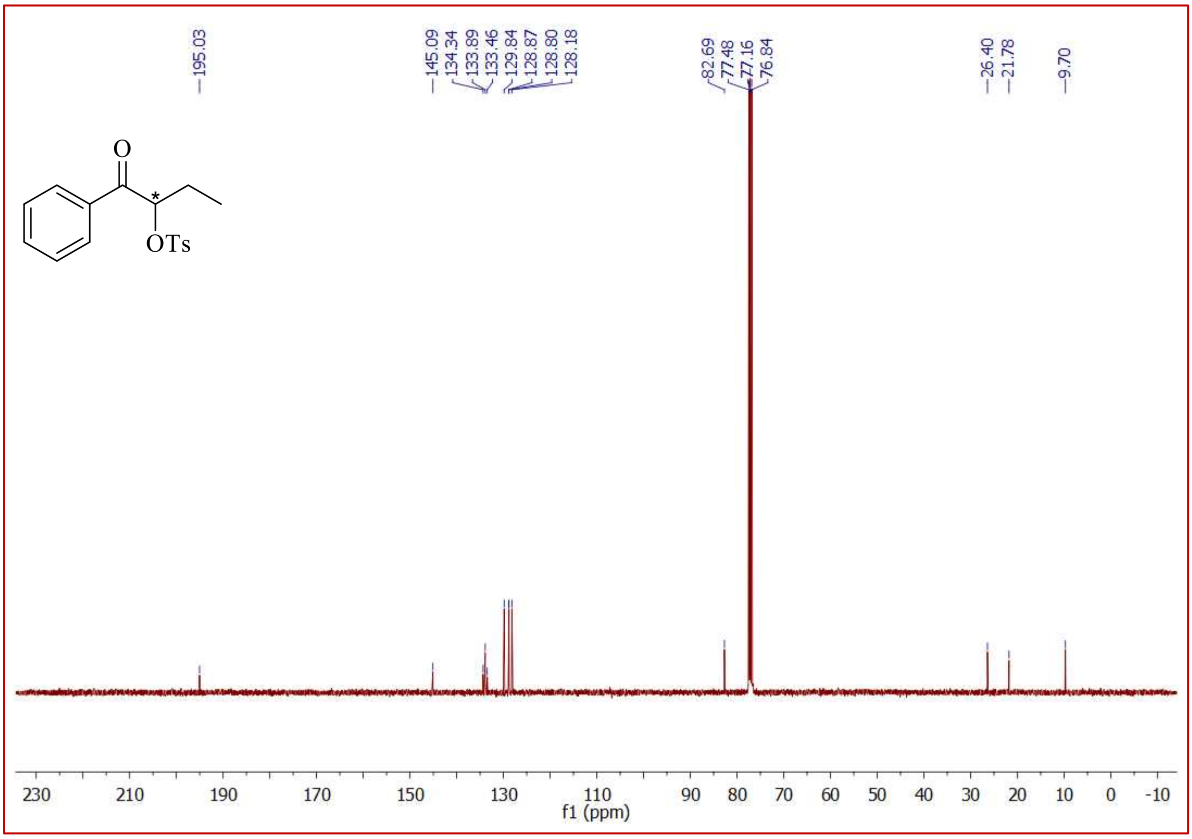

2-Oxo-1,2-diphenylethyl 4-methylbenzenesulfonate (**9k**), ^{1}H NMR

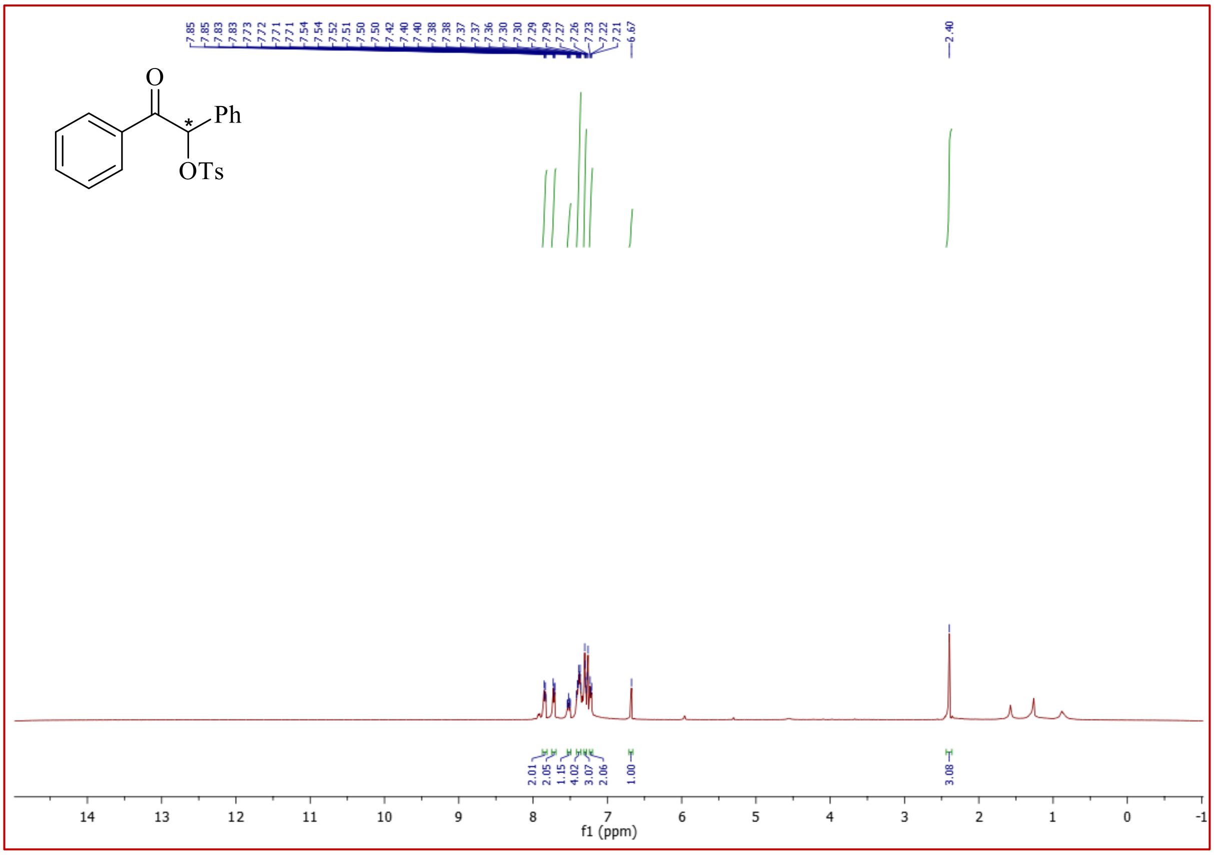

2-Oxo-1,2-diphenylethyl 4-methylbenzenesulfonate (**9k**), ^{13}C NMR

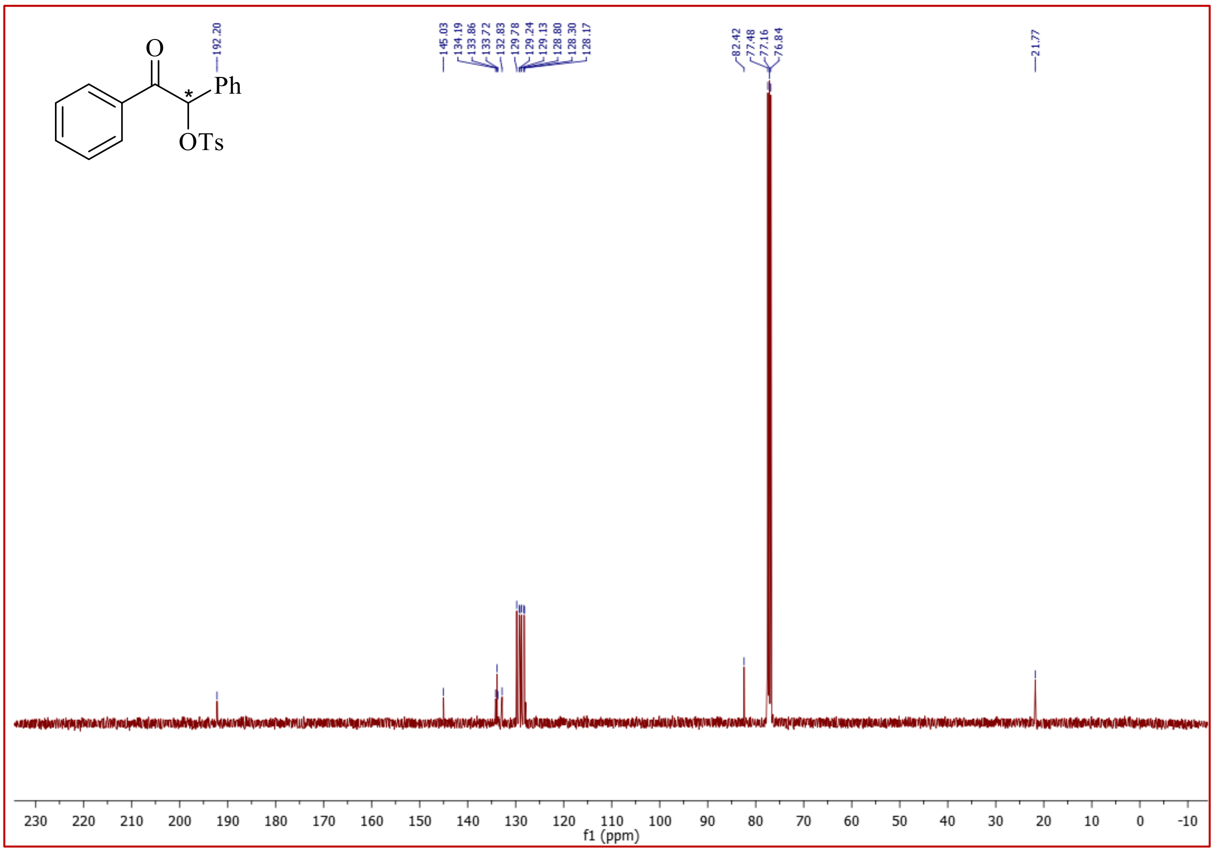

1-(Furan-2-yl)-1-oxopropan-2-yl 4-methylbenzenesulfonate (**9l**), ^{1}H NMR

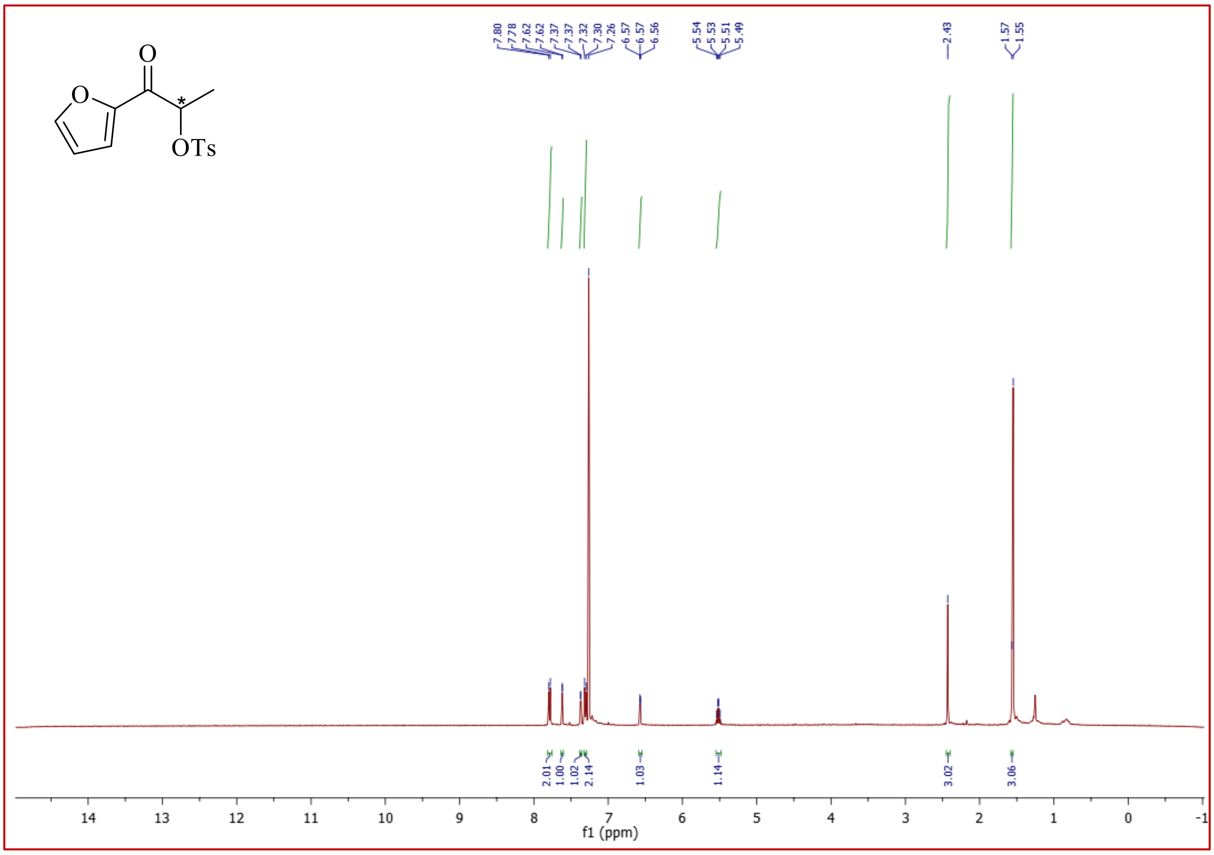

1-(Furan-2-yl)-1-oxopropan-2-yl 4-methylbenzenesulfonate (**9l**), ^{13}C NMR

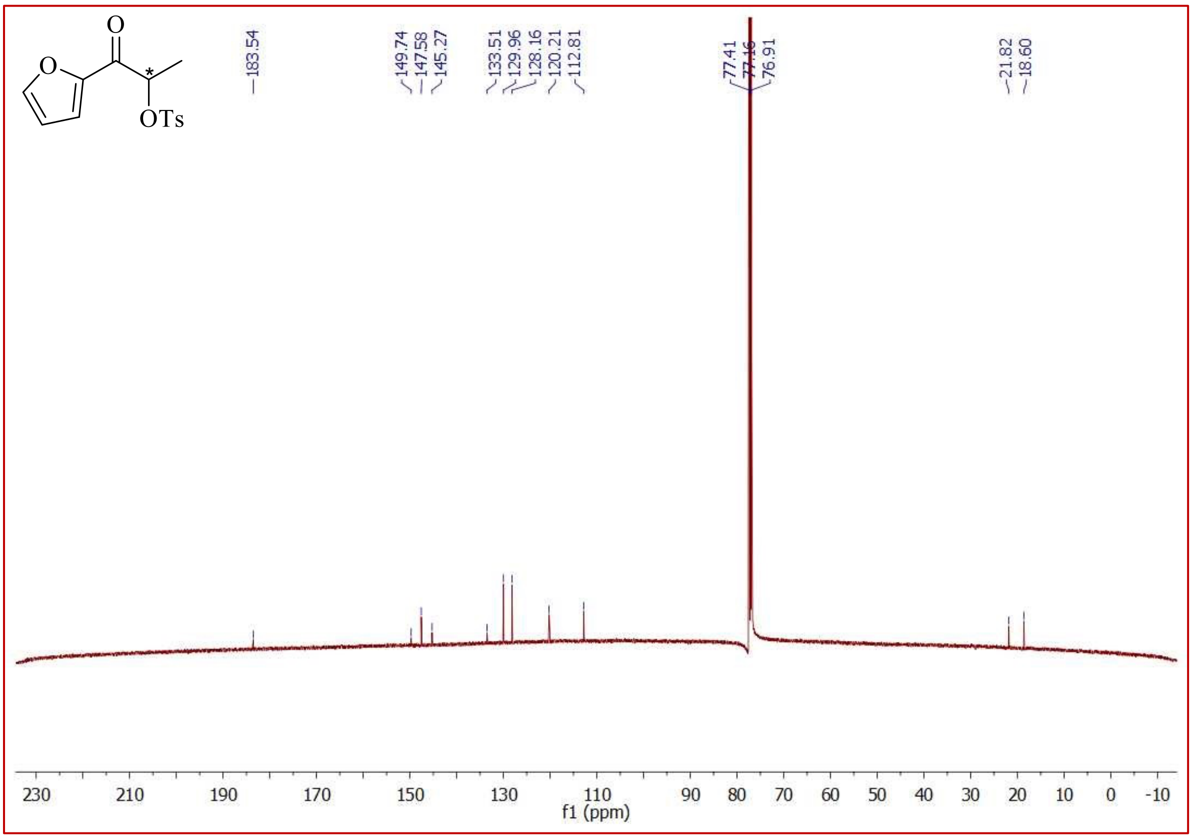

1-Oxo-1-(thiophen-2-yl)propan-2-yl 4-methylbenzenesulfonate (**9m**), ^{1}H NMR

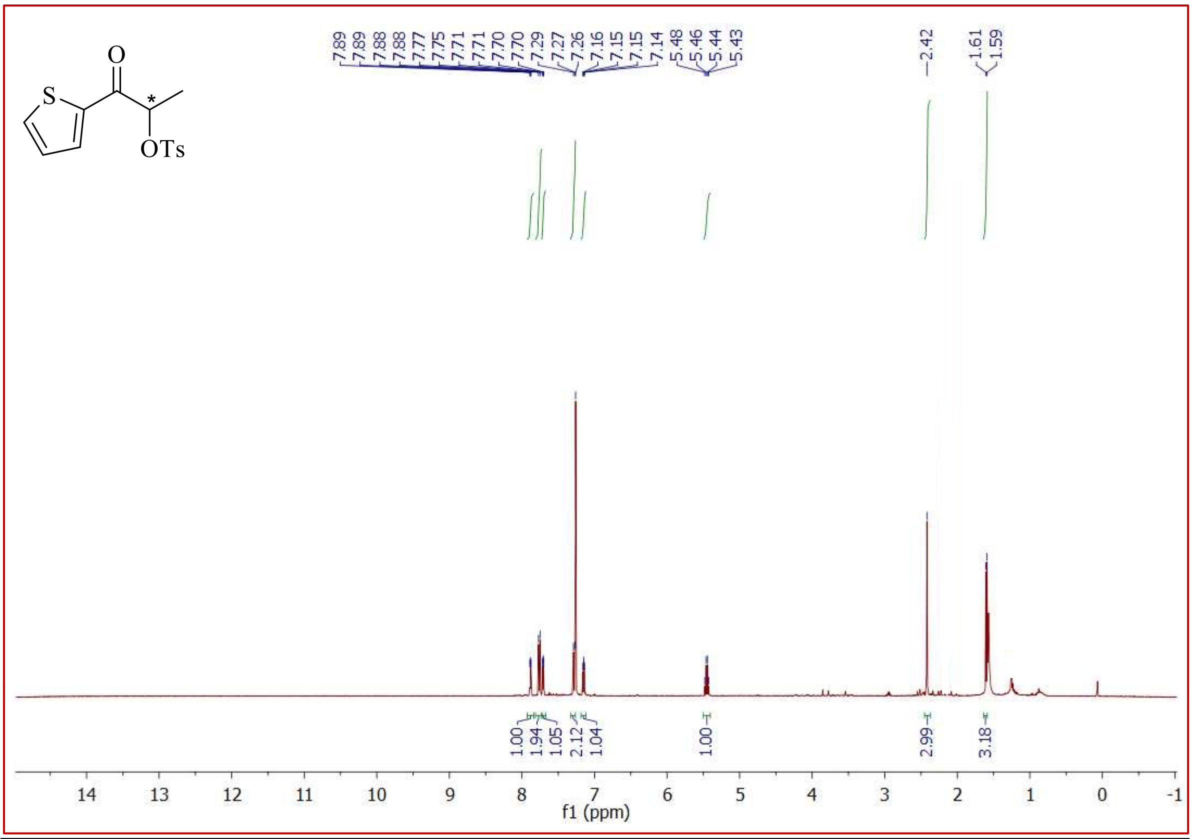

1-Oxo-1-(thiophen-2-yl)propan-2-yl 4-methylbenzenesulfonate (**9m**), ^{13}C NMR

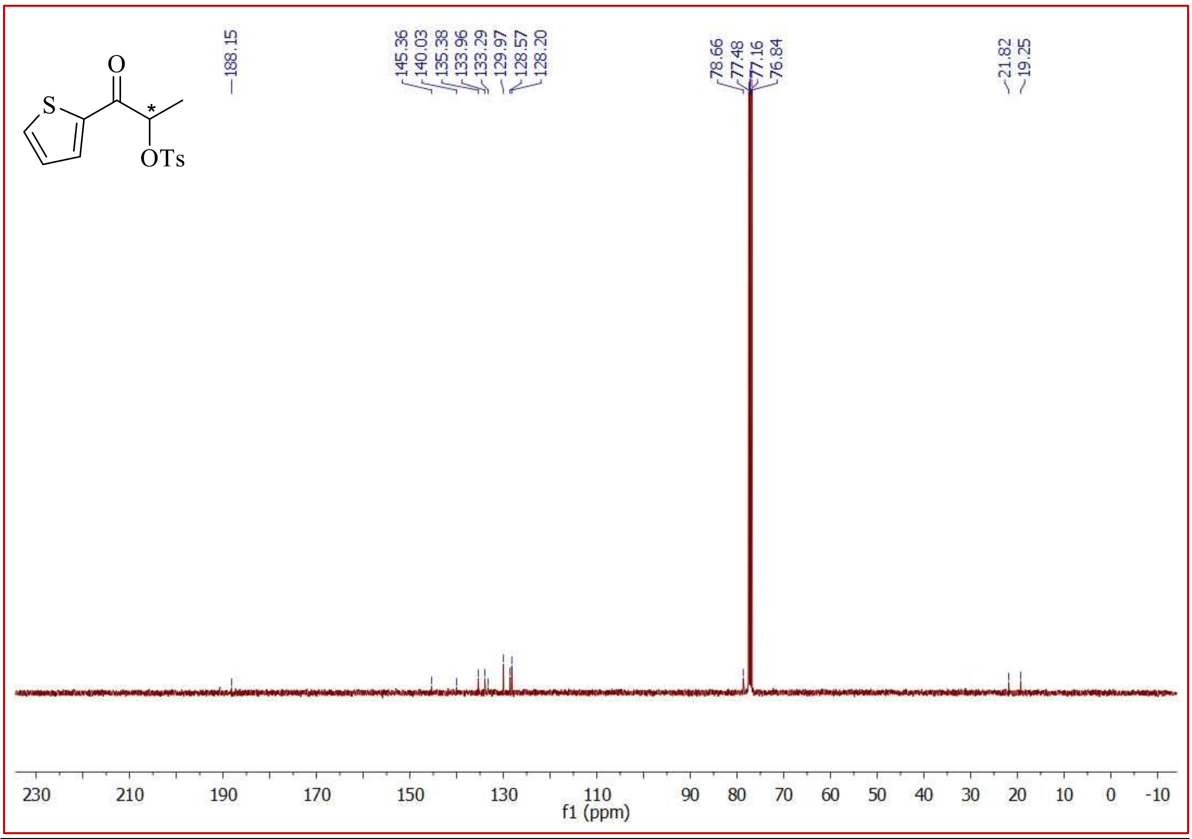

1-Oxo-2,3-dihydro-1H-inden-2-yl 4-methylbenzenesulfonate (**9n**), ^{1}H NMR

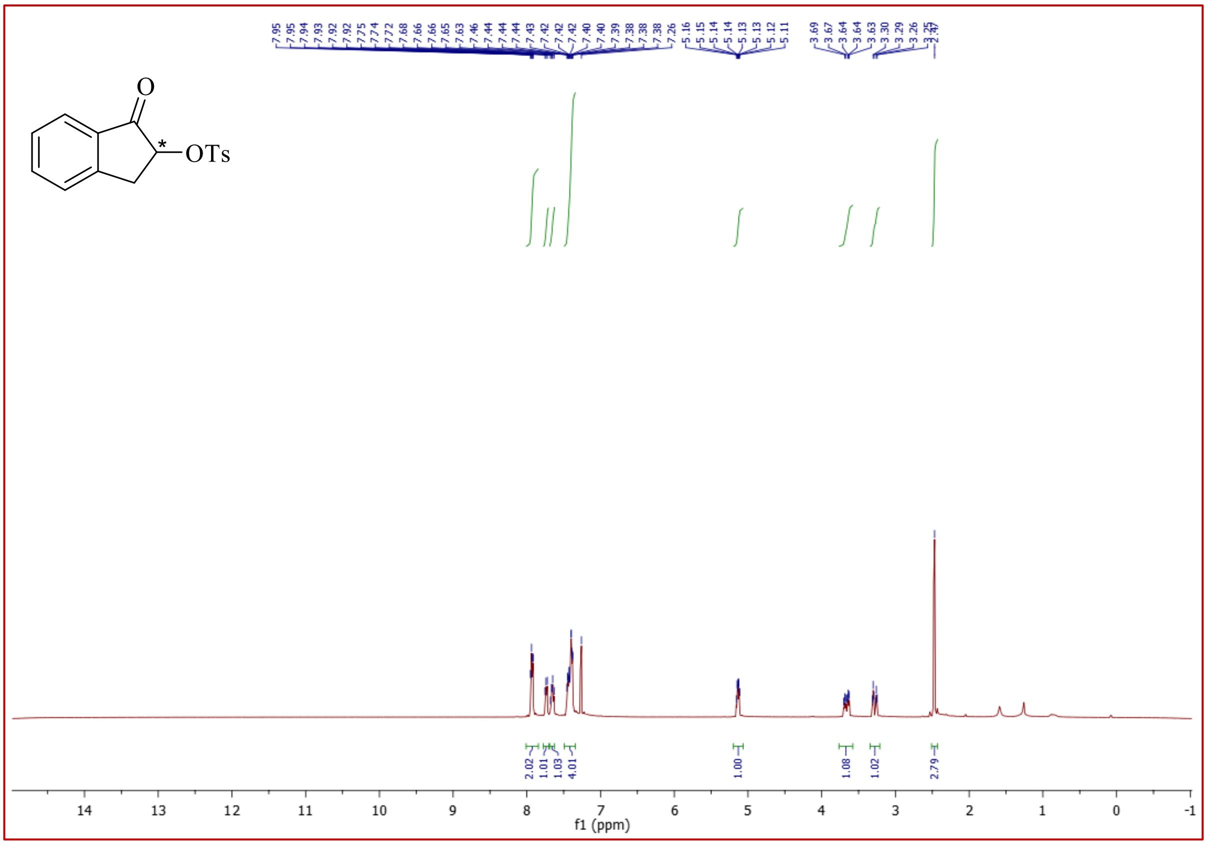

1-Oxo-2,3-dihydro-1H-inden-2-yl 4-methylbenzenesulfonate (**9n**), ^{13}C NMR

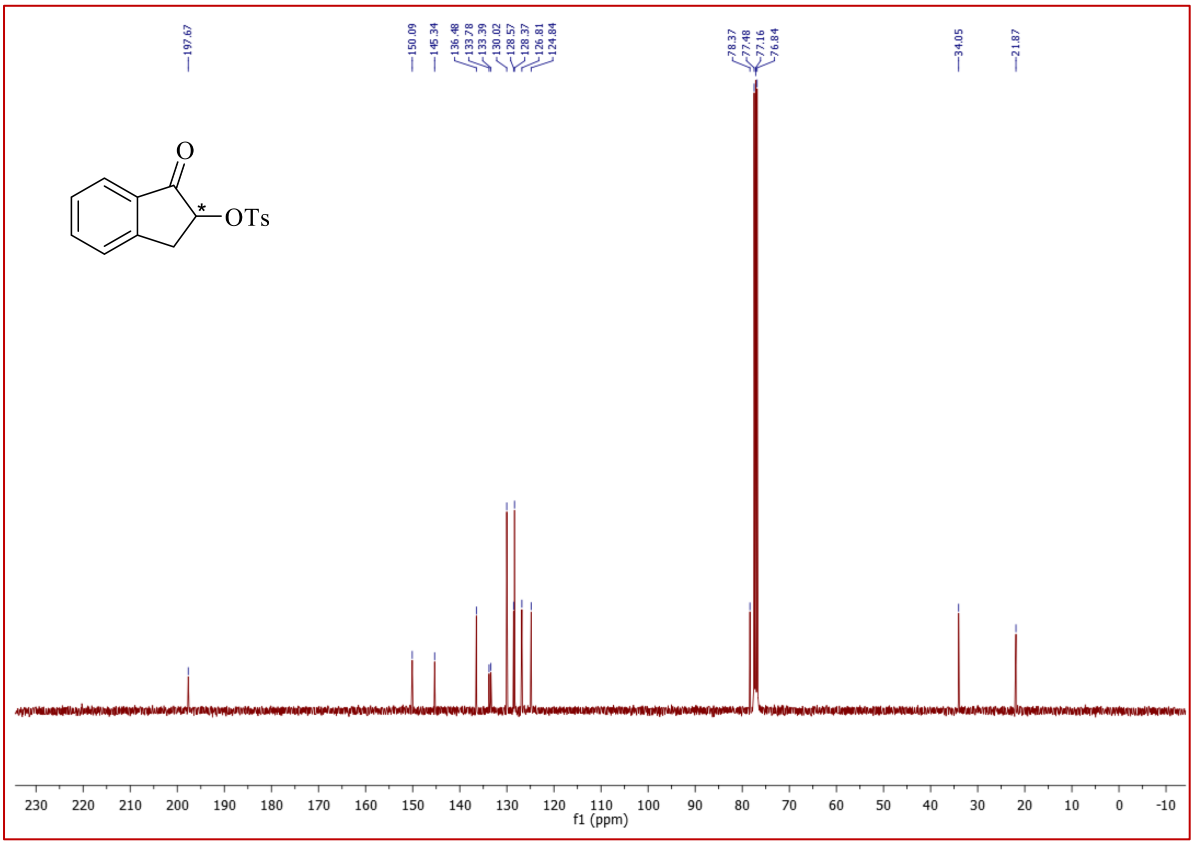

1-Oxo-1,2,3,4-tetrahydronaphthalen-2-yl 4-methylbenzenesulfonate (**9o**), ^{1}H NMR

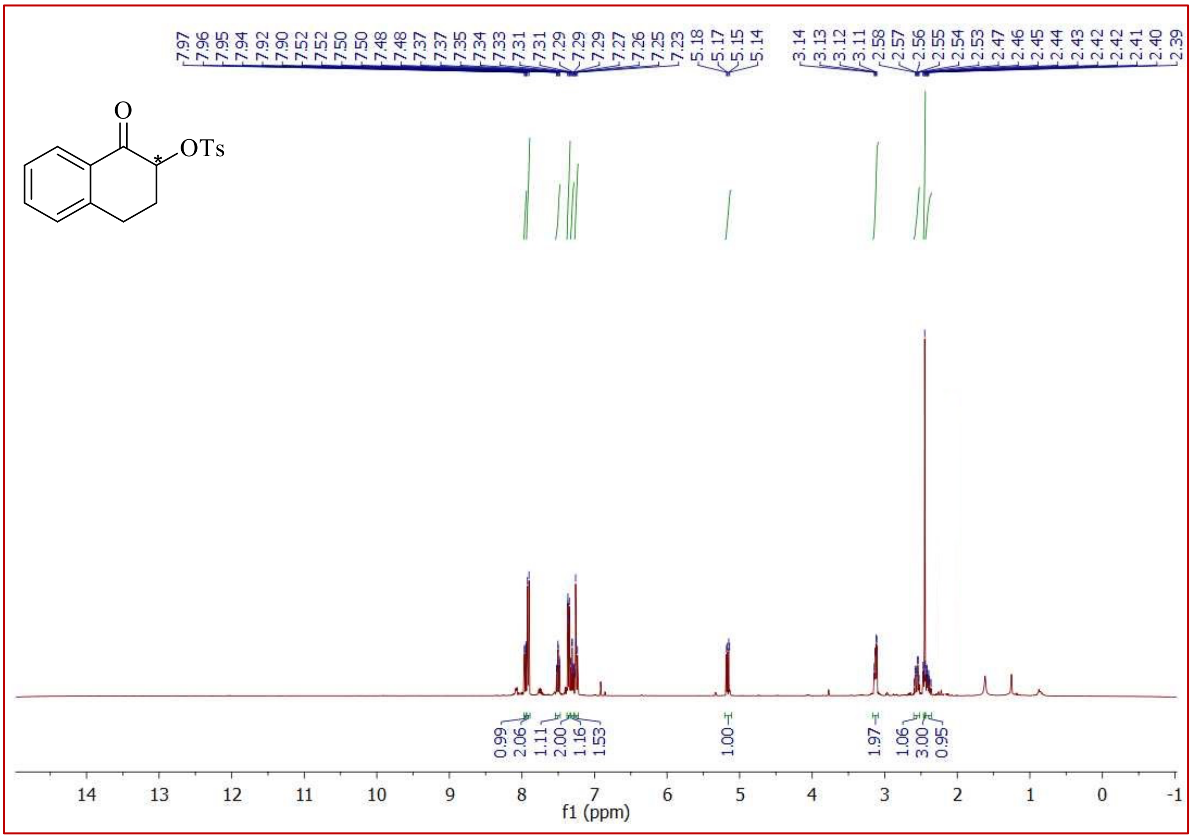

1-Oxo-1,2,3,4-tetrahydronaphthalen-2-yl 4-methylbenzenesulfonate (**9o**), ^{13}C NMR

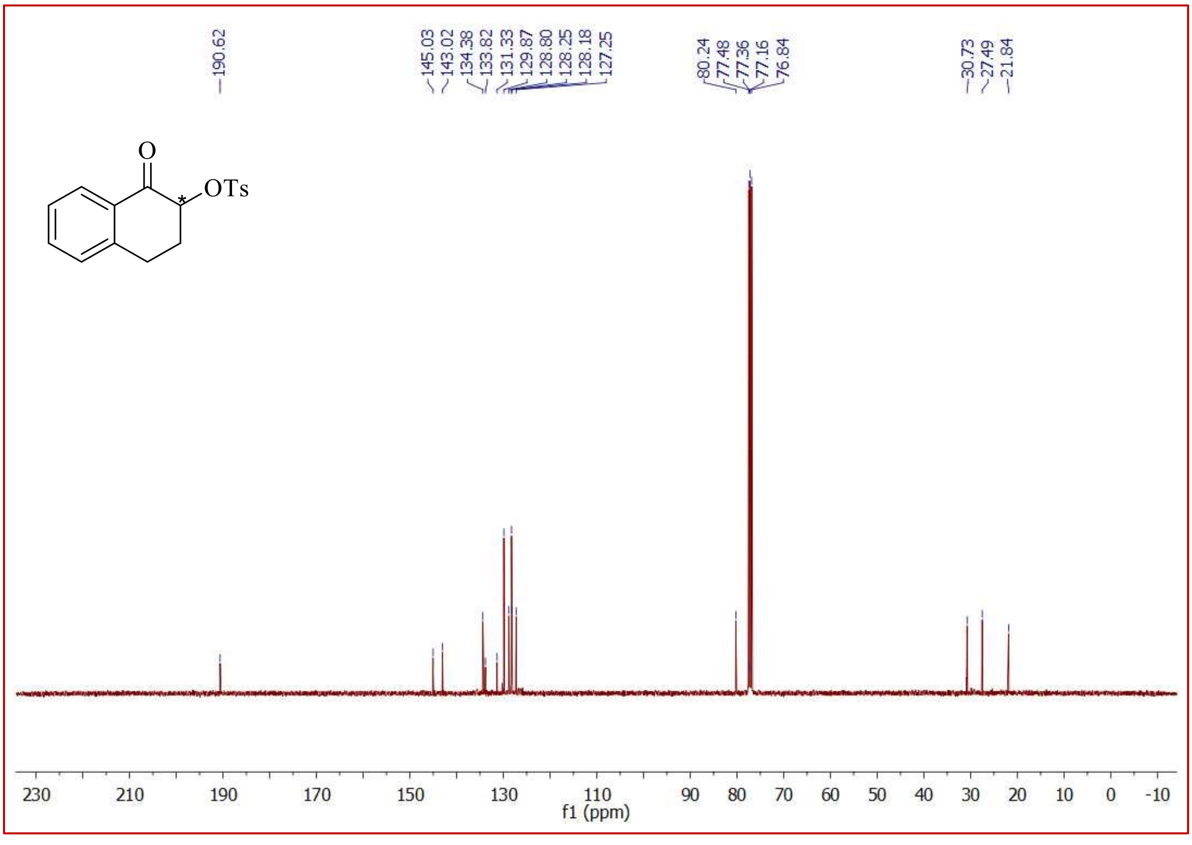

1-Oxo-1-phenylpropan-2-yl benzenesulfonate (**9q**), ^{1}H NMR

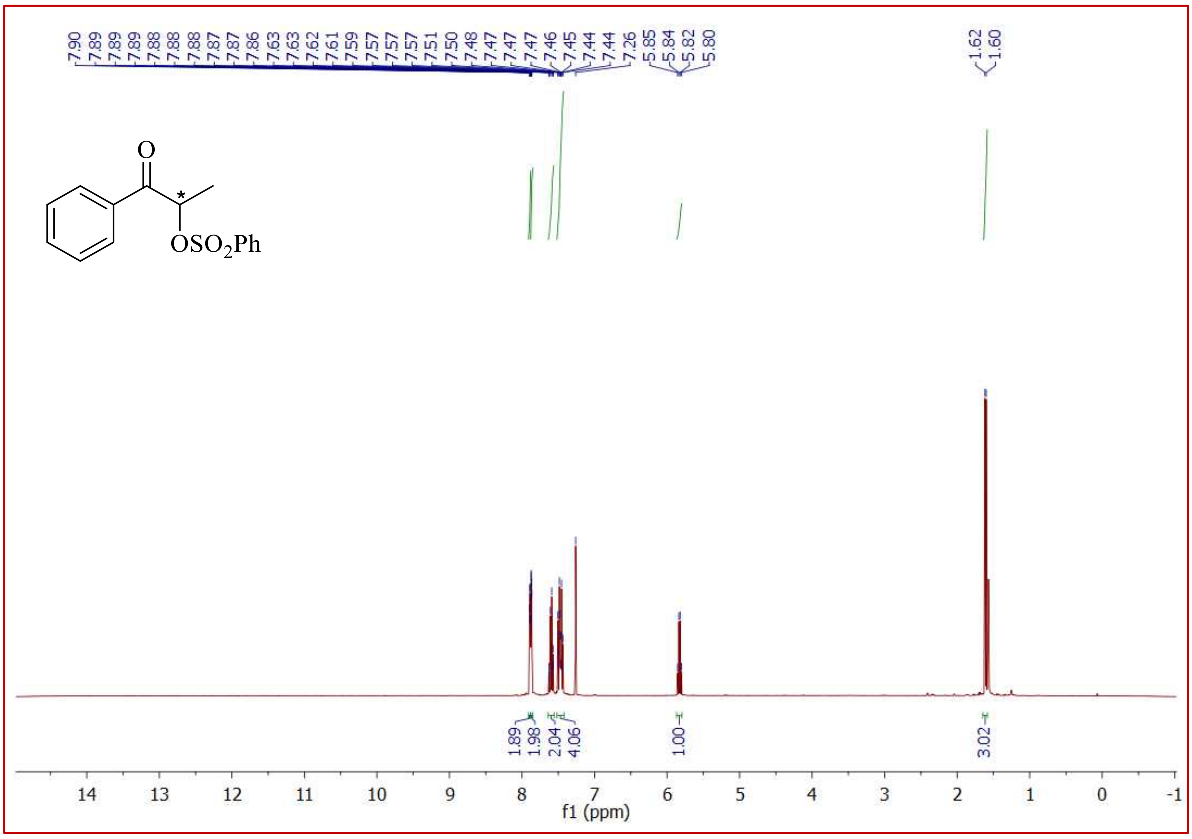

1-Oxo-1-phenylpropan-2-yl benzenesulfonate (**9q**), ^{13}C NMR

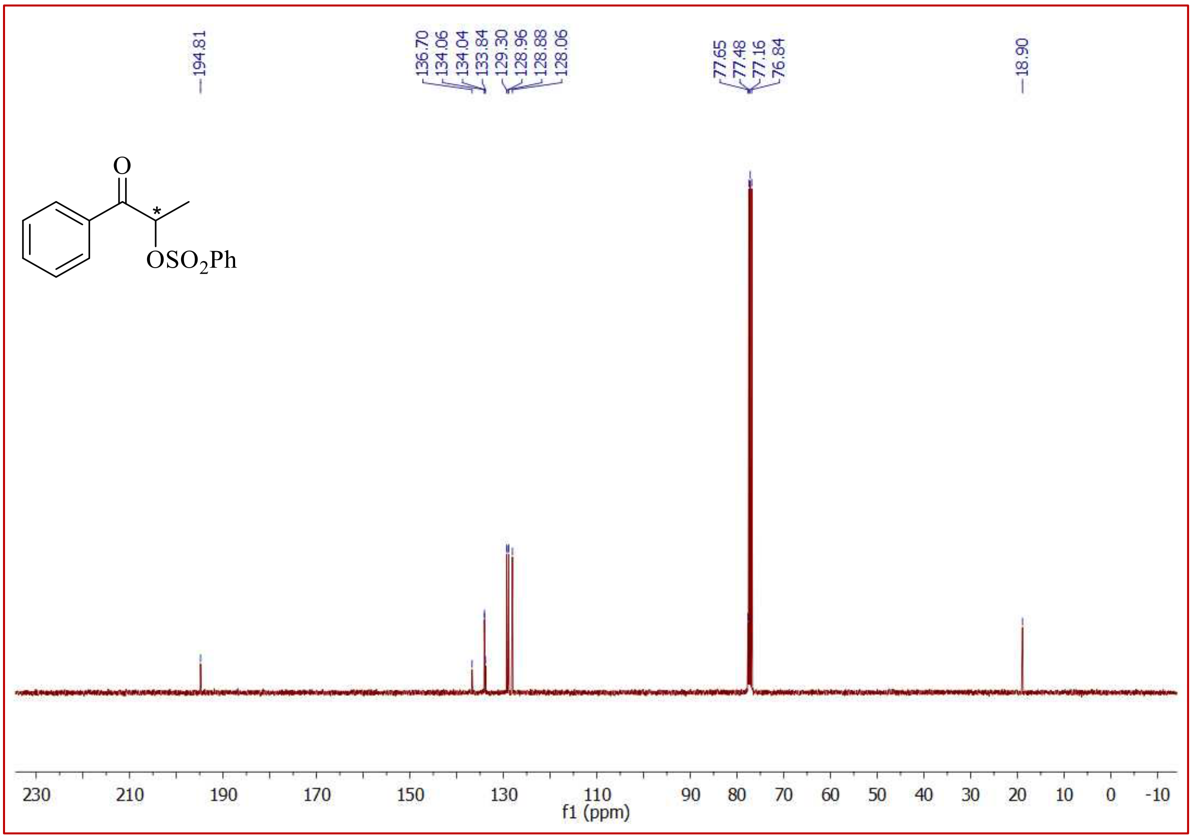

1-Oxo-1-phenylpropan-2-yl methanesulfonate (**9r**), ^{1}H NMR

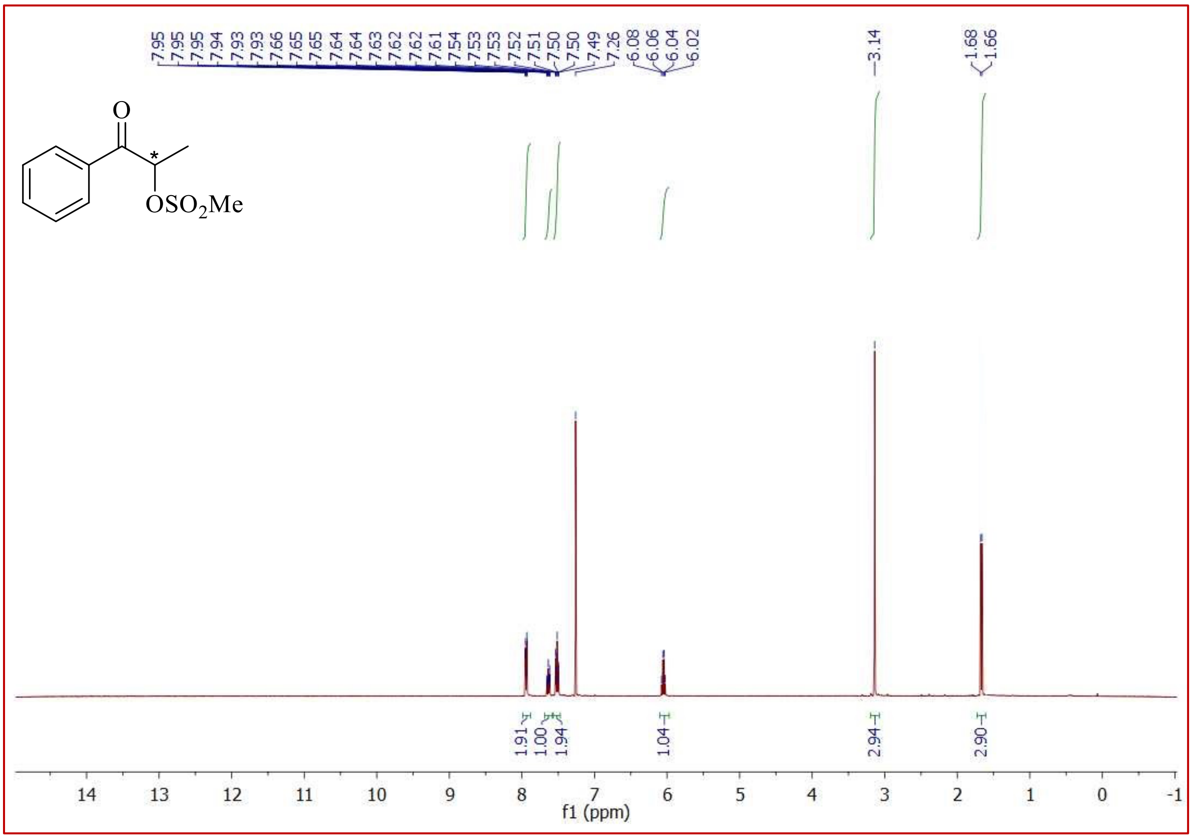

1-Oxo-1-phenylpropan-2-yl methanesulfonate (**9r**), ^{13}C NMR

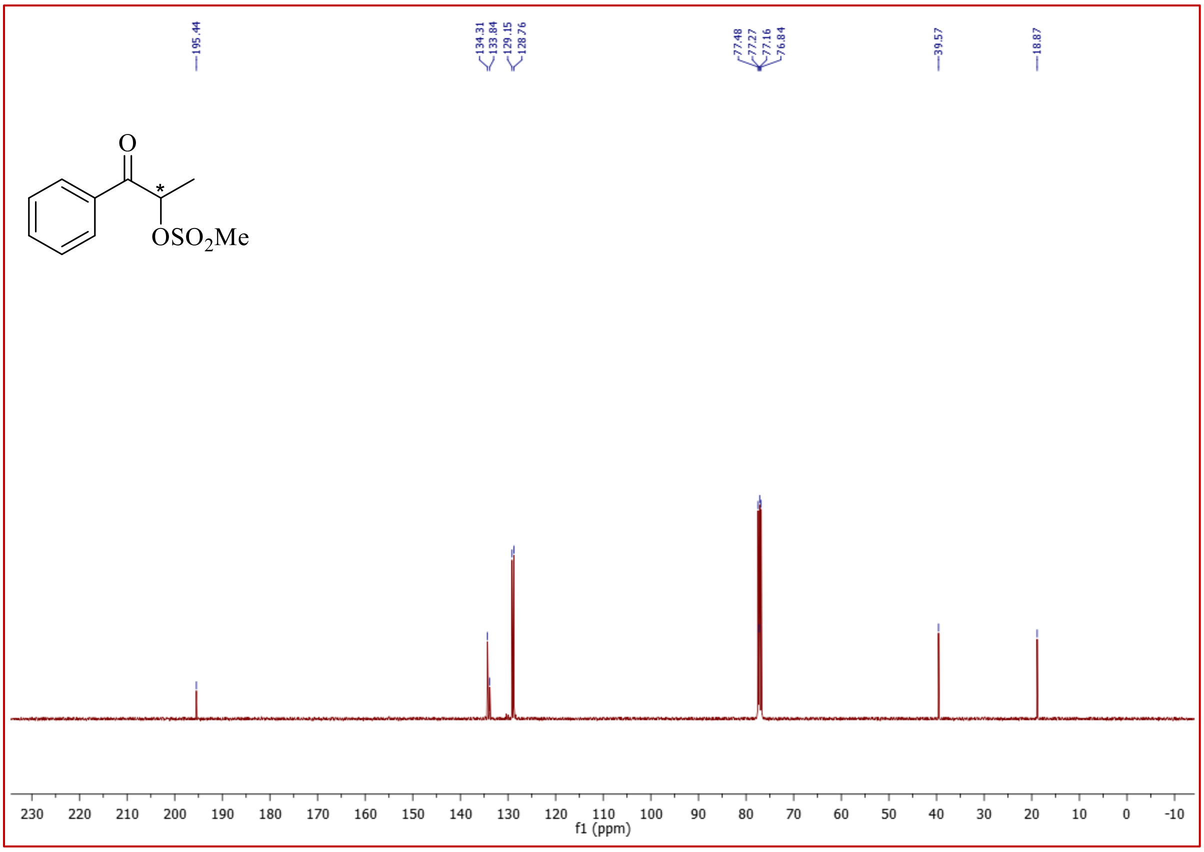

Supplement: Supplementary file 1 — Supplementary [file CHEM-27-4317-s001.pdf]
